# Assessment of the genetic and clinical determinants of hip fracture risk: Genome-wide association and Mendelian randomization study

**DOI:** 10.1016/j.xcrm.2022.100776

**Published:** 2022-10-18

**Authors:** Maria Nethander, Eivind Coward, Ene Reimann, Louise Grahnemo, Maiken E. Gabrielsen, Carl Wibom, Estonian Biobank Research Team, Mari Nelis, Lili Milani, Tõnu Esko, Andres Metspalu, Reedik Mägi, Thomas Funck-Brentano, Mari Hoff, Arnulf Langhammer, Ulrika Pettersson-Kymmer, Kristian Hveem, Claes Ohlsson

**Affiliations:** 1 Department of Internal Medicine and Clinical Nutrition, Institute of Medicine, Sahlgrenska Osteoporosis Centre, Centre for Bone and Arthritis Research at the Sahlgrenska Academy, University of Gothenburg, Vita Stråket 11, 41345 Gothenburg, Sweden; 2 Bioinformatics Core Facility, Sahlgrenska Academy, University of Gothenburg, Gothenburg, Sweden; 3 K.G. Jebsen Center for Genetic Epidemiology, Department of Public Health and Nursing, NTNU, Norwegian University of Science and Technology, 7491 Trondheim, Norway; 4 Estonian Genome Center, Institute of Genomics, University of Tartu, Riia 23b, 51010 Tartu, Estonia; 5 Department of Radiation Sciences, Oncology, Umea University, Umea, Sweden; 6 Department of Rheumatology, Lariboisière Hospital, INSERM U1132, Université de Paris, Paris, France; 7 Department of Neuromedicine and Movement Science, Norwegian University of Science and Technology, Trondheim, Norway; 8 Department of Rheumatology, St Olavs Hospital, Trondheim, Norway; 9 HUNT Research Centre, Forskningsveien 2, 7600 Levanger, Norway”; 10 Levanger Hospital, Nord-Trøndelag Hospital Trust, Levanger, Norway; 11 Department of Integrative Medical Biology, Clinical Pharmacology, Umea University, Umea, Sweden; 12 Region Västra Götaland, Department of Drug Treatment, Sahlgrenska University Hospital, Gothenburg, Sweden

**Keywords:** hip fracture, mendelian randomization, bone mineral density, genome-wide association study

## Abstract

Hip fracture is the clinically most important fracture, but the genetic architecture of hip fracture is unclear. Here, we perform a large-scale hip fracture genome-wide association study meta-analysis and Mendelian randomization study using five cohorts from European biobanks. The results show that five genetic signals associate with hip fractures. Among these, one signal associates with falls, but not with bone mineral density (BMD), while four signals are in loci known to be involved in bone biology. Mendelian randomization analyses demonstrate a strong causal effect of decreased femoral neck BMD and moderate causal effects of Alzheimer’s disease and having ever smoked regularly on risk of hip fractures. The substantial causal effect of decreased femoral neck BMD on hip fractures in both young and old subjects and in both men and women supports the use of change in femoral neck BMD as a surrogate outcome for hip fractures in clinical trials.

## Highlights

• Five genetic signals associate with hip fractures in a meta-analysis of five cohorts
• Four of the signals associate also with BMD, while the last signal associates with falls
• Decreased femoral neck BMD is a strong causal risk factor for hip fractures
• Ever smoked regularly and Alzheimer’s disease are moderate causal risk factors

## Graphical abstract

**Figure:**
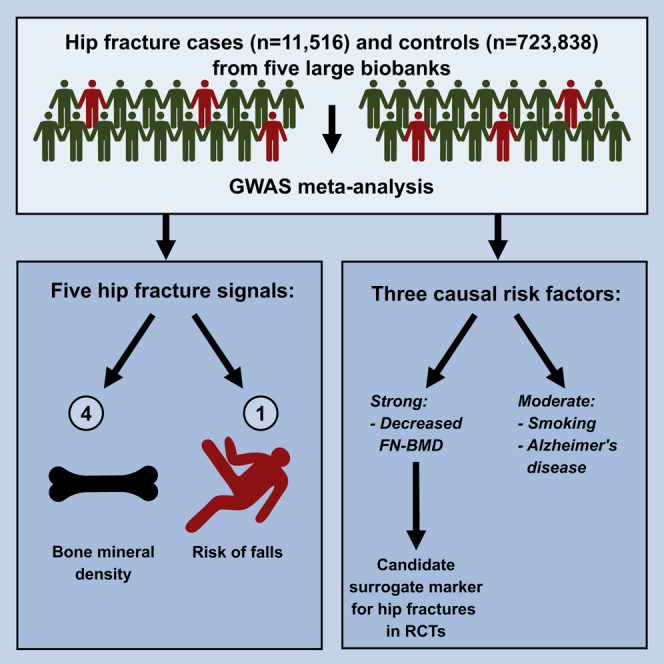

## Teaser

Using five cohorts from European biobanks in a GWAS meta-analysis, Nethander et al. show that five genetic signals associate with hip fractures. For hip fractures, decreased femoral neck BMD is a strong causal risk factor, while ever smoked regularly and Alzheimer’s disease are moderate causal risk factors.

## Introduction

Osteoporosis is a disease characterized by low bone mass and micro-architectural deterioration of bone tissue, leading to increased risk of fragility fractures.^1^ However, fracture risk is not only determined by bone strength but also by the risk of falls that, in turn, is influenced by parameters such as muscle mass and function, balance, medications, and vision.^2^^,^^3^ Hip fracture is the most severe type of fracture, associated with a high morbidity and mortality as well as high costs for society.^4^^,^^5^ The hip fracture incidence increases exponentially by age, and by 2050, the worldwide annual number of hip fractures is expected to reach 4.5 to 6.3 million, reflecting the continuous aging of the population.^4^^,^^6^

The extent to which modification of predictive clinical risk factors reduces hip fracture risk is unknown. A better understanding of causal mechanisms for hip fractures will enable prevention strategies and provide targets for effective lifestyle and pharmacological interventions. Understanding whether interventions aimed at clinical risk factors would reduce fracture risk is important, enabling clinicians to ensure that such risk factors are optimized in individuals at high risk of fracture. If the risk factors are not causal, then such optimization would not decrease fracture risk.^7^ Due to very high costs for randomized controlled osteoporosis registration trials with the required fracture endpoints, no new osteoporosis drugs are currently being evaluated in clinical trials.^8^ The validation of bone mineral density (BMD) change as a surrogate outcome for fracture could reduce the size and costs for randomized controlled osteoporosis registration trials. The use of BMD change as a surrogate outcome for fractures is supported by a recent large meta-analysis revealing that treatment-related BMD changes are strongly associated with fracture reductions across randomized trials of osteoporosis therapies with differing mechanisms of action.^8^ An alternative approach to estimate the usefulness of treatment-related response on femoral neck BMD as a surrogate outcome to specifically reduce hip fractures is to use Mendelian randomization (MR) and determine the causal effect estimates of genetically determined femoral neck BMD on hip fracture risk. However, this requires the availability of a well-powered genome-wide association study (GWAS) on hip fractures.

Twin studies have shown a heritability estimate of 48% for hip fractures.^9^ The heritable component of hip fracture risk may depend on both BMD-related and non-BMD-related genetic determinants. Although the genetic architecture of BMD has recently been evaluated in detail, the genetic determinants of hip fracture risk are essentially unknown.^10^, ^11^, ^12^ Two recent large-scale GWASs on fractures at any bone site, including a mixture of fractures at different bone sites and also including less-validated self-reported fractures, have identified 15 fracture loci.^7^^,^^11^ All the identified fracture loci were also associated with BMD, supporting the notion that BMD is a shared major risk factor for fractures at different bone sites. Nevertheless, it is likely that the causal mechanisms at least partly differ by fracture location. For instance, vertebral fracture risk depends largely on trabecular BMD, while risk of falls is an important determinant of hip fracture risk.^13^

We, herein, hypothesized that bone-site specific fracture loci, also including non-BMD-related loci, exist for hip fractures. To evaluate this hypothesis, we established a large dataset of hip fracture cases using data from five large Northern European biobanks in which high-quality national registers had been used to identify hip fractures according to the International Classification of Diseases (ICD; ICD10 codes S72.0–S72.2 and ICD9 820) and performed a large-scale hip fracture GWAS meta-analysis, including age- and gender-stratified analyses. This dataset was also used in an MR setting to identify causal clinical risk factors for hip fractures.

## Results

### Genetic loci associated with hip fractures

We included 11,516 hip fracture cases and 723,838 controls from the HUNT, UFO, UK Biobank, Estonian Biobank, and FinnGen biobanks. The associations for 9,457,767 variants (minor allele frequency [MAF] > 0.01, imputation quality > 0.3) with hip fractures were evaluated. No evidence of excessive genomic inflation (λ = 1.02, linkage disequilibrium [LD] score intercept = 0.999) was observed in the GWAS meta-analysis, suggesting that the results were not biased because of population stratification, genotyping artifacts, or cryptic family relationships (Figure S1).

Five genomic loci were at a genome-wide significant level associated with hip fracture risk (Tables 1 and S5; Figures S1–S3). One of these hip fracture signals (rs429358 at the *APOE* locus on chromosome 19q13.32, p = 3.8 × 10^−11^) is identical to the main established amino acid-altering genetic signal for Alzheimer’s disease (Table 1).^14^ The C allele of this SNP, which is associated with increased risk of Alzheimer’s disease (Table 1; Figure S3A), was associated with increased risk of hip fractures (odds ratio [OR] per C allele, 1.14; 95% confidence interval [CI] 1.10–1.19) and increased risk of falls (Table 1). In contrast, it was not significantly associated with the combined less-validated fracture trait fractures at any bone site or with any measures of BMD (Table 1). Age-stratified analyses revealed that this genetic signal was associated with hip fracture risk in old (≥71.2 years, OR per C allele, 1.33; 95% CI 1.25–1.43) but not in young (<71.2 years, OR 1.03; 95% CI 0.97–1.10) subjects, while gender-stratified analyses revealed similar associations for men and women (Table 1).

**Table 1:** Associations for BMD-related and non-BMD-related genome-wide significant SNPs for hip fractures

	Non-BMD-related locus			BMD-related loci												n	
	*APOE (19q13.32)*			*ETS2 (21q22.2)*			*SALL1 (16q12.1)*			*REST (4q12)*			*HOXC8 (12q13.13)*				
	rs429358, (EA C, EAF 0.17)			rs11088458 (EA G, EAF 0.70)			rs62028332 (EA G, EAF 0.87)			rs35339719 (EA G, EAF 0.74)			rs4142680 (EA T, EAF 0.41)^a^				
Fractures	OR	95% CI	p	OR	95% CI	p	OR	95% CI	p	OR	95% CI	p	OR	95% CI	p	Cases	Controls

Hip fracture	1.14	(1.10–1.19)	3.8 × 10^−11^	1.11	(1.07–1.14)	3.7 × 10^−10^	1.15	(1.10–1.20)	1.4 × 10^−9^	1.11	(1.07–1.14)	1.8 × 10^−9^	1.12	(1.08–1.16)	2.2 × 10^−9^	11,516	723,838
Old	1.33	(1.25–1.43)	3.1 × 10^−17^	1.11	(1.05–1.18)	1.3 × 10^−4^	1.18	(1.10–1.26)	6.5 × 10^−6^	1.08	(1.02–1.14)	7.2 × 10^−3^	1.09	(1.03–1.14)	1.8 × 10^−3^	4,700	609,945
Young	1.03	(0.97–1.10)	3.0 × 10^−1^	1.10	(1.05–1.16)	1.9 × 10^−4^	1.15	(1.07–1.23)	5.6 × 10^−5^	1.15	(1.09–1.21)	9.8 × 10^−8^	1.14	(1.09–1.20)	1.6 × 10^−7^	4,334	609,945
Men	1.14	(1.06–1.23)	3.9 × 10^−4^	1.09	(1.03–1.16)	5.8 × 10^−3^	1.08	(1.00–1.17)	5.8 × 10^−2^	1.11	(1.04–1.17)	1.3 × 10^−3^	1.12	(1.05–1.18)	2.5 × 10^−4^	3,070	266,328
Women	1.17	(1.11–1.24)	3.8 × 10^−8^	1.12	(1.07–1.17)	3.3 × 10^−6^	1.21	(1.14–1.28)	1.6 × 10^−9^	1.11	(1.06–1.17)	4.0 × 10^−6^	1.12	(1.07–1.17)	7.6 × 10^−7^	5,980	343,617
Fracture at any bone site	1.01	(0.99–1.02)	6.0 × 10^−1^	1.05	(1.03–1.06)	4.5 × 10^−11^	1.04	(1.03–1.06)	6.9 × 10^−6^	1.02	(1.00–1.03)	8.9 × 10^−3^	1.01	(0.99–1.02)	2.4 × 10^−1^	53,184	373,611

Other binary outcomes																	

Falls	1.02	(1.01–1.03)	6.3 × 10^−4^	1.01	(1.00–1.02)	8.5 × 10^−2^	1.00	(0.98–1.01)	5.2 × 10^−1^	1.00	(0.99–1.01)	8.3 × 10^−1^	1.01	(1.00–1.02)	1.0 × 10^−1^	89,076	362,103
Alzheimer′s disease	3.33	(3.20–3.45)	1.2 × 10^−881^	0.98	(0.95–1.01)	2.3 × 10^−1^	0.99	(0.94–1.03)	5.4 × 10^−1^	1.00	(0.97–1.03)	8.3 × 10^−1^	1.02	(0.98–1.05)	3.3 × 10^−1^	35,274	59,163

BMD-related traits	Beta	SE	p	Beta	SE	p	Beta	SE	p	Beta	SE	p	Beta	SE	p	n	

FN-BMD	−0.010	0.011	4.0 × 10^−1^	−0.030	0.008	4.1 × 10^−4^	−0.060	0.012	3.0 × 10^−7^	0.007	0.008	4.1 × 10^−1^	−0.04	0.012	9.8 × 10^−4^	49,998	
LS-BMD	0.006	0.013	6.3 × 10^−1^	−0.025	0.010	1.1 × 10^−2^	−0.023	0.013	9.0 × 10^−2^	0.016	0.010	1.1 × 10^−1^	−0.057	0.012	6.0 × 10^−6^	44,731	
eBMD	0.001	0.003	7.9 × 10^−1^	−0.044	0.002	6.8 × 10^−82^	−0.041	0.004	5.2 × 10^−20^	−0.015	0.002	1.8 × 10^−11^	−0.002	0.003	6.3 × 10^−1^^b^	426,824	

Besides the data for the five identified genetic signals in the present hip fracture GWAS meta-analyses, look ups were performed in the following published GWASs: fracture at any bone site,^11^ falls,^15^ Alzheimer′s disease,^14^ eBMD,^11^ and FN-BMD and LS-BMD.^12^ Odds ratios (OR) for binary outcomes are given per effect allele. Betas for continuous BMD-related parameters are expressed as SD per effect allele. EA, effect allele; EAF, effect allele frequency; BMD, bone mineral density; FN, femoral neck; LS, lumbar spine; eBMD, estimated bone mineral density in the heel using ultrasound. To achieve effect estimates not confounded by a possible minor dilution by diaphyseal and distal femur fractures and lack of adjustment for height and weight in the publicly available analyses of the FinnGen cohort, we replicated the five genome wide signals for hip fracture in a meta-analyses excluding the FinnGen cohort, yielding similar effect estimates (meta-analyses results excluding FinnGen; APOE, rs429358 OR 1.16 95% CI 1.11–1.21, p = 2.5 × 10^−10^; *ETS2*, rs11088458 OR 1.11 95% CI 1.07–1.15, p = 7.4 × 10^−8^; in the *ETS2* locus, the correlated SNP rs8130983 [r^2^ = 0.73] was genome-wide significant in analyses without FinnGen OR 1.12 95% CI 1.08–1.16, p = 1.1 × 10^−8^; SALL1, rs62028332 OR 1.16 95% CI 1.10–1.22, p = 3.1 × 10^−9^; *REST*, rs35339719 OR 1.11 95% CI 1.07–1.15, p = 3.4 × 10^−8^; *HOXC8*, rs4142680 OR 1.12 95% CI 1.08–1.16, p = 2.2 × 10^−9^).

a Information for rs4142680 and hip fractures was only available in HUNT, UK Biobank, and UFO (8,401 cases and 501,168 controls).

b Derived from Neale et al. (http://www.nealelab.is/uk-biobank/), n = 206,496 as this SNP was not present in Zheng et al.^12^

The other four genomic loci associated with hip fractures (*REST* at chromosome 4q12, *HOXC8* at chromosome 12q13.13, *SALL1* at chromosome 16q12.1, and *ETS2* at chromosome 21q22.2; Figures S3B–S3E), were at, or near, loci previously shown to be associated with different BMD measures (Table 1).^10^^,^^11^ Gender-stratified analyses of these BMD-related loci revealed that the association for the genetic signal at the *SALL1* locus was more pronounced in women than in men (p = 3.2 × 10^−2^ for differences between ORs, z test; Table 1). The alleles of the four BMD-related genetic signals that associated with increased hip fracture risk were all associated with decreased femoral neck BMD (*ETS2*, *SALL1*, and *HOXC8*) and/or decreased estimated BMD by ultrasound in the heel (*ETS2*, *SALL1*, and *REST*) but not with risk of falls or Alzheimer’s disease (Table 1).

### Genetic correlation with clinical risk factors

LD score regression (LDSR) was used to estimate the genetic correlation between hip fractures and different diseases and traits. We evaluated in total 17 different genetic correlations for fractures at any bone site, BMD measures, and clinical risk factors for hip fractures (Table 2).

**Table 2:** Estimated genetic correlation between hip fractures and risk factors for hip fractures

Disease or trait	Genetic correlation		
	r_g_	SE	p
Fracture at any bone site	0.68^a^	0.10^a^	4.6 × 10^−11^^a^

BMD-related measures			

FN-BMD^b^	−0.89^a^	0.10^a^	5.9 × 10^−20^^a^
LS-BMD^b^	−0.43^a^	0.09^a^	1.2 × 10^−6^^a^
eBMD	−0.48^a^	0.06^a^	5.7 × 10^−16^^a^

Clinical risk factors			

Age at menopause^b^	−0.06	0.07	4.3 × 10^−1^
Only females^b^	−0.01	0.11	9.1 × 10^−1^
Age at menarche^b^	−0.01	0.05	8.1 × 10^−1^
Only females^b^	−0.05	0.07	4.7 × 10^−1^
Relative age voice broke^b^	0.01	0.07	9.0 × 10^−1^
Only males^b^	−0.20	0.13	1.3 × 10^−1^
Grip strength	−0.12	0.05	7.8 × 10^−3^
Vitamin D levels	−0.01	0.06	8.9 × 10^−1^
Falls	0.35^b^	0.07^b^	2.1 × 10^−7^^a^
Coronary artery disease^b^	−0.04	0.06	5.4 × 10^−1^
Rheumatoid arthritis^b^	0.06	0.07	4.4 × 10^−1^
Inflammatory bowel disease^b^	0.06	0.07	3.8 × 10^−1^
Type 2 diabetes^b^	−0.07	0.10	4.5 × 10^−1^
Ever vs never smoked^b^	0.23	0.08	3.9 × 10^−3^
Alcohol consumption	0.13	0.05	6.9 × 10^−3^
Alzheimer′s disease	0.08	0.11	4.7 × 10^−1^

We evaluated the genetic correlation for plausible risk factors and Alzheimer′s disease with hip fractures. The genetic correlations were evaluated either using LDhub or locally using public available GWAS summary statistics (fractures at any bone site,^7^ eBMD,^11^ grip strength,^16^ falls,^15^ vitamin D,^17^ alcohol consumption,^18^ Alzheimer′s disease^14^). For diseases/traits including UK-Biobank in the GWAS and displaying significant genetic correlations (p < 0.05/17 = 0.0029) with hip fractures, sensitivity analyses were performed excluding UK Biobank in the hip fracture meta-analysis used for the correlations (analyses excluding UK Biobank: eBMD r_g_ = −0.53, p = 5.1 × 10^−10^; falls r_g_ = 0.33, p = 2 × 10^−4^). BMD, bone mineral density; FN, femoral neck; LS, lumbar spine; eBMD, estimated bone mineral density in the heel using ultrasound.

a Significant genetic correlation with risk of hip fracture passing Bonferroni adjusted level (p<0.05/17= 0.0029).

b Genetic correlations evaluated in the LD hub.

Femoral neck BMD (FN-BMD) was strongly (*r*_g_ = −0.89), while lumbar spine BMD (LS-BMD; *r*_g_ = −0.43) and estimated BMD in the heel using ultrasound (eBMD; *r*_g_ = −0.48) were moderately, inversely genetically correlated with hip fractures (Table 2). Among the different other evaluated clinical risk factors, only falls passed Bonferroni correction (p < 0.05/17 = 0.0029) and were directly genetically correlated with hip fractures (*r*_g_ = 0.35, p = 2.1 × 10^−7^). In addition, hand grip strength was indirectly, while ever versus never smoked and alcohol consumption were directly, nominally (p < 0.05) genetically correlated with hip fractures, but these correlations did not pass Bonferroni correction for multiple testing (Table 2). None of the other evaluated risk factors was significantly genetically correlated with hip fractures (Table 2).

### MR

**Figure 1:**
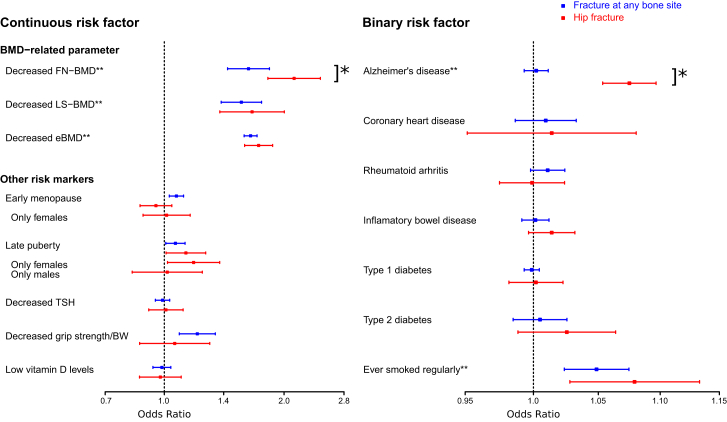
Mendelian randomization to estimate the causal effects of 15 genetically determined risk factors on risks of hip fractures (red) and fractures at any bone site (blue) Estimates for the association with hip fracture is from the present hip fracture GWAS, while the estimates from any fracture are from the summary statistics in Morris et al.^11^ Inverse variance weighted meta-analyses were performed. Odds ratio (OR) and 95% confidence intervals are for the risk of fractures per standard deviation change in the risk factor for continuous trait or risk of fracture per doubling of odds (obtained by multiplying the causal estimate of log odds by ln(2) ≈ 0.69)^19^ of disease susceptibility for binary factors. For ever smoked regularly, the ORs are expressed per 0.5 unit increase in log odds of ever smoking regularly with a 1 standard deviation increase in genetically predicted smoking initiation corresponding to a 10% increased risk of smoking.^18^^,^^22^ Estimates are displayed using a random effects model to account for possible heterogeneity. ∗, the ORs differ significantly between hip fracture and fractures at any bone site as determined by a z test. ∗∗, risk factors that remain significantly causally associated with hip fractures after correction for multiple testing ( p < 0.05/15 = 3.3 × 10^−3^). For risk factors including UK Biobank in the GWAS and displaying significant causal effects with hip fractures, sensitivity analyses were performed excluding UK Biobank in the hip fracture meta-analysis used for the Mendelian randomization, revealing essentially similar effect estimates (results excluding UK Biobank in the hip GWAS; decreased eBMD OR = 1.66; 95% confidence interval [CI]: 1.54–1.79; ever smoked regularly OR = 1.07; 95% CI: 1.01–1.14). Grip strength is given as grip strength per body weight. Standard deviation (SD) for grip strength is given for kg grip strength per kg in body weight and was estimated in the UK Biobank to be 0.127. To achieve effect estimates not confounded by a possible minor dilution by diaphyseal and distal femur fractures among hip fractures and lack of adjustment for height and weight in the publicly available analysis of the FinnGen cohort, we replicated the significant causal associations for FN-BMD (OR 2.25; 95% CI 1.91–2.65), Alzheimer’s disease (OR 1.08; 95% CI 1.06–1.11), and ever smoked regularly (OR 1.06; 95% CI 1.00–1.12) in a meta-analysis excluding the FinnGen cohort, yielding similar effect estimates. BMD, bone mineral density; TSH, thyroid-stimulating hormone; FN, femoral neck; LS, lumbar spine; eBMD, estimated BMD in the heel using ultrasound.

**Figure 2:**
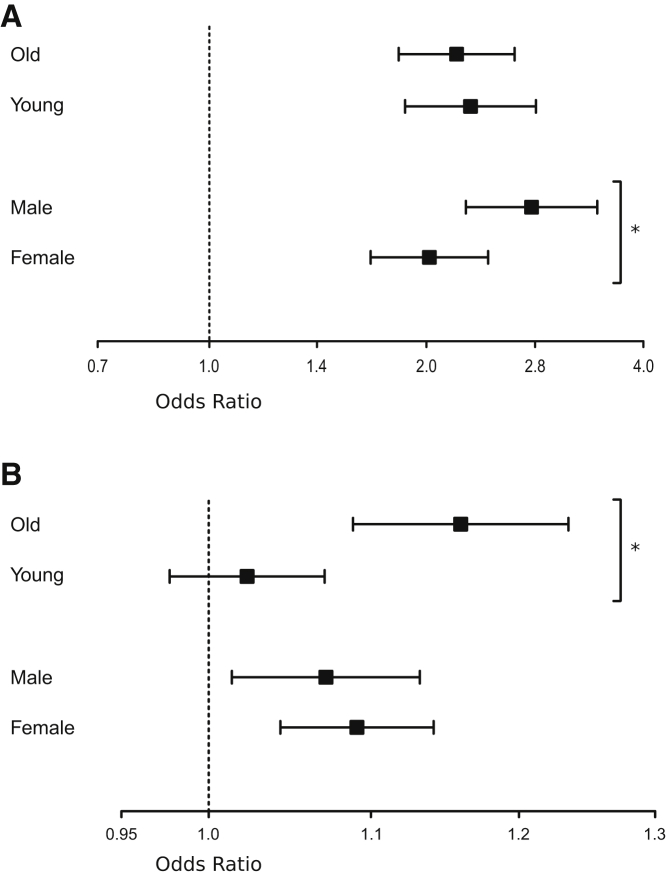
Age- and gender- stratified Mendelian randomization analyses Age- and gender-stratified Mendelian randomization to estimate the causal effects of (A) decreased FN-BMD and (B) Alzheimer’s disease on hip fracture risk. Inverse variance weighted meta-analysis were performed. OR and 95% CIs are for the risk of hip fractures per SD decrease in genetically determined FN-BMD or per doubling of odds of Alzheimer’s disease. Estimates are displayed using a random effects model to account for possible heterogeneity. Age-stratified analyses are divided by the median age (71.2 years) of the hip fracture cases. ∗, the ORs differ as determined by a z test (p < 0.05).

Two-sample MR was used to test the causal effect of 15 plausible risk factors on hip fractures (Tables 3 and S7; Figure 1), and these effects were also compared with the effects on fractures at any bone site. There was clear evidence of a strong causal effect of genetically decreased FN-BMD (OR per SD decrease 2.12; 95% CI 1.82–2.47, p = 3.7 × 10^−22^) and a moderate causal effect of genetically decreased LS-BMD (OR per SD decrease 1.66; 95% CI 1.38–2.00, p = 9.9 × 10^−8^) and eBMD (OR per SD decrease 1.73; 95% CI 1.59–1.87, p = 1.1 × 10^−39^; Figure 1; Table 3) on hip fracture risk. Compared with the causal effect on fractures at any bone site, the causal effect on hip fractures was significantly more pronounced for genetically decreased FN-BMD (p = 6.0 × 10^−3^ using z test) but not for genetically decreased LS-BMD or eBMD (Figure 1; Table 3). Stratified analyses revealed that the magnitude of the causal effect of genetically decreased FN-BMD on hip fracture risk was similar in young (<71.2 years) and old (≥71.2 years) subjects, while it was slightly more pronounced in men (OR per SD decrease 2.80; 95% CI 2.27–3.45, p = 7.2 × 10^−22^) compared with women (OR per SD decrease 2.02; 95% CI 1.67–2.44, p = 2.1 × 10^−13^; p = 2.3 × 10^−2^ men versus women using z test; Figure 2A). When the individual genome-wide significant top SNPs in the different loci from an FN-BMD GWAS^10^ were evaluated in a candidate approach, we observed that the lead SNP in as many as 25 of 47 loci was significantly (p < 0.05) associated with hip fracture risk in the expected direction (the allele associated with increased FN-BMD was associated with reduced hip fracture risk; Table S8). These data support the MR finding of a strong causal role of low FN-BMD on increased hip fracture risk.

**Table 3:** Mendelian randomization to estimate the causal effects of 15 genetically determined risk factors on hip fractures

Trait or disease	Inverse variance weighted meta-analyses									Egger regression	
	Hip fractures			Any fractures			Power			Intercept, p	
							Hip		Any	Hip fractures	Any fractures
	OR	95% CI	p	OR	95% CI	p	OR 1.15, %	OR 1.20, %	OR 1.15, %		
Continuous risk factors											

BMD-related parameters											
Decreased FN-BMD	2.12	(1.82–2.47)	3.7 × 10^−22^^a^	1.63	(1.44–1.84)	3.3 × 10^−15^^a^	96	100	100	1.2 × 10^−1^	4.4 × 10^−1^
Decreased LS-BMD	1.66	(1.38–2.00)	9.9 × 10^−8^^a^	1.56	(1.39–1.76)	7.2 × 10^−14^^a^	97	100	100	5.5 × 10^−4^^a^	4.1 × 10^−1^
Decreased eBMD	1.73	(1.59–1.87)	1.1 × 10^−39^^a^	1.65	(1.59–1.71)	3.0 × 10^−151^^a^	100	100	100	6.4 × 10^−2^	8.5 × 10^−1^

Other risk markers											

Early menopause	0.95	(0.87–1.04)	3.0 × 10^−1^	1.07	(1.03–1.12)	9.0 × 10^−4^^a^	96	100	100	2.3 × 10^−1^	8.8 × 10^−1^
Only females	1.01	(0.88–1.16)	8.5 × 10^−1^	N/A	N/A	N/A	75	95	N/A	9.6 × 10^−2^	N/A
Late puberty	1.13	(1.01–1.27)	3.3 × 10^−2^	1.07	(1.01–1.13)	2.5 × 10^−2^	69	91	99	3.2 × 10^−2^	8.5 × 10^−1^
Only females	1.18	(1.02–1.38)	2.8 × 10^−2^	N/A	N/A	N/A	40	65	N/A	2.5 × 10^−1^	N/A
Only males	1.02	(0.83–1.24)	8.7 × 10^−1^	N/A	N/A	N/A	22	37	N/A	4.1 × 10^−3^	N/A
Decreased TSH	1.01	(0.91–1.11)	8.6 × 10^−1^	0.99	(0.95–1.03)	6.2 × 10^−1^	95	100	100	4.8 × 10^−1^	8.1 × 10^−1^
Decreased grip strength/BW	1.06	(0.87–1.30)	5.6 × 10^−1^	1.21	(1.09–1.34)	3.4 × 10^−4^^a^	48	74	91	3.8 × 10^−1^	2.4 × 10^−1^
Low vitamin D levels	0.98	(0.87–1.10)	7.1 × 10^−1^	0.99	(0.94–1.04)	5.9 × 10^−1^	99	100	100	3.8 × 10^−1^	1.8 × 10^−1^

Binary risk factors											

Alzheimer′s disease	1.07	(1.05–1.10)	1.9 × 10^−12^^a^	1.00	(0.99–1.01)	6.5 × 10^−1^	100	100	100	7.2 × 10^−1^	7.6 × 10^−1^
Coronary heart disease	1.01	(0.95–1.08)	6.7 × 10^−1^	1.01	(0.99–1.03)	4.3 × 10^−1^	95	100	100	4.0 × 10^−1^	2.6 × 10^−1^
Rheumatoid arthritis	1.00	(0.97–1.02)	9.3 × 10^−1^	1.01	(1.00–1.02)	1.0 × 10^−1^	95	100	100	6.3 × 10^−1^	4.3 × 10^−1^
Inflammatory bowel disease	1.01	(1.00–1.03)	1.2 × 10^−1^	1.00	(0.99–1.01)	7.8 × 10^−1^	99	100	100	3.6 × 10^−1^	8.9 × 10^−1^
Type 1 diabetes	1.00	(0.98–1.02)	8.6 × 10^−1^	1.00	(0.99–1.00)	6.6 × 10^−1^	98	100	100	5.5 × 10^−1^	8.4 × 10^−1^
Type 2 diabetes	1.03	(0.99–1.06)	1.8 × 10^−1^	1.00	(0.98–1.03)	6.3 × 10^−1^	95	100	100	2.2 × 10^−1^	7.6 × 10^−2^
Ever smoked regularly	1.08	(1.03–1.13)	2.3 × 10^−3^^a^	1.05	(1.03–1.07)	1.4 × 10^−4^^a^	61	96	97	7.6 × 10^−1^	2.3 × 10^−1^

Inverse variance weighted meta-analysis. Estimates for the association with hip fracture are from the present hip fracture GWAS, while the estimates from fractures at any bone site are from the summary statistics in Morris et al.^11^ Odds ratio (OR) is for the risk of fracture per standard deviation (SD) change in the risk factor for continuous trait or risk of fracture per doubling of odds (obtained by multiplying the causal estimate of log odds by ln(2) ≈ 0.69)^19^ of disease susceptibility for binary factors. For menopause and puberty, we used the estimated SD from the largest cohorts in the published GWAS (early menopause SD = 3.81 years in Breast Cancer Association Consortium;^20^ late puberty SD = 1.40 years in Women’s Genome Health Study^21^) to translate the effect unit from year to SD. For ever smoked regularly, the ORs are expressed per 0.5 unit increase in log odds of ever smoking regularly with a 1 SD increase in genetically predicted smoking initiation corresponding to a 10% increased risk of smoking.^18^^,^^22^ Estimates are displayed using a random effects model to account for possible heterogeneity. Statistical power is given to detect an odds ratio of 1.15 or 1.20 at α ≤ 3.3 × 10^−3^ (0.05/15 risk factors). Egger intercepts are given in this table, while Egger effect estimates are presented in Table S7.For risk factors including UK Biobank in the GWAS and displaying significant causal effects with hip fractures, sensitivity analyses were performed excluding UK Biobank in the hip fracture meta-analysis used for the mendelian randomization, revealing essentially similar effect estimates (results excluding UK Biobank in the hip fracture GWAS; decreased eBMD OR = 1.66, 95% CI: 1.54–1.79; ever smoked regularly OR = 1.07, 95% CI: 1.01–1.14). Grip strength is given as grip strength per body weight (BW). SD for grip strength is given for kg grip strength per kg in BW and was estimated in the UK Biobank to be 0.127. To achieve effect estimates not confounded by a possible minor dilution by diaphyseal and distal femur fractures and lack of adjustment for height and weight in the publicly available analyses of the FinnGen cohort, we replicated the significant causal associations for FN-BMD (OR 2.25, 95% CI 1.91–2.65), Alzheimer′s disease (OR 1.08, 95% CI 1.06–1.11), and ever smoked regularly (OR 1.06, 95% CI 1.00–1.12) in a meta-analysis excluding the FinnGen cohort, yielding similar effect estimates. N/A, not available; BMD, bone mineral density; TSH, thyroid-stimulating hormone; FN, femoral neck; LS, lumbar spine; eBMD, estimated BMD in the heel using ultrasound; CI, confidence interval.

a Findings that remain significantly associated after correction for multiple testing (p < 0.05/15 = 3.3 × 10^−3^).

In addition, genetically determined Alzheimer’s disease was causally associated with increased risk of hip fractures (Figure 1; Table 3), and a sensitivity study excluding the genome-wide significant hip fracture SNP rs429358 at the *APOE* locus revealed a similar causal association (Figure S4). When evaluating the individual genome-wide significant top SNPs in the different loci from the used Alzheimer’s disease GWAS,^14^ we observed that, besides the top SNP in the *APOE* locus, the top SNP in the *CD2AP* locus on chromosome 6 was significantly associated with risk of hip fractures (Table S9). In addition, rs7412 in the *APOE* locus was modestly associated with hip fractures (OR 1.06, 95% CI 1.01–1.12 per C allele, p = 0.03). Stratified analyses demonstrated that the causal effect of genetically determined Alzheimer’s disease on hip fractures was robust in old (≥71.2 years), but not in young (<71.2 years), subjects (Figure 2B). The causal effect of genetically determined Alzheimer’s disease was of similar magnitude in men and women (Figure 2B).

Ever smoked regularly was also causally associated with increased risk of hip fractures and fractures at any bone site (Figure 1; Table 3). MR demonstrated that this smoking parameter was causally related to risk of falls (summary statistics for falls from Trajanoska et al.;^15^ OR: 1.08, 95% CI: 1.06–1.10, p = 7.7 × 10^−16^) but not FN-BMD (summary statistics for FN-BMD from Zheng et al;.^12^ beta = −0.009, SE = 0.014; p = 0.51).

Some evidence was observed that late puberty was causally associated with increased hip fracture risk in females, but this association did not pass Bonferroni correction. None of the other 9 evaluated risk factors for fractures showed evidence of a causal effect on risk of hip fractures, despite often adequate statistical power (Figure 1; Tables 3 and S6). For all of the reported significant causal associations, the estimates from the inverse variance weighted fixed effects meta-analysis were very similar to the estimates from the weighted median and penalized weighted median methods (Table S7).

To exclude reverse causality, wherein hip fractures influence risk of Alzheimer’s disease, we performed a bidirectional MR using hip fracture (GWAS SNPs from the present hip fracture GWAS, excluding the established strong Alzheimer’s SNP rs429358) as the exposure and risk of Alzheimer’s disease^14^ as the outcome, revealing no evidence that genetically determined hip fracture was causally related to Alzheimer’s disease (OR per doubling of diseases susceptibility 0.96; 95% CI 0.82–1.12, p = 0.63).

As hip fracture risk is associated with increased risk of falls and reduced FN-BMD in observational studies, we determined the effects of genetically determined Alzheimer’s disease on risk of falls and FN-BMD. We observed that genetically determined Alzheimer’s disease (GWAS SNPs from Kunkle at al.^14^) was causally related to increased risk of falls (GWAS summary statistics from Trajanoska et al.;^15^ OR per doubling of diseases susceptibility 1.02; 95% CI 1.01–1.03; p = 6.3 × 10^−3^) but not to FN-BMD (summary statistics from Zheng et al.;^12^ SD per doubling of diseases susceptibility beta = 0.015, SE = 0.012; p = 0.21). Therefore, it is biologically plausible that Alzheimer’s disease increases hip fracture risk at least partly via increased risk of falls without altering FN-BMD. A multivariable analysis using both Alzheimer’s disease and falls as exposures did not support a strong mediating effect of falls for the causal association between Alzheimer’s disease and hip fractures (Table S10). However, it should be noted that the genome-wide significant SNPs derived from the falls GWAS explained only 0.28% of the variation in falls,^15^ suggesting limited power in this multivariable analysis. In addition, as Alzheimer’s disease results in reduced memory, it is possible that patients with Alzheimer’s disease underreport falls.

The three MR assumptions were evaluated as previously described.^7^ We only selected variants with a MAF > 1% that were strongly associated with the clinical risk factor (p < 5 × 10^−8^), ensuring that the genetic variants used as instrumental variables are associated with the clinical risk factor (first assumption). The impact of reported associations between the genetic variants and potential confounding factors (second assumption) was searched for in the literature and in the GWAS catalog (https://www.ebi.ac.uk/gwas/). We observed that the genetic signal for both hip fractures (the present study) and Alzheimer’s disease, rs429358, was also robustly associated with C-reactive protein levels and serum cholesterol levels. However, using MR and adequate genetic instruments (Table S11), we did not observe any evidence of a causal effect of C-reactive protein (CRP; genetic instruments from Han et al.^23^) or low-density lipoprotein (LDL) cholesterol or high-density lipoprotein (HDL) cholesterol (genetic instruments for both HDL and LDL cholesterol from Willer et al.^24^) on hip fractures (inverse variance weighted MR; CRP, OR 0.95 (0.88–1.02), p = 0.14; LDL cholesterol OR 1.01 (0.92–1.12), p = 0.80; HDL cholesterol OR 1.03 (0.93–1.14) p = 0.56; ORs are expressed per SD increase in exposure). Finally, for the reported significant causal associations, no evidence of horizontal pleiotropy between the instruments and the outcomes was observed, except for LS-BMD, using MR Egger regression (Tables 3 and S7).

## Discussion

Although hip fracture is the clinically most important fracture, the genetic architecture and the causal risk factors of hip fractures are not characterized in detail. We, herein, established a large hip fracture dataset and performed a large-scale GWAS meta-analysis on hip fractures, identifying one non-BMD-related genetic hip fracture signal and four signals in loci known to be involved in bone biology. In addition, we demonstrate that the causal clinical risk factors for hip fractures include decreased FN-BMD, Alzheimer’s disease, and ever smoked regularly. We also demonstrate that the impact of these causal risk factors differs by age, gender, and fracture site. Finally, we provide causal effect estimates of the effect of FN-BMD on hip fractures, including separate effect estimates by age and gender, supporting the use of change in FN-BMD as an efficient surrogate outcome to estimate effects of BMD-targeting treatments on hip fractures in future clinical trials.

Interestingly, in the present GWAS on hip fracture, one of the identified genetic signals, located in the *APOE* locus, is associated with falls but not associated with any BMD measures. Falls are a major determinant of hip fractures,^2^, ^3^, ^4^^,^^13^ and the allele of the *APOE* signal associated with increased hip fracture risk was also associated with increased risk of falls, suggesting that this signal has an effect on hip fracture risk via risk of falls and not via bone strength. In addition, it is possible that this signal also may influence the pattern of falls as decline in age-related neuromuscular function is reported to change how we fall. When not appropriately stretching out the arms, we more often fall on the side, directly on the hip.^25^ Age-stratified analyses revealed that this genetic signal was associated with hip fracture risk in old, but not in young, subjects. The hip fracture signal identified at the *APOE* locus is identical to the main established amino acid-altering genetic signal for Alzheimer’s disease (in exon 4 of Apo lipoprotein E).^14^^,^^26^ The allele that increases the risk of Alzheimer’s disease also increases the risk of hip fractures and falls.

Our MR analyses demonstrated that Alzheimer’s disease is causally associated with increased hip fracture risk, and a sensitivity study excluding the genome-wide significant hip fracture signal at the *APOE* locus revealed a similar causal association. In support of a mechanism of Alzheimer’s disease on hip fracture risk involving increased risk of falls, MR demonstrated that Alzheimer’s disease was causally directly related to falls but not to FN-BMD. Furthermore, genetic correlation analyses revealed that falls were significantly directly correlated with hip fractures, suggesting that the genetic architecture of falls and hip fractures are partly overlapping. Previous observational studies have reported that Alzheimer’s disease is associated with increased number and severity of falls.^13^^,^^26^

The signals in the other four genomic loci associated with hip fractures (*REST*, *HOXC8*, *SALL1*, and *ETS2*) were previously shown to associate with different BMD measures,^10^^,^^11^ supporting the notion that BMD is an important determinant also of hip fracture risk. None of these four BMD-related signals were associated with risk of falls, suggesting that these signals mainly affected hip fracture risk via reduced bone strength. The signal in the *ETS2* locus has previously been identified in a GWAS on fractures at any bone site, while the signals at the *SALL1* and *REST* loci were nominally (p < 0.05) significantly associated with fractures at any bone site in the expected direction.^7^^,^^11^ A highly correlated SNP to the top SNP in the *HOXC8* locus was identified in a GWAS on LS bone area to also be associated with fractures.^27^ Thus, all these four identified BMD-related hip fracture signals are known to be involved in bone biology.

A validated surrogate outcome for fracture would reduce the size, duration, and cost of trials of new osteoporosis treatments, thereby facilitating drug development. Thus, there is a clinical need of an FDA-approved surrogate marker for fractures as outcomes in clinical osteoporosis trials.^8^ Two-sample MR, using strong genetic instruments, is a methodology to obtain precise causal effect estimates of FN-BMD on hip fractures that is not affected by confounders or reverse causality. Using the present large-scale GWAS on hip fractures, we demonstrated a strong causal association of decreased FN-BMD on increased hip fracture risk. Our further stratified analyses yielded separate effect estimates by age and gender, revealing that FN-BMD exerts a substantial causal effect on hip fracture risk in both men and women and in both young and old subjects, supporting the use of change in FN-BMD as a general surrogate outcome in randomized clinical trials that estimate the effects of BMD-targeting treatments on hip fractures.

In addition to decreased FN-BMD and Alzheimer’s disease, ever smoked regularly was causally associated with increased risk of hip fracture. Ever smoked regularly was chosen as a measure of smoking, as strong genetic instruments were available for this trait.^18^ As there was some overlap between the populations included in the GWAS for ever smoked regularly and the present GWAS on hip fractures, this causal association should be confirmed in additional cohorts. Interestingly, this smoking parameter was directly causally associated with risk of falls but not associated with FN-BMD. In addition to increasing risk of falls, it is possible that smoking may regulate bone strength via effects on bone microstructure or other bone quality parameters, which are not captured by FN-BMD. A causal effect of smoking on hip fracture risk is supported by some previous observational association studies.^28^^,^^29^

The strength of this study is the large number of hip fracture cases included, generating the most comprehensive assessment of the genetic determinants of hip fracture risk so far. This well-powered dataset also enabled us to identify and estimate the strength of causal clinical risk factors for hip fractures.

In conclusion, this hip fracture GWAS identified one non-BMD-related and four BMD-related genetic determinants for hip fractures. MR analyses demonstrated a strong causal effect of decreased FN-BMD and moderate causal effects of Alzheimer’s disease and ever smoked regularly on risk of hip fractures. The present study demonstrates that the genetic architecture of fractures is complex and at least partly differs by bone site, age, and gender and, for hip fractures, involves both BMD-related and non-BMD-related signals. The substantial causal effect of FN-BMD on hip fracture risk in both young and old subjects and in both men and women supports the use of change in FN-BMD as a general surrogate outcome in randomized clinical trials to estimate effects of BMD-targeting treatments on hip fractures.

### Limitations of the study

Some limitations of the present study need to be considered. As the available genetic instruments for falls and alcohol consumption were very weak, the causal associations for these two relevant risk factors for hip fractures could not be evaluated. Moreover, as the hip fracture GWAS was adjusted for height and weight, we could not assess the possible causal associations of BMI or height on hip fractures. In addition, it is a major limitation with the present study that the analyses were restricted to participants of White ancestry. Therefore, additional analyses are necessary to investigate whether our results also apply to those of other ethnicities. As MR assumes a linear relation between the risk factor and the outcome, the present finding of no apparent causal association between low vitamin D and hip fractures does not exclude a non-linear threshold association between low vitamin D and hip fractures. Also, the present findings need to be replicated in independent cohorts.

## Consortia

The Estonian Biobank Research Team includes Mari Nelis, Lili Milani, Tõnu Esko, and Andres Metspalu.

## STAR★Methods

### Key resources table

**Table:** 

REAGENT or RESOURCE	SOURCE	IDENTIFIER
**Deposited data**		

GWAS meta-analyses	This article	GWAS catalog, https://www.ebi.ac.uk/gwas/

**Software and algorithms**		

SAIGE	Zhou et al. 2018^30^	https://github.com/weizhouUMICH/SAIGE
PLINK	Chang et al. 2015^31^	https://www.cog-genomics.org/plink/1.9/
METAL	Willer et al. 2010^32^	https://genome.sph.umich.edu/wiki/METAL
LDSR	Bulik-Sullivan et al. 2015^33^	https://github.com/bulik/ldsc
R	The R Project for Statistical Computing	https://www.r-project.org/version 4.0.1

### Resource availability

#### Lead contact

Further information and requests for resources should be directed to and will be fulfilled by the lead contact Claes Ohlsson (claes.ohlsson@medic.gu.se).

#### Materials availability

This study did not generate unique reagents.

### Experimental model and subject details

#### Study population

We included subjects from five biobanks (*UFO* from Sweden; *HUNT* from Norway; *UK-Biobank* from UK; *Estonian Biobank* from Estonia and *FinnGen* from Finland) from some of the major Northern European biobanks with hip fracture data, relevant covariates, and genotype data available. To reduce potential bias due to population stratification, we restricted the analyses to studies with participants of European descent. In total 11,516 hip fracture cases and 723,838 controls were included. The study was approved by the local ethics review boards and study subjects provided written informed consent. For detailed information on the five contributing biobanks, please see supplemental methods (Tables S1–S4).

### Method details

#### Hip fracture definition

To specifically evaluate hip fractures, we only included hip fractures derived from high quality national registers based on medical and/or radiological reports and classified according to International Classification of Diseases (ICD; corresponding to ICD10 codes S72.0, S72.1 and S72.2 and ICD9 code 820; HUNT, UK Biobank, UFO, Estonian Biobank). Only hip fracture cases >30 years old were included. No self-reported hip fractures were included. Controls were defined as individuals from the same cohorts, without a history of hip fracture. For the FinnGen cohort, only public available summary statistics were available and for that analysis, a wider definition of hip fractures had been used corresponding to the ICD10 code S72, including not only the most common hip fractures (S72.0, S72.1 and S72.2) but also the less common diaphyseal and distal femur fractures. Predicted from the observed distribution in the large UK-Biobank, ∼90% of the femur fractures in the FinnGen would be hip fractures. To achieve effect estimates not confounded by a possible minor dilution by diaphyseal and distal femur fractures in the FinnGen cohort, we replicated the five genome-wide signals (Table 1) and the significant causal risk factors (Table 3) for hip fracture in a meta-analysis excluding the FinnGen cohort, yielding similar effect estimates.

### Quantification and statistical analysis

#### Genome-wide association study and meta-analyses

Genome-wide genotyping was performed in each cohort by use of Illumina or Affymetrix genome-wide genotyping chips and imputation was performed to ensure accurate ascertainment of nearly all common genetic variation above a minor allele frequency threshold of 1% (Table S4). We followed a standardized analytical plan to assess the association of single nucleotide polymorphisms (SNPs) with risk of hip fracture in each participating cohort. Logistic models using the SAIGE or PLINK software were used to estimate the SNP associations with hip fracture, adjusted for gender, age (simple and quadratic terms), height, weight, principal components, study site (when necessary) and family structure (if feasible), testing additive (per allele) genetic effects. Information on covariates for adjustments and study designs for the five included cohorts are presented in Table S1. When needed, individual GWAS summary results were corrected for population stratification by the genomic control inflation factor before we performed fixed effect inverse-variance weighted meta-analysis using METAL software. A total of 9,457,767 autosomal and X-linked SNPs present in more than two studies were meta-analysed. Allele and genotype frequencies of all genotyped variants followed Hardy-Weinberg equilibrium proportions. Further exploratory age- (divided by the median age of the hip fracture cases, 71.2 years) and gender-stratified sub-analyses were also performed.

#### Genetic correlation

To estimate the genetic correlation between hip fractures and other complex traits and diseases, we used (cross-trait) Linkage disequilibrium score regression (LDSR)^34^ as implemented in the online web utility LDHub.^35^ This method uses the cross-products of summary test statistics from two GWASs and regresses them against a measure of how much variation each SNP tags (its LD score).^36^ The LDSR analyses were restricted to HapMap3 SNPs with MAF >5% in the 1000 Genomes European reference population. We used pre-calculated LD scores from the same reference population (https://data.broadinstitute.org/alkesgroup/LDSCORE/).

In general, the selection of plausible clinical risk factors for evaluation of genetic correlation with hip fractures in Table 2 and for MR in Table 3 was similar as reported in a previous GWAS on fractures at any bone site*.*^7^ From the variety of traits available on LDHub,^33^ we selected 10 plausible risk factors for hip fractures.^15^ In addition, locally, we used the LDSCORE tool available from LDHub to estimate the genetic correlation between hip fracture risk and seven additional plausible risk factors for hip fractures using available GWAS summary statistics for fractures at any bone site,^7^ estimated BMD by ultrasound in the heel (eBMD),^11^ grip strength,^16^ vitamin D levels,^17^ falls,^15^ alcohol consumption,^18^ and Alzheimer′s disease.^14^ We accounted for multiple testing by using a conservative Bonferroni correction for 17 tests (p < 0.05/17 = 0.0029).

#### Mendelian randomization

To assess causal associations between plausible risk markers and hip fractures, we performed Two-sample mendelian randomization (MR) analyses and these associations were also compared with the effects on fractures at any bone site using summary statistics derived from a previous GWAS meta-analysis.^11^ We used genetic instrument variables obtained from selected GWAS as proxies for femoral neck BMD (FN-BMD),^10^ lumbar spine BMD (LS-BMD),^10^ eBMD,^11^ menopause^7^^,^^20^ puberty,^7^^,^^21^ thyroid-stimulating hormone (TSH),^7^^,^^37^ grip strength,^16^ vitamin D levels,^17^ Alzheimer’s disease,^14^ coronary heart disease,^7^^,^^38^ rheumatoid arthritis,^39^ inflammatory bowel disease,^7^^,^^40^ type 1 diabetes,^7^^,^^41^ type 2 diabetes,^7^^,^^42^ and ever smoked regularly.^18^ Although alcohol consumption and falls are plausible causal risk factors for hip fractures, these were not included in the MR analyses as the available genetic instruments were very weak, resulting in insufficient power in the analyses (Table S6).^15^^,^^18^ As genetic variants are randomly distributed at birth, they are unaffected by confounders. As the primary MR analyses, we used combined weighted estimates by an inverse-variance weighted (IVW) approach using fixed or random effects depending on Cochran’s Q statistic test of heterogeneity. We applied a conservative Bonferroni corrected threshold to account for the multiple testing (i.e., p < 0.05/15 = 0.0033, because 15 exposures were assessed). We then used the MR-Egger method as a sensitivity analysis to avoid possible uncontrolled pleiotropy. This method uses a weighted regression with an unconstrained intercept to regress the effect sizes of variant risk factor associations. It can thus detect some violations of the standard MR assumptions and provide an effect estimate, which is not subject to these violations.^43^ In further sensitivity analyses, we used the penalized weighted median MR method and the weighted median MR methods. The MR analyses were conducted with the R-package MendelianRandomization.^44^

R-Code for MR:

library("MendelianRandomization")

mrin < - mr_input(bx = …,bxse = …,by = …, byse = …) #bx & bxse are set to Beta and SE for the exposure (risk factor) and by & byse are set to Beta and SE for the outcome (fractures).

mrivwfix < - mr_ivw(mrin, model = "fixed")

mrivwrand < - mr_ivw(mrin, model = "random")

if(nrow(data)≥3){mrweightmed < - mr_median(mrin, weighting = "weighted")}

if(nrow(data)≥3){mrpenwightmed < - mr_median(mrin, weighting = "penalized")}

if(nrow(data)≥3){mregger < - mr_egger(mrin)}”

#### Power calculation

Calculations were performed to test whether our MR studies were adequately powered to detect a significant change mainly in the hip fracture outcomes but also in the fracture at any bone site outcome with the IVW method. For each trait, we used the variance explained by the instruments variables (R^2^ for continuous risk factors and available pseudo R^2^ for binary risk factors) reported in the GWAS publications, the proportion of fracture cases and the sample size, to estimate the power to detect different OR of 1.15 and 1.20 (α = 0.05/15 = 0.0033; http://cnsgenomics.com/shiny/mRnd/).^45^

## Data Availability

• The summary statistics of the present GWAS meta-analysis on hip fractures will be deposited to the GWAS catalog (*https://www.ebi.ac.uk/gwas/studies/GCST90161240*) upon publication of the article. Additional datasets that have been used for analysis can be found on the links to their corresponding reference papers. • This paper does not report original code. • Any additional information required to reanalyze the data reported in this work is available from the lead contact upon request.

## Supplemental information

Document S1. Figures S1–S4 and Tables S1–S10

Table S11. Association estimates for the genetic instruments used in the different Mendelian randomization analyses, related to Figure 1 and Table 3

Document S2. Article plus supplemental information
