# Supplementary material for: Assessment of the genetic and clinical determinants of hip fracture risk: Genome-wide association and Mendelian randomization study
Source: Cell Rep Med. 2022 Oct 18;3(10):100776. doi: 10.1016/j.xcrm.2022.100776 (PMC9589021; doi:10.1016/j.xcrm.2022.100776)
Supplement: Document S1. Figures S1–S4 and Tables S1–S10 [file mmc1.pdf]

Cell Reports Medicine, Volume *3*

Supplemental information

Assessment of the genetic and clinical determinants of hip fracture risk: Genome-wide association and Mendelian randomization study

Maria Nethander, Eivind Coward, Ene Reimann, Louise Grahnemo, Maiken E. Gabrielsen, Carl Wibom, Estonian Biobank Research Team, Reedik Mägi, Thomas Funck-Brentano, Mari Hoff, Arnulf Langhammer, Ulrika Pettersson-Kymmer, Kristian Hveem, and Claes Ohlsson

A

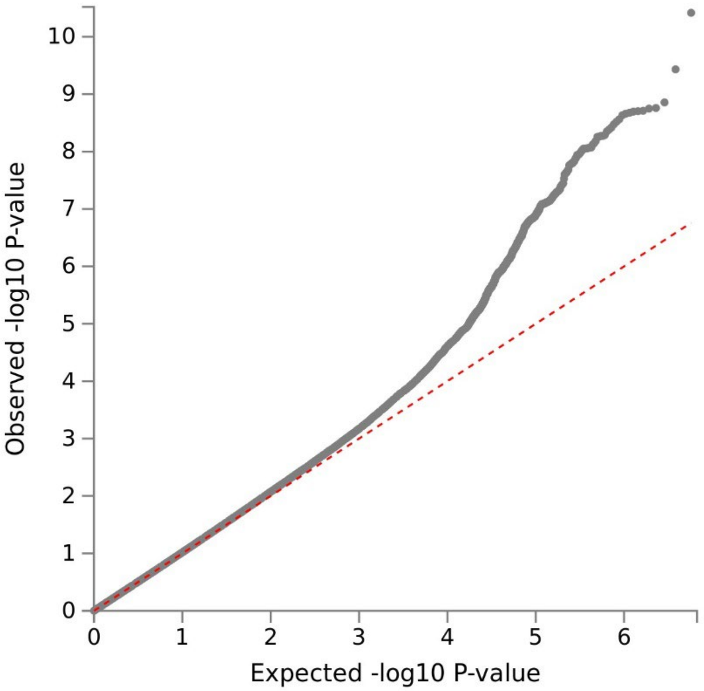

B

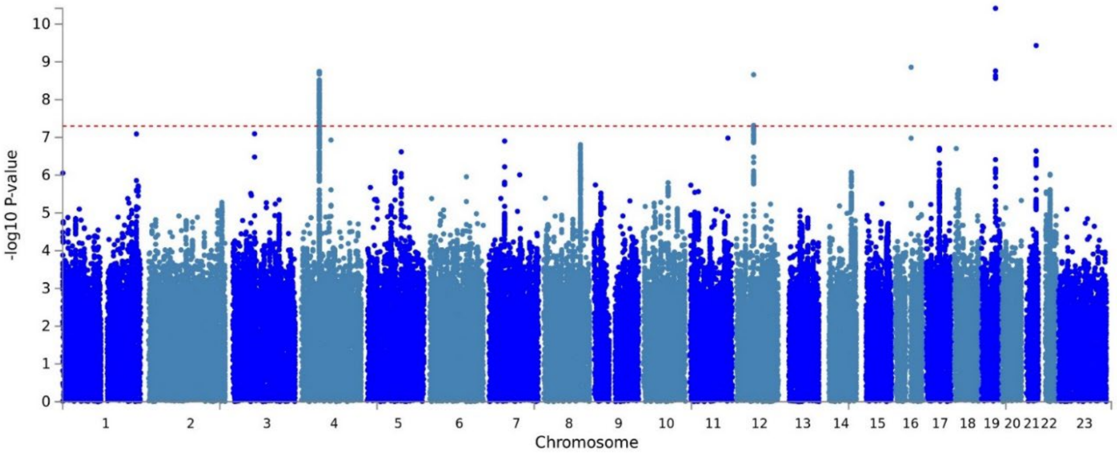

Figure S1: Quantile-quantile (Q-Q (A) and Manhattan Plot (B). Related to Table 1.

(A) Quantile-quantile (Q-Q) plot of observed versus expected P values of the GWAS results. The straight line in the Q-Q plot indicates the distribution of SNPs under the null hypothesis.

(B) Manhattan Plot of Association Statistics ($-\log_{10}(P)$) for hip fracture risk for the meta-analysis. Each dot represents a SNP, and the x axis indicates its chromosomal position (built 37 NCBI). Dashed horizontal red line marks the genome-wide significance threshold ($p < 5 \times 10^{-8}$).

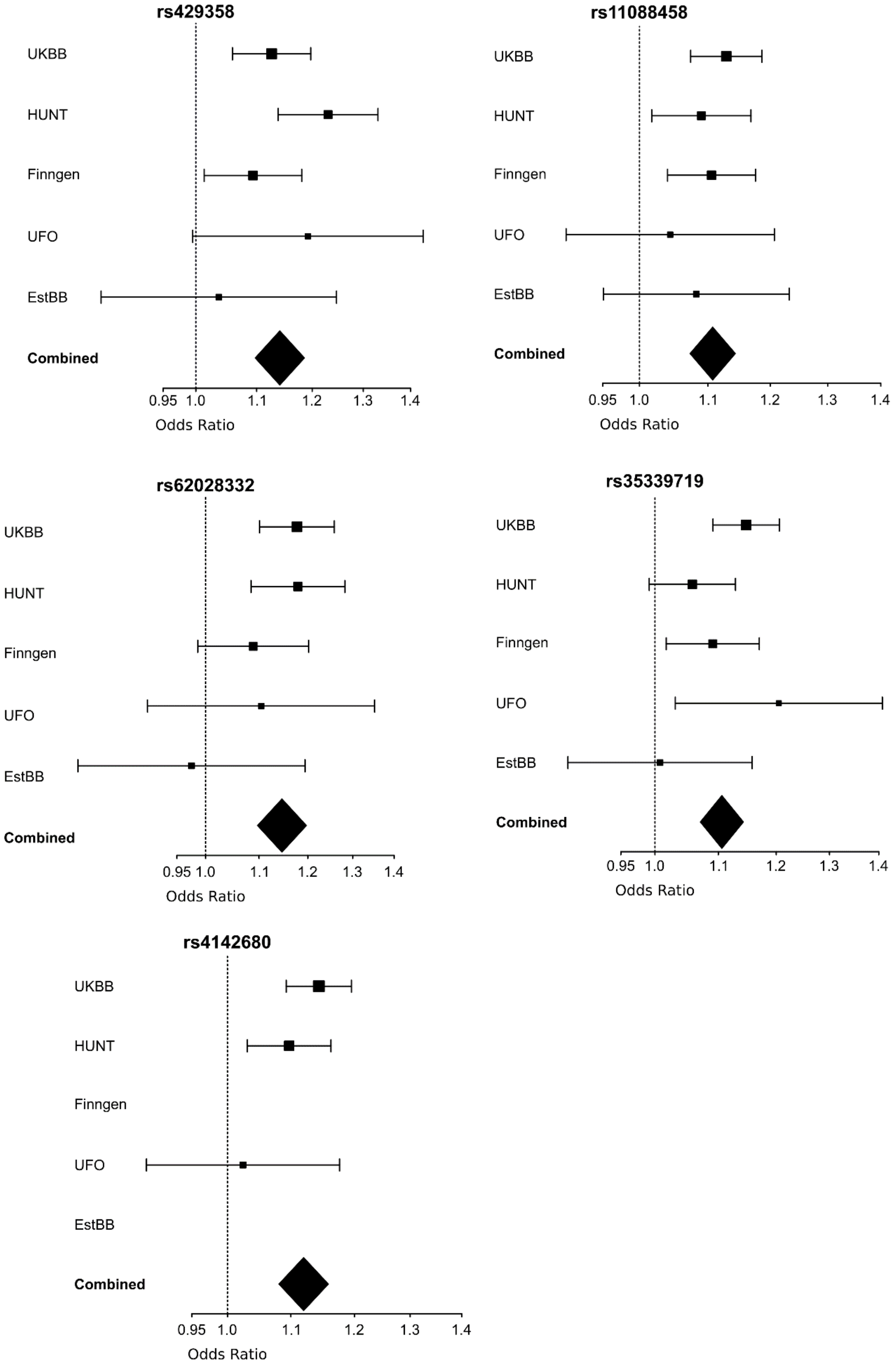

Figure S2: **Forest plots for the lead signal in each of the five loci associated with hip fracture risk.** Related to Table 1.

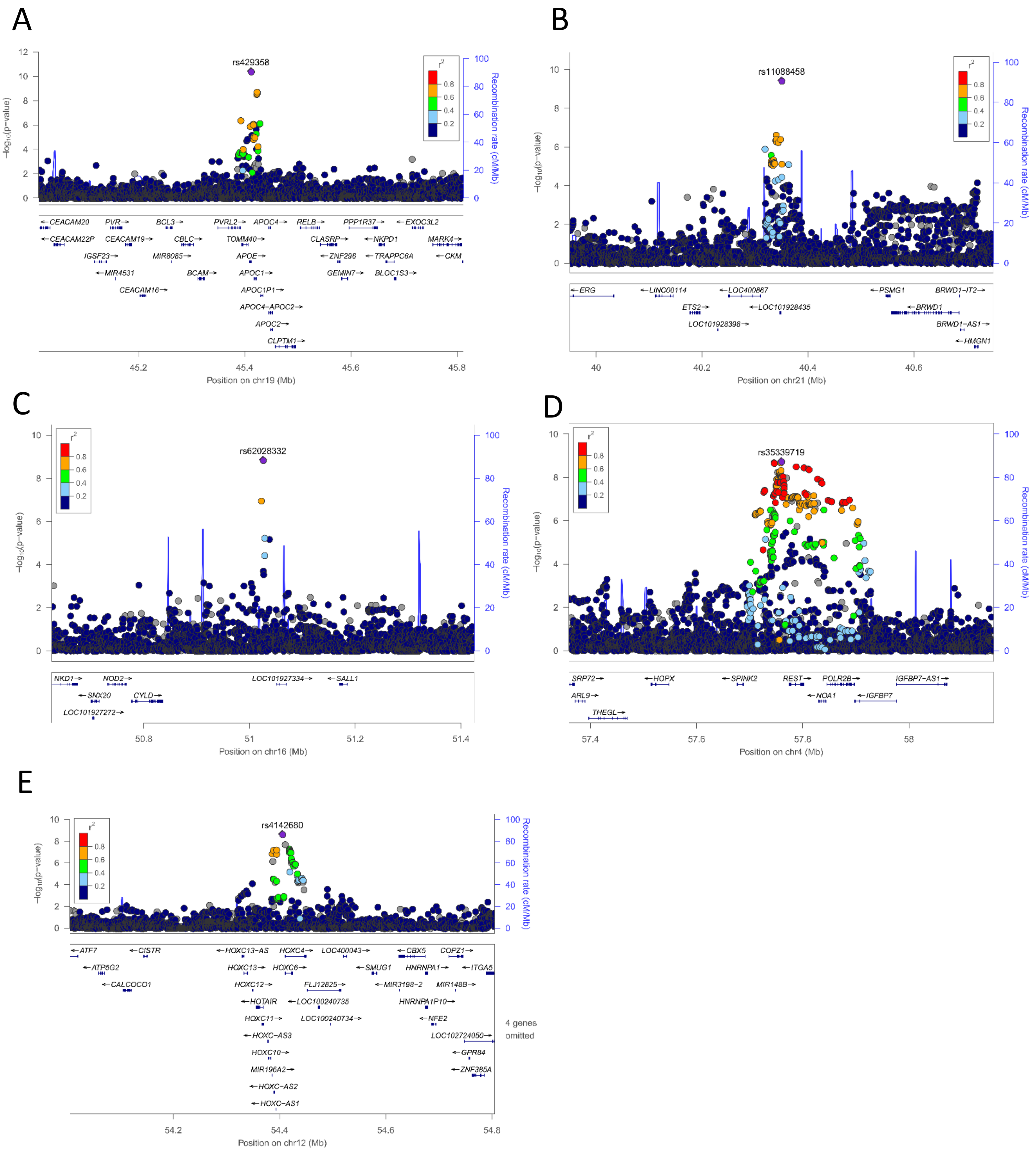

Figure S3: Regional association plots of top signals. Related to Table 1. (A). *APOE* region, 19q13.3219; (B). *ETS2* region 21q22.221; (C). *SALL1* region 16q12.116; (D). *REST* region 4q12; (E). *HOXC8* region 12q13.1312. SNPs are plotted by position in 500kb window against association with hip fracture risk (-log10 P). Plot highlighting the most significant SNPs in the hip fracture meta-analysis.

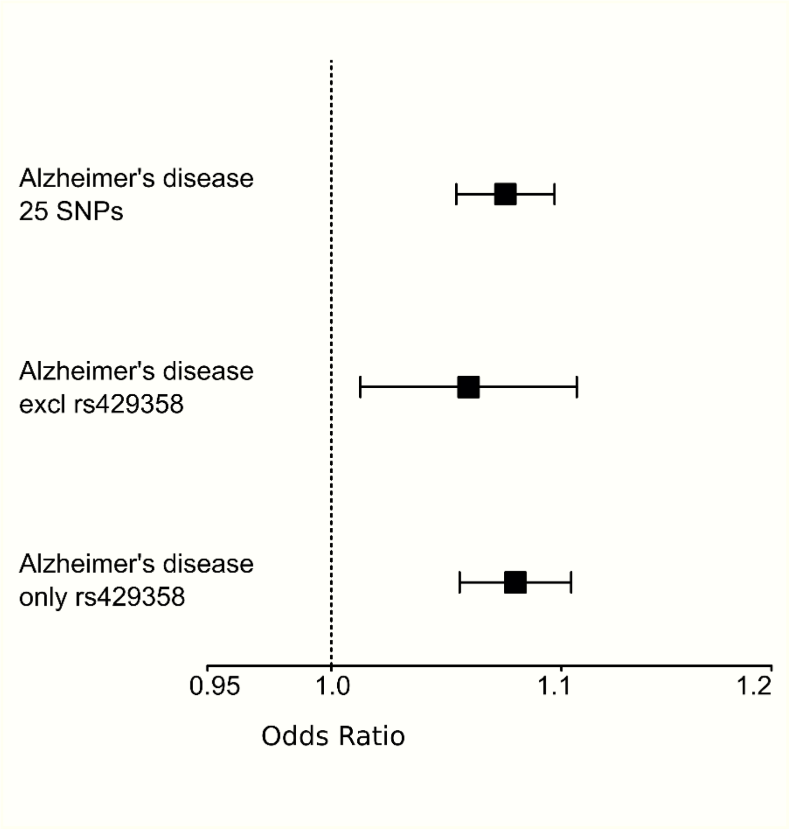

Figure S4: Mendelian randomization to estimate the causal effects of Alzheimer's disease on hip fractures. Related to Fig 1. (i) including all 25 available SNPs (=25 SNPs), (ii) excluding the strong signal in the *APOE*-region (=24 SNPs) or (iii) only using the strong SNP in the *APOE*-region (1 SNP). (Kunkle et al., 2019) Inverse variance weighted meta-analyses are performed. Odds ratios are for the risk of hip fracture per doubling of odds of Alzheimer's disease. Estimates are displayed using a random effects model to account for possible heterogeneity.

Table S1 Descriptive Study Design. Related to STAR Methods.

Short name	Full name	Study Design	Area of Origin	Ethnicity	Study Type	Total Sample Size with DNA, Covariates and Phenotype Information for the present study (N)	Study Description	References
**UKBB**	UK Biobank	Cohort study. Both prevalent and incident hip fractures were identified by ICD codes (ICD10, S72.0, S72.1, S72.2; ICD9, 820) from registers and fracture cases >30 years were included. Controls were defined as individuals from the same cohort without a history of hip fracture. Height and weight were derived from the UK-Biobank baseline visit. For fracture cases, age was the time for first hip fracture and for controls the age was the age of censoring (either death, emigration or time of evaluation in the registers). The UK Biobank has ethical approval from the Northwest Multicentre Research Ethics Committee, and informed consent was obtained from all participants.	UK	North-Western European subset used for analysis in the present study	Population based	438937	In 2006-2010, the UK Biobank recruited 502,643 individuals aged between 37 and 73 years (99.5% were 40-69 years) from across the country. Each participant provided a large amount of information regarding their health and lifestyle using touch screen questionnaires, physical measurements and agreement to have their health followed and they also provided blood, urine and saliva samples for future analysis.	Biobank UK. UK Biobank: Protocol for a Large-scale Prospective Epidemiological Resource. 2010. http://www.ukbiobank.ac.uk/wp-content/uploads/2011/11/UKBiobank-Protocol.pdf
**FinnGen**	FinnGen	The FinnGen study is a mixture of population-based cohorts and samples from hospital biobanks. Hip/femur fractures were identified by ICD codes. Both prevalent and incident hip fractures were identified by ICD codes (ICD10, S72; ICD9 820). We used publicly available summary statistics data from the 3rd data release from the FinnGen study (https://www.finngen.fi/en/access_results). The FinnGen analyses were adjusted for age, gender, genotyping chip, genetic relationship and first 10 PCs but not for height or weight. To achieve effect estimates not confounded by a possible minor dilution by diaphyseal and distal femur fractures and lack of adjustment for height and weight in the publicly available looked analyses of the FinnGen cohort, we replicated the significant causal associations in a meta-analysis excluding the FinnGen cohort, yielding similar effect estimates (Tables 1 and 3).	Finland	Northern European/Finnish	Population based	116375	FinnGen is a public-private partnership project combining genotype data from Finnish biobanks and digital health record data from Finnish health registries. FinnGen provides a unique opportunity to study genetic variation in relation to disease trajectories in an isolated population.<br>FinnGen is a growing project, aiming at 500,000 individuals in 2023. The data in the present study includes 116375 participants in the FinnGen, Data Freeze 3. FinnGen comprises prospective epidemiological cohorts (initiated as far back as 1992), disease-based cohorts, and hospital biobank samples. The unique national personal identification number links the genotypes to the national hospital discharge registry (1968-), the national death registry (1969-) and the medication reimbursement registry (1964-). These registries cover the whole population.	PMID33318493
**UFO**	Umeå Fracture and Osteoporosis Study	Case-Control study. Both prevalent and incident hip fractures were identified by ICD codes (ICD10, S72.0, S72.1, S72.2; ICD9, 820) and fracture cases >30 years were included. All fracture cases were also confirmed by radiological report and or medical records. Height and weight were retrieved from the closest visit and age is the age at the hip fracture. The UFO-hip fracture controls were age-matched without previous hip fracture and were drawn from the controls of a previous GWAS study of glioma (PMID 22886559). The UFO study was approved by the local research ethics committee at Umeå University. Written consent was obtained from all participants.	Sweden/Umeå	Northern European	Population based	1813	The UFO study is case-control study investigating associations between genes, lifestyle and osteoporotic fractures. The study is based on the prosepctive and populationbased Northern Sweden Health and Disease Study cohort, initiated to assess risk factors for diabetes and cardiovascular disease.	PMID20464545; PMID:1466024
**HUNT**	The Trøndelag Health Study	Cohort study. Both prevalent and incident hip fractures were identified by ICD codes (ICD10, S72.0, S72.1, S72.2; ICD9, 820) from registers and fracture cases >30 years were included. Controls were defined as individuals from the same cohorts, without a history of hip fracture. Height and weight were derived from the latest available HUNT visit. For fracture cases, age was the time for first hip fracture and for controls the age was the age at the HUNT visit for the height and weight measurements. Participation in the HUNT Study is based on informed consent and the study has been approved by the Regional Ethics Committee for Medical Research in Norway.	Norway/Trøndelag	Northern European	Population based	68819	The Trøndelag Health Study (HUNT) comprises data and samples obtained through four population studies between 1984 and 2019. 230,000 people from the county of Trøndelag have participated with questionnaire data, and and almost 120,000 participants have submitted biological samples. Even more than 120.000 have contributed with anthropometric measurements such as height, weight, blood pressure and many other measurements.	PMID22879362
**EstBB**	Estonian Biobank	Cohort study. Both prevalent and incident hip fractures were identified by ICD codes (ICD10, S72.0, S72.1, S72.2; ICD9, 820) from registers and fracture cases >30 years were included. Controls were defined as individuals from the same cohorts, without a history of hip fracture. Height and weight were derived from the latest visit. For fracture cases, age was the time for first hip fracture and for controls the age was age at recruitment to EstBB. Estonian Biobank has ethical approval from the Estonian Commitee on Bioethics and Human Research at the Ministry of Social Affairs (No 1.1-12/624). Written informed consent for participation was obtained from all study subjects.	Estonia/Tartu	Northern European	Population based	109410	The Estonian Biobank cohort is a volunteer-based sample of the Estonian resident adult population (aged ≥18 years). Estonians represent 83%, Russians 14%, and other nationalities 3% of all participants. The current number of participants is close to 200,000 and represents a large proportion, > 15 %, of the Estonian adult population, making it ideally suited to population-based studies. General practitioners (GPs) and medical personnel in the special recruitment offices have recruited participants throughout the country. At baseline, the GPs performed a standardized health examination of the participants, who also donated blood samples for DNA, white blood cells and plasma tests and filled out a 16-module questionnaire on health-related topics such as lifestyle, diet and clinical diagnoses described in WHO ICD-10. A significant part of the cohort has whole genome sequencing (3000), whole exome sequencing (2500), genome-wide single nucleotide polymorphism (SNP) array data (>200 000) and/or NMR metabolome data (11 000) available. The data are continuously updated through periodical linking to national electronic databases and registries. A part of the cohort has been re-contacted for follow-up purposes and resampling, and targeted invitations are possible for specific purposes, for example people with a specific diagnosis. For the current study data freeze including approximately 150,000 gene donors was applied, which resulted in including 109410 gene donors into the study.	PMID24518929, https://genomics.ut.ee/en/access-biobank

Table S2 Study-specific descriptive statistics. Related to STAR Methods.

		Women			Men		
**Short name**	**Trait**	**N**	**mean**	**SD**	**N**	**mean**	**SD**
**UKBB**	Age (yrs)	238565	68.3	7.9	200372	68.5	8.1
	Height (cm)	238565	162.6	6.2	200372	175.8	6.8
	Weight (kg)	238565	71.4	13.9	200372	86.2	14.3
**HUNT**	Age (yrs)	36377	52.73	18.14	32442	52.61	17.33
	Height (cm)	36377	164.1	6.51	32442	177.32	6.83
	Weight (kg)	36377	72.43	13.66	32442	85.47	13.39
**UFO**	Age (yrs)	1034	64.7	11.09	779	60.88	11.16
	Height (cm)	1034	163.4	5.96	779	176.74	6.67
	Weight (kg)	1034	68.0	12.68	779	81.45	12.05
**EstBB**	Age (yrs)	73617	43.65	15.90	35793	43.04	16.34
	Height (cm)	73617	166.00	6.36	35793	179.85	7.06
	Weight (kg)	73617	70.85	15.04	35793	86.39	15.63

For the public available **FinnGen** summary statistic data, the mean age = 60.6 yrs (SD 16.5 yrs) and 56.3% of the subjects are women. We do not have height or weight data for these subjects and we do not have age or gender stratified analyses for FinnGen.

Table S3 study-specific fracture counts. Related to STAR Methods.

Study	Fracture assessment method	Fracture cases (n)	Controls (n)	All (n)	Median age at fracture (yrs)
**UKBB**	Medical records; ICD codes; ICD10 S72.0; S72.1; S72.0; ICD9 820	4035	434902	438937	69
**HUNT**	Medical records; ICD codes; ICD10 S72.0; S72.1; S72.0; ICD9 820	3404	65415	68819	80
**UFO**	Medical records and radiographic verification ; ICD codes; ICD10 S72.0; S72.1; S72.0; ICD9 820	962	851	1813	64
**EstBB**	Medical records; ICD codes; ICD10 S72.0; S72.1; S72.0; ICD9 820	633	108777	109410	75
**FinnGen**	Medical records; ICD codes; ICD10 S72; ICD9 820	2482	113893	116375	NA
		11516	723838	735354	

NA = not available

Table S4 Genotypeing, study-specific fracture counts. Related to STAR Methods.

Cohort	Genotyping				Imputation			Association Analyses			
	Platform	Genotype Calling Algorithm	Call Rate	P for HWE	Imputation Software	Reference panel	Imputation Quality	MAF	Analysis Software	No. analyzed SNPs	λ
UK Biobank	UK Biobank Axiom Array (N~450,000) and UK BiLEVE Array (N~50,000)	Affymetrix Power Tools software and the Affymetrix Best Practices Workflow	≥90%	$>10^{-12}$	Phasing: Modified version of SHAPEIT2; Imputation: modified version of IMPUTE2	HRC v1.1 and UK10K + 1000 Genomes panel	≥0.3	>1%	SAIGE	9 576 610	1.00
HUNT	Illumina HumanCoreExome arrays: 12 v1.0, 12 v1.1, UM HUNT Biobank v1.0 (HumanCoreExome 24 with custom content)	Illumina GenomeStudio	≥99%	$>10^{-4}$	Minimac3	HRC v1.1 and 2201 WGS samples from HUNT	≥0.3	>1%	SAIGE	8 825 549	1.02
FinnGen	Illumina (Illumina Inc., San Diego, CA, USA) and Affymetrix arrays (Thermo Fisher Scientific, Santa Clara, CA, USA)	GenCall and zCall algorithms for Illumina and AxiomGT1 algorithm for Affymetrix data	≥95%	$>10^{-6}$	Pre-phased using Eagle 2.3.5, imputed using Beagle 4.1 (version 08Jun17.d8b)	SISu v3 imputation reference panel	≥0.95	>1%	SAIGE	8 696 849	0.99
EstBB	Illumina GSAv1.0, GSAv2.0, and GSAv2.0_EST arrays	Illumina GenomeStudio v2.0.4	≥95%	$>10^{-4}$	Pre-phased using Eagle v2.3, imputed using Beagle v.28Sep18.793	Estonian population specific imputation reference of 2297 WGS samples were used	≥0.3	>1%	SAIGE	8 664 150	0.92
UFO	Illumina HumanHap660 arrays	BeadStudio	≥97.5%	$>10^{-6}$	Pre-phased using Eagle v2.4, imputed using PBWT	HRC v1.1	≥0.3	>1%	PLINK	8 002 885	1.06

Table S5 Genome wide significant nucleotide polymorphisms for hip fractures combined in the meta-analyseis and for each included cohort. Related to Table 1.

Locus	Candidate Gene	Distance to gene (kb)	SNP	CHR	EA	OA	EAF	EAFSE	GWAS meta-analyses N	GWAS meta-analyses Ncases	GWAS meta-analyses OR	GWAS meta-analyses (95% CI)	GWAS meta-analyses P	Heterozygosity I^2	Heterozygosity P
19q13.32	*APOE*	0.0	rs429358	19	C	T	0.17	0.02	735354	11516	1.14	(1.10 - 1.19)	3.8E-11	34.6	0.19
21q22.2	*ETS2*	153.2	rs11088458	21	G	A	0.70	0.04	735354	11516	1.11	(1.07 - 1.14)	3.7E-10	0	0.83
16q12.1	*SALL1*	144.4	rs62028332	16	G	A	0.87	0.02	735354	11516	1.15	(1.10 - 1.20)	1.4E-09	13.9	0.33
4q12	*REST*	14.5	rs35339719	4	G	A	0.74	0.02	735354	11516	1.11	(1.07 - 1.14)	1.8E-09	41.3	0.15
12q13.13	*HOXC8*	0.0	rs4142680	12	T	C	0.41	0.01	509569	8401	1.12	(1.08 - 1.16)	2.2E-09	26.5	0.26

Locus	UKBB OR	UKBB 95% CI	UKBB P	UKBB N	UKBB Ncases	HUNT OR	HUNT 95% CI	HUNT P	HUNT N	HUNT Ncases
19q13.32	1.13	(1.06 - 1.20)	1.5E-04	438937	4035	1.23	(1.14 - 1.33)	2.1E-07	68819	3404
21q22.2	1.13	(1.07 - 1.19)	1.8E-06	438937	4035	1.09	(1.02 - 1.17)	1.5E-02	68819	3404
16q12.1	1.18	(1.10 - 1.26)	1.7E-06	438937	4035	1.18	(1.08 - 1.28)	1.2E-04	68819	3404
4q12	1.15	(1.09 - 1.21)	7.2E-08	438937	4035	1.06	(0.99 - 1.13)	8.9E-02	68819	3404
12q13.13	1.14	(1.09 - 1.19)	3.9E-08	438937	4035	1.09	(1.03 - 1.16)	3.9E-03	68819	3404

Locus	FinnGen OR	FinnGen 95% CI	FinnGen P	FinnGen N	FinnGen Ncases	UFO OR	UFO 95% CI	UFO P	UFO N	UFO Ncases	EstBB OR	EstBB 95% CI	EstBB P	EstBB N	EstBB Ncases
19q13.32	1.09	(1.01 - 1.18)	2.2E-02	116375	2482	1.19	(1.00 - 1.43)	5.6E-02	1813	962	1.04	(0.86 - 1.25)	7.0E-01	109410	633
21q22.2	1.11	(1.04 - 1.18)	1.3E-03	116375	2482	1.04	(0.90 - 1.21)	5.6E-01	1813	962	1.08	(0.95 - 1.23)	2.3E-01	109410	633
16q12.1	1.09	(0.99 - 1.20)	9.3E-02	116375	2482	1.10	(0.90 - 1.35)	3.4E-01	1813	962	0.98	(0.80 - 1.19)	8.1E-01	109410	633
4q12	1.09	(1.02 - 1.17)	1.5E-02	116375	2482	1.20	(1.03 - 1.41)	1.9E-02	1813	962	1.01	(0.88 - 1.16)	9.1E-01	109410	633
12q13.13	NA	NA	NA	NA	NA	1.02	(0.89 - 1.17)	7.5E-01	1813	962	NA	NA	NA	NA	NA

OR = Odds ratio NA = not available, EA = effect allele, EAF Effect allele frequency, OA = other allele, I^2 = index of heterogenity, CHR = chromosome, SNP = single nucleotide polymorphism, CI = confidence interval

Table S6 Number of SNPs and Variance explained by Instrumental Variables for Each Risk Factor. Related to Fig 1.

Trait or disease	N SNPs	Total R^2	Total Sample Size in reference GWAS	PMID
Continuous risk factor				
**BMD-related parameters**				
Decreased FN-BMD	47	5.8	32 961	22504420
Decreased LS-BMD	45	6.4	31 800	22504420
Decreased eBMD	457	20	426 824	30598549
**Other risk markers**				
Early menopause	54	6	69 360	26414677 + 30158200
Late puberty	106	2.7	182 416	25231870 + 30158200
Decreased TSH	20	5.6	26 523	23408906 + 30158200
Decreased grip strength/BW	130	1.7	223 315	29691431
Low vitamin D levels	103	7.5	417 580	32242144
Binary risk factor				
Alzheimer´s disease	25	31	63926 (21982 cases)	30820047
Coronary heart disease	38	5.7	107 432 (41 513 cases)	23202125 + 30158200
Rheumatoid arhritis	57	5.5	58 284 (14 361 cases)	24390342
Inflammatory bowel disease	149	7.5	34 652 (12 882 cases)	23128233 + 30158200
Type 1 diabetes	19	6.7	26 890 (9934 cases)	21980299 + 30158200
Type 2 diabetes	38	5.7	56 862 (12 171 cases)	22885922 + 30158200
Ever smoked regularly	367	2.3	1 232 091 (NA)	30643251
Risk factors with insufficient power for MR analyses				
Falls	3	0.28	362 103 (89 076 cases)	32999390
Alcohol Consumption	96	0.2	941 280	30643251

NA= not available, BMD = bone mineral density, TSH = Thyroid-stimulating hormone, FN = femoral neck, LS = lumbar spine, eBMD = estimated BMD in the heel using ultrasound

Table S7 Mendelian randomization to estimate the causal effects of 15 genetically determined risk factors on hip fracture and fracture at any bone site using a variety of MR methods. Related to Fig 1.

Hip fracture

			CochranQ		Inverse variance weighted fixed effects			Inverse variance weighted random effects			Weighted median			Penalized weighted median			Egger Intercept		Egger Estimate		
**Trait or disease**	**N SNPs**	**PMID**	**Est**	**P**	**OR**	**95% CI**	**P**	**OR**	**95% CI**	**P**	**OR**	**95% CI**	**P**	**OR**	**95% CI**	**P**	**Beta**	**P**	**OR**	**95% CI**	**P**
Continuous risk factor																					
**BMD-related parameters**																					
Decreased FN-BMD	47	22504420	117.30	**3.8E-08**	2.12	(1.93 - 2.33)	**6.7E-54**	2.12	(1.82 - 2.47)	**3.7E-22**	2.03	(1.72 - 2.40)	**5.6E-17**	2.00	(1.70 - 2.37)	**3.3E-16**	0.02	1.2E-01	1.49	(0.93 - 2.38)	9.9E-02
Decreased LS-BMD	45	22504420	194.69	**7.5E-21**	1.66	(1.52 - 1.82)	**3.6E-29**	1.66	(1.38 - 2.00)	**9.9E-08**	1.55	(1.31 - 1.83)	**2.6E-07**	1.59	(1.34 - 1.88)	**7.7E-08**	0.05	**5.5E-04**	0.71	(0.43 - 1.19)	1.9E-01
Decreased eBMD	457	30598549	925.89	**3.2E-34**	1.73	(1.63 - 1.83)	**1.0E-78**	1.73	(1.59 - 1.87)	**1.1E-39**	1.73	(1.54 - 1.94)	**5.6E-21**	1.87	(1.67 - 2.09)	**5.5E-28**	0.00	6.4E-02	1.55	(1.35 - 1.78)	**6.3E-10**
**Other risk markers**																					
Early menopause	54	26414677	40.46	9.0E-01	0.95	(0.87 - 1.04)	3.0E-01	0.95	(0.87 - 1.04)	3.0E-01	0.97	(0.84 - 1.12)	7.0E-01	0.97	(0.84 - 1.12)	7.1E-01	-0.01	2.3E-01	1.08	(0.86 - 1.36)	4.9E-01
Only females	54	26414677	57.68	3.1E-01	1.01	(0.89 - 1.15)	8.4E-01	1.01	(0.88 - 1.16)	8.5E-01	1.02	(0.82 - 1.26)	8.9E-01	1.02	(0.82 - 1.26)	8.8E-01	-0.01	9.6E-02	1.30	(0.94 - 1.80)	1.1E-01
Late puberty	106	25231870	131.73	4.0E-02	1.13	(1.02 - 1.26)	1.7E-02	1.13	(1.01 - 1.27)	3.3E-02	1.26	(1.08 - 1.48)	4.0E-03	1.28	(1.09 - 1.50)	**2.6E-03**	-0.01	3.2E-02	1.64	(1.15 - 2.35)	6.5E-03
Only females	106	25231870	109.70	3.6E-01	1.18	(1.02 - 1.37)	2.5E-02	1.18	(1.02 - 1.38)	2.8E-02	1.45	(1.14 - 1.85)	**2.8E-03**	1.47	(1.15 - 1.88)	**2.3E-03**	-0.01	2.5E-01	1.55	(0.96 - 2.50)	7.3E-02
Onlly males	106	25231870	113.16	2.8E-01	1.02	(0.84 - 1.24)	8.7E-01	1.02	(0.83 - 1.25)	8.7E-01	1.19	(0.88 - 1.61)	2.7E-01	1.19	(0.88 - 1.61)	2.6E-01	-0.03	4.1E-03	2.42	(1.30 - 4.51)	5.4E-03
Decreased TSH	20	23408906	21.42	3.1E-01	1.01	(0.92 - 1.11)	8.5E-01	1.01	(0.91 - 1.11)	8.6E-01	0.93	(0.82 - 1.07)	3.2E-01	0.93	(0.81 - 1.06)	2.9E-01	0.01	4.8E-01	0.90	(0.65 - 1.26)	5.4E-01
Decreased grip strength/BW	130	29691431	192.11	**2.6E-04**	1.06	(0.90 - 1.25)	4.8E-01	1.06	(0.87 - 1.30)	5.6E-01	1.04	(0.80 - 1.35)	7.8E-01	1.02	(0.78 - 1.33)	9.1E-01	-0.01	3.8E-01	1.56	(0.64 - 3.78)	3.3E-01
Low vitamin D levels	103	32242144	142.54	5.0E-03	0.98	(0.88 - 1.08)	6.6E-01	0.98	(0.87 - 1.10)	7.1E-01	0.99	(0.85 - 1.15)	9.0E-01	0.99	(0.85 - 1.15)	9.0E-01	0.00	3.8E-01	1.02	(0.87 - 1.19)	7.9E-01
Binary risk factor																					
Alzheimer´s disease	25	30820047	17.85	8.1E-01	1.07	(1.05 - 1.10)	**1.9E-12**	1.07	(1.05 - 1.10)	**1.9E-12**	1.08	(1.06 - 1.10)	**2.1E-11**	1.08	(1.06 - 1.10)	**2.1E-11**	0.00	7.2E-01	1.08	(1.05 - 1.10)	**3.6E-09**
Coronar heart disease	38	23202125	61.13	7.5E-03	1.01	(0.96 - 1.07)	5.9E-01	1.01	(0.95 - 1.08)	6.7E-01	0.98	(0.91 - 1.06)	6.6E-01	0.97	(0.90 - 1.05)	4.9E-01	-0.01	4.0E-01	1.11	(0.90 - 1.36)	3.5E-01
Rheumatoid arhritis	57	24390342	55.53	4.9E-01	1.00	(0.97 - 1.02)	9.3E-01	1.00	(0.97 - 1.02)	9.3E-01	1.00	(0.96 - 1.04)	9.1E-01	1.00	(0.97 - 1.04)	8.8E-01	0.00	6.3E-01	1.01	(0.96 - 1.06)	7.1E-01
Inflamatory bowel disease	149	23128233	179.65	3.9E-02	1.01	(1.00 - 1.03)	8.9E-02	1.01	(1.00 - 1.03)	1.2E-01	0.99	(0.97 - 1.02)	6.2E-01	0.99	(0.96 - 1.01)	2.8E-01	0.00	3.6E-01	1.00	(0.96 - 1.04)	9.2E-01
Type 1 diabetes	19	21980299	26.64	8.6E-02	1.00	(0.99 - 1.02)	8.2E-01	1.00	(0.98 - 1.02)	8.6E-01	1.00	(0.98 - 1.02)	9.8E-01	1.00	(0.98 - 1.02)	1.0E+00	0.00	5.5E-01	1.01	(0.98 - 1.04)	5.8E-01
Type 2 diabetes	38	22885922	44.31	1.9E-01	1.03	(0.99 - 1.06)	1.4E-01	1.03	(0.99 - 1.06)	1.8E-01	1.01	(0.96 - 1.07)	5.9E-01	1.01	(0.96 - 1.06)	7.3E-01	0.01	2.2E-01	0.97	(0.89 - 1.07)	5.6E-01
Ever smoked regularly	367	30643251	440.65	4.5E-03	1.08	(1.03 - 1.13)	**8.2E-04**	1.08	(1.03 - 1.13)	**2.3E-03**	1.04	(0.97 - 1.11)	3.1E-01	1.03	(0.96 - 1.10)	4.1E-01	0.00	7.6E-01	1.05	(0.85 - 1.29)	6.7E-01

Fracture at any bone site

	CochranQ		Inverse variance weighted fixed effects			Inverse variance weighted random effects			Weighted median			Penalized weighted median			Egger Intercept		Egger Estimate		
**Trait or disease**	**Est**	**P**	**OR**	**95% CI**	**P**	**OR**	**95% CI**	**P**	**OR**	**95% CI**	**P**	**OR**	**95% CI**	**P**	**Beta**	**P**	**OR**	**95% CI**	**P**
Continuous risk factor																			
**BMD-related parameters**																			
Decreased FN-BMD	382.23	**1.5E-54**	1.63	(1.56 - 1.70)	**3.6E-114**	1.63	(1.44 - 1.84)	**3.3E-15**	1.42	(1.31 - 1.54)	**3.5E-18**	1.37	(1.26 - 1.48)	**2.9E-15**	0.01	4.4E-01	1.41	(0.97 - 2.06)	7.4E-02
Decreased LS-BMD	396.11	**3.6E-58**	1.56	(1.50 - 1.62)	**1.1E-111**	1.56	(1.39 - 1.76)	**7.2E-14**	1.46	(1.35 - 1.58)	**5.5E-22**	1.39	(1.29 - 1.51)	**3.1E-17**	0.01	4.1E-01	1.36	(0.95 - 1.93)	9.0E-02
Decreased eBMD	1040.18	**5.8E-45**	1.65	(1.61 - 1.69)	**< 2.2E-308**	1.65	(1.59 - 1.71)	**3.0E-151**	1.76	(1.67 - 1.84)	**5.4E-115**	1.78	(1.69 - 1.88)	**1.0E-98**	0.00	8.5E-01	1.66	(1.55 - 1.76)	**< 2.2E-308**
**Other risk markers**																			
Early menopause	55.64	3.8E-01	1.07	(1.03 - 1.12)	**6.7E-04**	1.07	(1.03 - 1.12)	**9.0E-04**	1.08	(1.01 - 1.15)	1.6E-02	1.08	(1.01 - 1.15)	1.6E-02	0.00	8.8E-01	1.08	(0.98 - 1.20)	1.4E-01
Only females																			
Late puberty	155.75	**9.6E-04**	1.07	(1.02 - 1.12)	6.2E-03	1.07	(1.01 - 1.13)	2.5E-02	1.08	(0.99 - 1.16)	7.0E-02	1.08	(0.99 - 1.16)	7.0E-02	0.00	8.5E-01	1.05	(0.88 - 1.25)	6.1E-01
Only females																			
Onlly males																			
Decreased TSH	15.73	6.7E-01	0.99	(0.95 - 1.03)	6.2E-01	0.99	(0.95 - 1.03)	6.2E-01	1.01	(0.95 - 1.07)	8.1E-01	1.01	(0.95 - 1.07)	8.1E-01	0.00	8.1E-01	1.01	(0.88 - 1.15)	9.4E-01
Decreased grip strength/BW	268.17	**8.5E-12**	1.21	(1.13 - 1.30)	**2.4E-07**	1.21	(1.09 - 1.34)	**3.4E-04**	1.21	(1.07 - 1.35)	**1.4E-03**	1.21	(1.08 - 1.35)	**1.2E-03**	0.00	2.4E-01	0.93	(0.59 - 1.46)	7.5E-01
Low vitamin D levels	112.51	2.9E-02	0.99	(0.94 - 1.03)	5.4E-01	0.99	(0.94 - 1.04)	5.9E-01	1.00	(0.93 - 1.06)	8.9E-01	1.00	(0.93 - 1.06)	9.0E-01	0.00	1.8E-01	1.01	(0.95 - 1.08)	6.9E-01
Binary risk factor																			
Alzheimer´s disease	19.87	7.0E-01	1.00	(0.99 - 1.01)	6.5E-01	1.00	(0.99 - 1.01)	6.5E-01	1.00	(0.99 - 1.01)	5.5E-01	1.00	(0.99 - 1.01)	5.5E-01	0.00	7.6E-01	1.00	(0.99 - 1.01)	5.9E-01
Coronar heart disease	39.69	3.5E-01	1.01	(0.99 - 1.03)	4.1E-01	1.01	(0.99 - 1.03)	4.3E-01	1.02	(0.98 - 1.05)	3.5E-01	1.02	(0.98 - 1.05)	3.5E-01	0.00	2.6E-01	0.97	(0.90 - 1.04)	4.0E-01
Rheumatoid arhritis	77.13	3.2E-02	1.01	(1.00 - 1.02)	5.6E-02	1.01	(1.00 - 1.02)	1.0E-01	1.01	(0.99 - 1.02)	5.1E-01	1.01	(0.99 - 1.02)	5.0E-01	0.00	4.3E-01	1.00	(0.98 - 1.03)	8.7E-01
Inflamatory bowel disease	331.35	**4.5E-16**	1.00	(0.99 - 1.01)	6.7E-01	1.00	(0.99 - 1.01)	7.8E-01	1.01	(0.99 - 1.02)	3.1E-01	1.01	(1.00 - 1.02)	2.1E-01	0.00	8.9E-01	1.00	(0.98 - 1.02)	8.0E-01
Type 1 diabetes	13.87	7.4E-01	1.00	(0.99 - 1.00)	6.6E-01	1.00	(0.99 - 1.00)	6.6E-01	1.00	(0.99 - 1.01)	9.5E-01	1.00	(0.99 - 1.01)	9.5E-01	0.00	8.4E-01	1.00	(0.99 - 1.01)	8.5E-01
Type 2 diabetes	69.05	**1.1E-03**	1.00	(0.99 - 1.02)	5.1E-01	1.00	(0.98 - 1.03)	6.3E-01	1.02	(1.00 - 1.05)	7.4E-02	1.02	(1.00 - 1.05)	7.8E-02	-0.01	7.6E-02	1.05	(1.00 - 1.10)	6.9E-02
Ever smoked regularly	565.40	**1.5E-10**	1.05	(1.03 - 1.07)	**2.2E-06**	1.05	(1.02 - 1.07)	**1.4E-04**	1.05	(1.02 - 1.09)	**7.2E-04**	1.06	(1.03 - 1.10)	**9.3E-05**	0.00	2.3E-01	1.11	(1.01 - 1.23)	3.8E-02

Several different MR methods are displayed, including Inverse variance weighted, Weighted median, Penalized median and Egger regression. Odds ratio (OR) is for the risk of fracture per standard deviation (SD) change in the risk factor for continuous trait or risk of fracture per doubling of odds of disease susceptibility for binary factors. For ever smoked regularly, the ORs are expressed per 0.5 unit increase in log odds of ever smoking regularly with a 1 SD increase in genetically predicted smoking initiation corresponding to a 10% increased risk of smoking (PMID30643251 + PMID33253141).

Findings that remain associated after correction for multiple testing (0.05/15; $\alpha<3.3\times10^{-3}$) are given in bold. Grip strength is given as grip strength per body weight (BW). SD for grip strength is given for kg grip strength per kg in body weight and was estimated in the UK-Biobank to be 0.127. NA=not available; BMD=bone mineral density. Estimates for the association with hip fracture is from the present hip fracture GWAS while the estimates from any fracture is from Morris *et al* Nature Genetics 2019 (PMID30598549). TSH = Thyroid-stimulating hormone, FN = femoral neck, LS = lumbar spine, eBMD = estimated BMD in the heel using ultrasound.

Table S8 Association for femoral neck bone mineral density GWAS significant loci with hip fracture risk. Related to Table 1 and Fig 1.

			FN-BMD				Hip fracture			
**SNP**	**EA**	**OA**	**EAF**	**Beta**	**SE**	**P**	**EAF**	**OR**	**95%CI**	**P**
rs736825	C	G	0.56	0.04	0.01	1.1E-09	0.59	0.92	(0.90 - 0.95)	**8.9E-08**
rs1566045	C	T	0.20	0.06	0.01	1.9E-22	0.19	0.90	(0.87 - 0.94)	**1.1E-07**
rs1366594	A	C	0.54	0.08	0.00	4.5E-61	0.55	0.93	(0.90 - 0.96)	**8.1E-07**
rs4792909	T	G	0.37	0.04	0.01	2.0E-11	0.41	0.93	(0.90 - 0.96)	**1.5E-06**
rs1286083	C	T	0.19	0.05	0.01	2.0E-15	0.17	0.91	(0.88 - 0.95)	**3.0E-06**
rs7108738	G	T	0.17	0.08	0.01	1.1E-32	0.22	0.92	(0.89 - 0.96)	**9.5E-06**
rs4796995	A	G	0.63	0.03	0.01	4.9E-08	0.63	0.94	(0.91 - 0.96)	**1.2E-05**
rs11623869	G	T	0.65	0.04	0.00	5.2E-16	0.66	0.94	(0.91 - 0.97)	**6.7E-05**
rs9466056	G	A	0.62	0.04	0.01	2.7E-13	0.63	0.94	(0.92 - 0.97)	**1.1E-04**
rs430727	C	T	0.52	0.06	0.01	4.4E-25	0.56	0.95	(0.92 - 0.97)	**1.4E-04**
rs6532023	T	G	0.34	0.06	0.01	5.0E-26	0.32	0.95	(0.92 - 0.97)	**3.3E-04**
rs6959212	C	T	0.68	0.04	0.01	1.2E-13	0.68	0.95	(0.92 - 0.98)	**3.9E-04**
rs3790160	T	C	0.50	0.04	0.01	3.6E-12	0.49	0.95	(0.93 - 0.98)	**1.4E-03**
rs7521902	C	A	0.69	0.04	0.01	2.9E-09	0.75	0.95	(0.92 - 0.98)	**1.5E-03**
rs7751941	G	A	0.79	0.04	0.01	1.6E-09	0.79	0.95	(0.91 - 0.98)	**3.7E-03**
rs4790881	A	C	0.69	0.05	0.01	9.8E-19	0.69	0.96	(0.93 - 0.99)	**4.0E-03**
rs7953528	A	T	0.18	0.05	0.01	1.9E-12	0.17	0.95	(0.91 - 0.99)	**6.9E-03**
rs1373004	G	T	0.87	0.04	0.01	1.5E-08	0.88	0.94	(0.90 - 0.98)	**7.1E-03**
rs2062377	T	A	0.43	0.06	0.01	9.1E-25	0.43	0.96	(0.93 - 0.99)	**8.6E-03**
rs3736228	C	T	0.84	0.05	0.01	4.8E-11	0.87	0.94	(0.90 - 0.99)	**1.0E-02**
rs6426749	C	G	0.17	0.11	0.01	7.4E-57	0.15	0.95	(0.91 - 0.99)	**1.2E-02**
rs13336428	G	A	0.57	0.04	0.00	1.5E-16	0.59	0.97	(0.94 - 1.00)	**2.3E-02**
rs9921222	C	T	0.52	0.04	0.01	5.2E-12	0.52	0.97	(0.94 - 1.00)	**2.4E-02**
rs4985155	G	A	0.33	0.03	0.00	1.7E-10	0.34	0.97	(0.94 - 1.00)	**2.8E-02**
rs7812088	A	G	0.13	0.05	0.01	7.3E-09	0.13	0.96	(0.92 - 1.00)	**4.0E-02**
rs10048146	A	G	0.80	0.05	0.01	1.0E-14	0.80	0.97	(0.93 - 1.00)	5.3E-02
rs4727338	C	G	0.67	0.08	0.01	8.1E-48	0.68	0.97	(0.94 - 1.00)	5.4E-02
rs7851693	C	G	0.64	0.05	0.01	3.4E-22	0.63	0.97	(0.94 - 1.00)	6.0E-02
rs7932354	T	C	0.31	0.05	0.01	5.1E-18	0.31	0.97	(0.94 - 1.00)	6.8E-02
rs4869742	C	T	0.69	0.05	0.01	4.2E-18	0.72	0.97	(0.93 - 1.01)	9.3E-02
rs7217932	A	G	0.46	0.03	0.00	1.9E-11	0.48	0.98	(0.95 - 1.01)	1.2E-01
rs2887571	G	A	0.24	0.03	0.01	6.5E-09	0.23	0.97	(0.94 - 1.01)	1.3E-01
rs163879	C	T	0.32	0.03	0.01	2.1E-08	0.35	0.98	(0.95 - 1.01)	1.8E-01
rs1346004	G	A	0.50	0.05	0.00	1.1E-25	0.49	0.98	(0.95 - 1.01)	2.0E-01
rs7584262	T	C	0.23	0.04	0.01	1.3E-09	0.23	0.99	(0.95 - 1.02)	4.0E-01
rs1053051	C	T	0.48	0.03	0.00	9.6E-10	0.48	0.99	(0.96 - 1.02)	4.4E-01
rs9533090	C	T	0.51	0.05	0.01	4.9E-23	0.49	0.99	(0.96 - 1.02)	4.5E-01
rs17040773	A	C	0.76	0.04	0.01	1.5E-09	0.79	0.99	(0.95 - 1.02)	4.7E-01
rs1026364	T	G	0.37	0.03	0.00	4.1E-10	0.39	1.01	(0.98 - 1.04)	5.7E-01
rs7084921	T	C	0.39	0.03	0.00	9.0E-10	0.37	0.99	(0.96 - 1.02)	5.9E-01
rs13204965	A	C	0.76	0.04	0.01	8.1E-12	0.76	0.99	(0.96 - 1.02)	5.9E-01
rs479336	G	T	0.26	0.04	0.01	8.5E-15	0.26	0.99	(0.96 - 1.02)	5.9E-01
rs3801387	G	A	0.26	0.08	0.01	5.0E-40	0.26	1.01	(0.98 - 1.04)	6.0E-01
rs884205	C	A	0.73	0.04	0.01	3.2E-10	0.73	0.99	(0.96 - 1.03)	6.5E-01
rs3755955	G	A	0.84	0.06	0.01	1.5E-14	0.85	0.99	(0.95 - 1.03)	7.0E-01
rs12407028	T	C	0.60	0.05	0.01	3.4E-23	0.58	1.00	(0.97 - 1.02)	7.4E-01
rs2016266	G	A	0.32	0.03	0.00	3.7E-10	0.32	1.00	(0.97 - 1.03)	7.8E-01

EA = effect allele, OA= other allele. Effect estimates for femoral neck bone mineral density (FN-BMD) are derived from Estrada et al (PMID 2250442).

Table S9 Association for Alzheimer's disease GWAS significant loci with hip fracture risk. Related to Fig 1.

					Alzheimer's disease						Hip fracture					
SNP	POS	Gene	EA	OA	EAF	Beta	SE	OR	95%CI	P	EAF	Beta	SE	OR	95%CI	P
rs429358	chr19:44908684	APOE	C	T	0.22	1.20	0.02	3.32	(3.20 - 3.45)	1.2E-881	0.17	0.13	0.02	1.14	(1.10 - 1.19)	3.8E-11
rs9473117	chr6:47463548	CD2AP	C	A	0.28	0.09	0.01	1.09	(1.06 - 1.12)	1.2E-10	0.26	0.04	0.02	1.04	(1.00 - 1.07)	2.9E-02
rs3851179	chr11:86157598	EED	C	T	0.64	0.13	0.01	1.14	(1.11 - 1.16)	6.0E-25	0.64	0.03	0.02	1.03	(1.00 - 1.06)	6.3E-02
rs12539172	chr7:100494172	NYAP1	C	T	0.70	0.08	0.01	1.09	(1.06 - 1.12)	9.3E-10	0.69	0.02	0.02	1.03	(0.99 - 1.06)	1.2E-01
rs9331896	chr8:27610169	CLU	T	C	0.61	0.13	0.01	1.14	(1.11 - 1.16)	4.6E-24	0.58	-0.02	0.02	0.98	(0.95 - 1.01)	1.3E-01
rs3740688	chr11:47358789	SPI1	T	G	0.55	0.08	0.01	1.09	(1.06 - 1.11)	5.4E-13	0.53	0.02	0.02	1.02	(0.99 - 1.05)	1.5E-01
rs6733839	chr2:127135234	LOC105373605	T	C	0.41	0.18	0.01	1.20	(1.17 - 1.23)	2.1E-44	0.40	0.02	0.02	1.02	(0.99 - 1.05)	1.8E-01
rs4844610	chr1:207629207	CR1	A	C	0.19	0.16	0.02	1.17	(1.14 - 1.21)	3.6E-24	0.19	0.02	0.02	1.02	(0.99 - 1.06)	2.3E-01
rs9271058	chr6:32607629	HLA-DRB1	A	T	0.27	0.10	0.01	1.10	(1.07 - 1.13)	1.4E-11	0.29	0.02	0.02	1.02	(0.98 - 1.06)	3.1E-01
rs593742	chr15:58753575	ADAM10	A	G	0.71	0.07	0.01	1.08	(1.05 - 1.10)	6.8E-09	0.66	0.02	0.02	1.02	(0.98 - 1.05)	3.3E-01
rs6024870	chr20:56422512	CASS4	G	A	0.91	0.13	0.02	1.14	(1.09 - 1.19)	3.5E-08	0.93	-0.02	0.03	0.98	(0.92 - 1.04)	4.2E-01
rs73223431	chr8:27362470	PTK2B	T	C	0.37	0.10	0.01	1.10	(1.07 - 1.13)	6.3E-14	0.37	0.01	0.02	1.01	(0.98 - 1.04)	4.7E-01
rs11218343	chr11:121564878	SORL1	T	C	0.96	0.22	0.03	1.25	(1.17 - 1.33)	2.9E-12	0.97	0.03	0.04	1.03	(0.95 - 1.11)	4.8E-01
rs12881735	chr14:92466484	SLC24A4	T	C	0.78	0.08	0.01	1.09	(1.06 - 1.12)	7.4E-09	0.78	0.01	0.02	1.01	(0.98 - 1.05)	5.5E-01
rs3752246	chr19:1056493	ABCA7	G	C	0.18	0.14	0.02	1.15	(1.11 - 1.19)	3.1E-16	0.16	0.01	0.02	1.01	(0.97 - 1.05)	5.6E-01
rs2830500	chr21:26784537	ADAMTS1	C	A	0.69	0.07	0.01	1.08	(1.05 - 1.10)	2.6E-08	0.70	0.01	0.02	1.01	(0.98 - 1.04)	5.7E-01
rs7920721	chr10:11678309	ECHDC3	G	A	0.39	0.08	0.01	1.08	(1.05 - 1.11)	2.3E-09	0.38	-0.01	0.02	0.99	(0.96 - 1.02)	6.5E-01
rs7185636	chr16:19796841	IQCK	T	C	0.82	0.08	0.01	1.09	(1.06 - 1.12)	2.4E-08	0.83	-0.01	0.02	0.99	(0.95 - 1.04)	6.9E-01
rs10808026	chr7:143402040	EPHA1	C	A	0.80	0.11	0.02	1.11	(1.08 - 1.15)	1.3E-10	0.80	0.01	0.02	1.01	(0.97 - 1.04)	7.3E-01
rs62039712	chr16:79321960	MAF	A	G	0.12	0.15	0.03	1.16	(1.10 - 1.22)	3.7E-08	0.12	0.01	0.02	1.01	(0.96 - 1.06)	7.3E-01
rs17125924	chr14:52924962	FERMT2	G	A	0.09	0.13	0.02	1.14	(1.09 - 1.19)	1.4E-09	0.10	-0.01	0.02	0.99	(0.94 - 1.04)	7.4E-01
rs114812713	chr6:41066261	OARD1	C	G	0.03	0.28	0.04	1.32	(1.23 - 1.42)	2.1E-13	0.03	-0.01	0.06	0.99	(0.88 - 1.10)	7.9E-01
rs138190086	chr17:63460787	CYB561	A	G	0.02	0.28	0.05	1.32	(1.20 - 1.45)	7.5E-09	0.02	0.01	0.06	1.01	(0.90 - 1.13)	9.1E-01
rs10933431	chr2:233117202	INPP5D	C	G	0.78	0.09	0.02	1.10	(1.07 - 1.13)	3.4E-09	0.78	0.00	0.02	1.00	(0.96 - 1.03)	9.5E-01
rs7933202	chr11:60169453	MS4A6A	A	C	0.61	0.12	0.01	1.12	(1.10 - 1.15)	1.9E-19	0.64	0.00	0.02	1.00	(0.97 - 1.03)	9.6E-01

EA = effect allele, OA= other allele. Effect estimates for Alzheimer's disease are derived from Kunkle et al (PMID 30820047).

Table S10 Multivariable association for Alzheimer's disease and falls on risk of hip fractures. Related to Fig 1.

		Heterogeneity		Inverse variance weighted Fixed effects model			Inverse variance weighted Random effects model			MR-Egger Regression					Multivariable/Weighted Median		
										Intercept		Slope					
Risk factor	N SNPs	Cochran's Q	P	OR	95% CI	P	OR	95% CI	P	Beta	P	OR	95% CI	P	OR	95% CI	P
**Individual associations**																	
Alzheimer's disease	24	17.59	7.8E-01	1.07	(1.05 - 1.10)	2.8E-12	1.07	(1.05 - 1.10)	2.8E-12	0.00	6.5E-01	1.08	(1.05 - 1.11)	3.2E-09	1.08	(1.06 - 1.10)	2.8E-11
Falls	3	5.22	7.4E-02	1.01	(0.63 - 1.61)	9.8E-01	1.01	(0.47 - 2.14)	9.9E-01						1.24	(0.64 - 2.43)	5.2E-01
**Multivariable associations**																	
Alzheimer's disease	27	22.67	6.0E-01	1.07	(1.05 - 1.10)	5.7E-11	1.07	(1.05 - 1.10)	5.7E-11	0.00	4.4E-01	1.08	(1.05 - 1.10)	1.5E-09	1.07	(1.05 - 1.10)	4.8E-08
Falls				1.09	(0.72 - 1.64)	6.8E-01	1.09	(0.72 - 1.64)	6.8E-01			1.11	(0.74 - 1.68)	6.1E-01	1.28	(0.69 - 2.38)	4.3E-01

Genetic instrument for Alzheimer's disease were derived from Kunkle et al (PMID 30820047) while genetic instruments for falls were derived from Trajanaskova et al (PMID 32999390)
