# Supplementary material for: Assessment of the genetic and clinical determinants of hip fracture risk: Genome-wide association and Mendelian randomization study
Source: Cell Rep Med. 2022 Oct 18;3(10):100776. doi: 10.1016/j.xcrm.2022.100776 (PMC9589021; doi:10.1016/j.xcrm.2022.100776)
Supplement: Document S2. Article plus supplemental information [file mmc3.pdf]

Cell Reports
Medicine

Article

Assessment of the genetic and clinical determinants of hip fracture risk: Genome-wide association and Mendelian randomization study

Graphical abstract

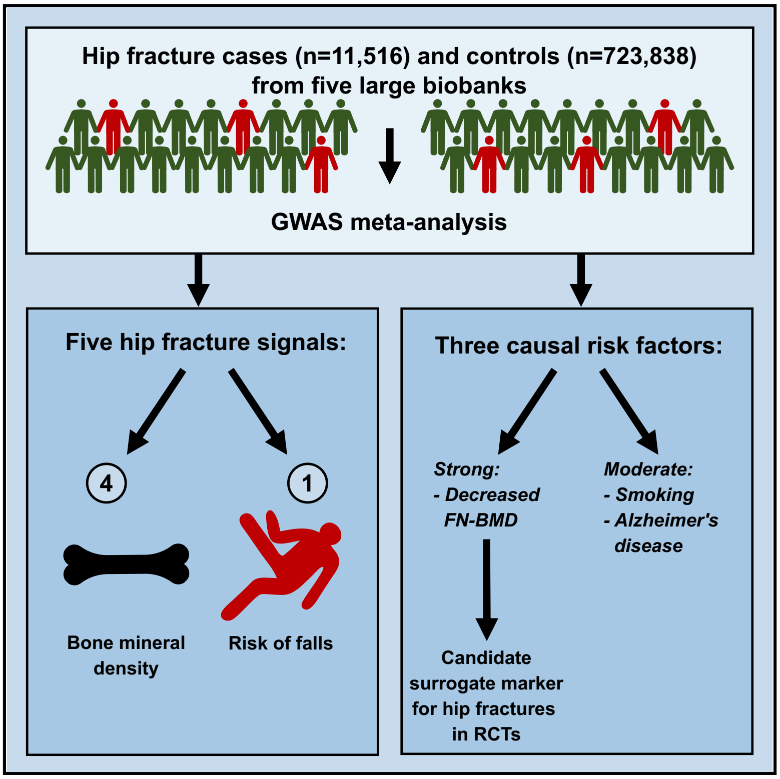

Highlights

- Five genetic signals associate with hip fractures in a meta-analysis of five cohorts
- Four of the signals associate also with BMD, while the last signal associates with falls
- Decreased femoral neck BMD is a strong causal risk factor for hip fractures
- Ever smoked regularly and Alzheimer's disease are moderate causal risk factors

Authors

Maria Nethander, Eivind Coward, Ene Reimann, ..., Ulrika Pettersson-Kymmer, Kristian Hveem, Claes Ohlsson

Correspondence

claes.ohlsson@medic.gu.se

In brief

Using five cohorts from European biobanks in a GWAS meta-analysis, Nethander et al. show that five genetic signals associate with hip fractures. For hip fractures, decreased femoral neck BMD is a strong causal risk factor, while ever smoked regularly and Alzheimer's disease are moderate causal risk factors.

Nethander et al., 2022, Cell Reports Medicine 3, 100776
October 18, 2022 © 2022 The Author(s).
https://doi.org/10.1016/j.xcrm.2022.100776

Cell Reports Medicine

CellPress
OPEN ACCESS

Article

Assessment of the genetic and clinical determinants of hip fracture risk: Genome-wide association and Mendelian randomization study

Maria Nethander,[1,2] Eivind Coward,[3] Ene Reimann,[4] Louise Grahnemo,[1] Maiken E. Gabrielsen,[3] Carl Wibom,[5] Estonian Biobank Research Team, Reedik Mägi,[4] Thomas Funck-Brentano,[1,6] Mari Hoff,[7,8] Arnulf Langhammer,[9,10] Ulrika Pettersson-Kymmer,[11] Kristian Hveem,[3,9,10] and Claes Ohlsson[1,12,13,*]

[1]Department of Internal Medicine and Clinical Nutrition, Institute of Medicine, Sahlgrenska Osteoporosis Centre, Centre for Bone and Arthritis Research at the Sahlgrenska Academy, University of Gothenburg, Vita Stråket 11, 41345 Gothenburg, Sweden
[2]Bioinformatics Core Facility, Sahlgrenska Academy, University of Gothenburg, Gothenburg, Sweden
[3]K.G. Jebsen Center for Genetic Epidemiology, Department of Public Health and Nursing, NTNU, Norwegian University of Science and Technology, 7491 Trondheim, Norway
[4]Estonian Genome Center, Institute of Genomics, University of Tartu, Riia 23b, 51010 Tartu, Estonia
[5]Department of Radiation Sciences, Oncology, Umea University, Umea, Sweden
[6]Department of Rheumatology, Lariboisière Hospital, INSERM U1132, Université de Paris, Paris, France
[7]Department of Neuromedicine and Movement Science, Norwegian University of Science and Technology, Trondheim, Norway
[8]Department of Rheumatology, St Olavs Hospital, Trondheim, Norway
[9]HUNT Research Centre, Forskningsveien 2, 7600 Levanger, Norway"
[10]Levanger Hospital, Nord-Trøndelag Hospital Trust, Levanger, Norway
[11]Department of Integrative Medical Biology, Clinical Pharmacology, Umea University, Umea, Sweden
[12]Region Västra Götaland, Department of Drug Treatment, Sahlgrenska University Hospital, Gothenburg, Sweden
[13]Lead contact
*Correspondence: claes.ohlsson@medic.gu.se
https://doi.org/10.1016/j.xcrm.2022.100776

SUMMARY

Hip fracture is the clinically most important fracture, but the genetic architecture of hip fracture is unclear. Here, we perform a large-scale hip fracture genome-wide association study meta-analysis and Mendelian randomization study using five cohorts from European biobanks. The results show that five genetic signals associate with hip fractures. Among these, one signal associates with falls, but not with bone mineral density (BMD), while four signals are in loci known to be involved in bone biology. Mendelian randomization analyses demonstrate a strong causal effect of decreased femoral neck BMD and moderate causal effects of Alzheimer's disease and having ever smoked regularly on risk of hip fractures. The substantial causal effect of decreased femoral neck BMD on hip fractures in both young and old subjects and in both men and women supports the use of change in femoral neck BMD as a surrogate outcome for hip fractures in clinical trials.

INTRODUCTION

Osteoporosis is a disease characterized by low bone mass and micro-architectural deterioration of bone tissue, leading to increased risk of fragility fractures.[1] However, fracture risk is not only determined by bone strength but also by the risk of falls that, in turn, is influenced by parameters such as muscle mass and function, balance, medications, and vision.[2,3] Hip fracture is the most severe type of fracture, associated with a high morbidity and mortality as well as high costs for society.[4,5] The hip fracture incidence increases exponentially by age, and by 2050, the worldwide annual number of hip fractures is expected to reach 4.5 to 6.3 million, reflecting the continuous aging of the population.[4,6]

The extent to which modification of predictive clinical risk factors reduces hip fracture risk is unknown. A better understanding of causal mechanisms for hip fractures will enable prevention strategies and provide targets for effective lifestyle and pharmacological interventions. Understanding whether interventions aimed at clinical risk factors would reduce fracture risk is important, enabling clinicians to ensure that such risk factors are optimized in individuals at high risk of fracture. If the risk factors are not causal, then such optimization would not decrease fracture risk.[7] Due to very high costs for randomized controlled osteoporosis registration trials with the required fracture endpoints, no new osteoporosis drugs are currently being evaluated in clinical trials.[8] The validation of bone mineral density (BMD) change as a surrogate outcome for fracture could reduce the size and costs

© 2022 The Author(s).
This is an open access article under the CC BY license (http://creativecommons.org/licenses/by/4.0/).

for randomized controlled osteoporosis registration trials. The use of BMD change as a surrogate outcome for fractures is supported by a recent large meta-analysis revealing that treatment-related BMD changes are strongly associated with fracture reductions across randomized trials of osteoporosis therapies with differing mechanisms of action.[8] An alternative approach to estimate the usefulness of treatment-related response on femoral neck BMD as a surrogate outcome to specifically reduce hip fractures is to use Mendelian randomization (MR) and determine the causal effect estimates of genetically determined femoral neck BMD on hip fracture risk. However, this requires the availability of a well-powered genome-wide association study (GWAS) on hip fractures.

Twin studies have shown a heritability estimate of 48% for hip fractures.[9] The heritable component of hip fracture risk may depend on both BMD-related and non-BMD-related genetic determinants. Although the genetic architecture of BMD has recently been evaluated in detail, the genetic determinants of hip fracture risk are essentially unknown.[10–12] Two recent large-scale GWASs on fractures at any bone site, including a mixture of fractures at different bone sites and also including less-validated self-reported fractures, have identified 15 fracture loci.[7,11] All the identified fracture loci were also associated with BMD, supporting the notion that BMD is a shared major risk factor for fractures at different bone sites. Nevertheless, it is likely that the causal mechanisms at least partly differ by fracture location. For instance, vertebral fracture risk depends largely on trabecular BMD, while risk of falls is an important determinant of hip fracture risk.[13]

We, herein, hypothesized that bone-site specific fracture loci, also including non-BMD-related loci, exist for hip fractures. To evaluate this hypothesis, we established a large dataset of hip fracture cases using data from five large Northern European biobanks in which high-quality national registers had been used to identify hip fractures according to the International Classification of Diseases (ICD; ICD10 codes S72.0–S72.2 and ICD9 820) and performed a large-scale hip fracture GWAS meta-analysis, including age- and gender-stratified analyses. This dataset was also used in an MR setting to identify causal clinical risk factors for hip fractures.

RESULTS

Genetic loci associated with hip fractures

We included 11,516 hip fracture cases and 723,838 controls from the HUNT, UFO, UK Biobank, Estonian Biobank, and FinnGen biobanks. The associations for 9,457,767 variants (minor allele frequency [MAF] > 0.01, imputation quality > 0.3) with hip fractures were evaluated. No evidence of excessive genomic inflation (λ = 1.02, linkage disequilibrium [LD] score intercept = 0.999) was observed in the GWAS meta-analysis, suggesting that the results were not biased because of population stratification, genotyping artifacts, or cryptic family relationships (Figure S1).

Five genomic loci were at a genome-wide significant level associated with hip fracture risk (Tables 1 and S5; Figures S1–S3). One of these hip fracture signals (rs429358 at the *APOE* locus on chromosome 19q13.32, p = 3.8×10^{-11}) is identical to the main established amino acid-altering genetic signal for Alzheimer's disease (Table 1).[14] The C allele of this SNP, which is associated with increased risk of Alzheimer's disease (Table 1; Figure S3A), was associated with increased risk of hip fractures (odds ratio [OR] per C allele, 1.14; 95% confidence interval [CI] 1.10–1.19) and increased risk of falls (Table 1). In contrast, it was not significantly associated with the combined less-validated fracture trait fractures at any bone site or with any measures of BMD (Table 1). Age-stratified analyses revealed that this genetic signal was associated with hip fracture risk in old ($\geq$71.2 years, OR per C allele, 1.33; 95% CI 1.25–1.43) but not in young (<71.2 years, OR 1.03; 95% CI 0.97–1.10) subjects, while gender-stratified analyses revealed similar associations for men and women (Table 1).

The other four genomic loci associated with hip fractures (*REST* at chromosome 4q12, *HOXC8* at chromosome 12q13.13, *SALL1* at chromosome 16q12.1, and *ETS2* at chromosome 21q22.2; Figures S3B–S3E), were at, or near, loci previously shown to be associated with different BMD measures (Table 1).[10,11] Gender-stratified analyses of these BMD-related loci revealed that the association for the genetic signal at the *SALL1* locus was more pronounced in women than in men (p = 3.2×10^{-2} for differences between ORs, z test; Table 1). The alleles of the four BMD-related genetic signals that associated with increased hip fracture risk were all associated with decreased femoral neck BMD (*ETS2*, *SALL1*, and *HOXC8*) and/or decreased estimated BMD by ultrasound in the heel (*ETS2*, *SALL1*, and *REST*) but not with risk of falls or Alzheimer's disease (Table 1).

Genetic correlation with clinical risk factors

LD score regression (LDSR) was used to estimate the genetic correlation between hip fractures and different diseases and traits. We evaluated in total 17 different genetic correlations for fractures at any bone site, BMD measures, and clinical risk factors for hip fractures (Table 2).

Femoral neck BMD (FN-BMD) was strongly ($r_g = -0.89$), while lumbar spine BMD (LS-BMD; $r_g = -0.43$) and estimated BMD in the heel using ultrasound (eBMD; $r_g = -0.48$) were moderately, inversely genetically correlated with hip fractures (Table 2). Among the different other evaluated clinical risk factors, only falls passed Bonferroni correction ($p < 0.05/17 = 0.0029$) and were directly genetically correlated with hip fractures ($r_g = 0.35$, p = 2.1×10^{-7}). In addition, hand grip strength was indirectly, while ever versus never smoked and alcohol consumption were directly, nominally ($p < 0.05$) genetically correlated with hip fractures, but these correlations did not pass Bonferroni correction for multiple testing (Table 2). None of the other evaluated risk factors was significantly genetically correlated with hip fractures (Table 2).

MR

Two-sample MR was used to test the causal effect of 15 plausible risk factors on hip fractures (Tables 3 and S7; Figure 1), and these effects were also compared with the effects on fractures at any bone site. There was clear evidence of a strong causal effect of genetically decreased FN-BMD (OR per SD decrease 2.12; 95% CI 1.82–2.47, p = 3.7×10^{-22}) and a

Table 1. Associations for BMD-related and non-BMD-related genome-wide significant SNPs for hip fractures

	Non-BMD-related locus			BMD-related loci													
	APOE (19q13.32)			*ETS2 (21q22.2)*			*SALL1 (16q12.1)*			*REST (4q12)*			*HOXC8 (12q13.13)*				
	rs429358, (EA C, EAF 0.17)			rs11088458 (EA G, EAF 0.70)			rs62028332 (EA G, EAF 0.87)			rs35339719 (EA G, EAF 0.74)			rs4142680 (EA T, EAF 0.41)[a]			n	
Fractures	OR	95% CI	p	OR	95% CI	p	OR	95% CI	p	OR	95% CI	p	OR	95% CI	p	Cases	Controls
Hip fracture	1.14	(1.10–1.19)	3.8×10^{-11}	1.11	(1.07–1.14)	3.7×10^{-10}	1.15	(1.10–1.20)	1.4×10^{-9}	1.11	(1.07–1.14)	1.8×10^{-9}	1.12	(1.08–1.16)	2.2×10^{-9}	11,516	723,838
Old	1.33	(1.25–1.43)	3.1×10^{-17}	1.11	(1.05–1.18)	1.3×10^{-4}	1.18	(1.10–1.26)	6.5×10^{-6}	1.08	(1.02–1.14)	7.2×10^{-3}	1.09	(1.03–1.14)	1.8×10^{-3}	4,700	609,945
Young	1.03	(0.97–1.10)	3.0×10^{-1}	1.10	(1.05–1.16)	1.9×10^{-4}	1.15	(1.07–1.23)	5.6×10^{-5}	1.15	(1.09–1.21)	9.8×10^{-8}	1.14	(1.09–1.20)	1.6×10^{-7}	4,334	609,945
Men	1.14	(1.06–1.23)	3.9×10^{-4}	1.09	(1.03–1.16)	5.8×10^{-3}	1.08	(1.00–1.17)	5.8×10^{-2}	1.11	(1.04–1.17)	1.3×10^{-3}	1.12	(1.05–1.18)	2.5×10^{-4}	3,070	266,328
Women	1.17	(1.11–1.24)	3.8×10^{-8}	1.12	(1.07–1.17)	3.3×10^{-6}	1.21	(1.14–1.28)	1.6×10^{-9}	1.11	(1.06–1.17)	4.0×10^{-6}	1.12	(1.07–1.17)	7.6×10^{-7}	5,980	343,617
Fracture at any bone site	1.01	(0.99–1.02)	6.0×10^{-1}	1.05	(1.03–1.06)	4.5×10^{-11}	1.04	(1.03–1.06)	6.9×10^{-6}	1.02	(1.00–1.03)	8.9×10^{-3}	1.01	(0.99–1.02)	2.4×10^{-1}	53,184	373,611
Other binary outcomes																	
Falls	1.02	(1.01–1.03)	6.3×10^{-4}	1.01	(1.00–1.02)	8.5×10^{-2}	1.00	(0.98–1.01)	5.2×10^{-1}	1.00	(0.99–1.01)	8.3×10^{-1}	1.01	(1.00–1.02)	1.0×10^{-1}	89,076	362,103
Alzheimer's disease	3.33	(3.20–3.45)	1.2×10^{-881}	0.98	(0.95–1.01)	2.3×10^{-1}	0.99	(0.94–1.03)	5.4×10^{-1}	1.00	(0.97–1.03)	8.3×10^{-1}	1.02	(0.98–1.05)	3.3×10^{-1}	35,274	59,163
BMD-related traits	Beta	SE	p	Beta	SE	p	Beta	SE	p	Beta	SE	p	Beta	SE	p	n	
FN-BMD	−0.010	0.011	4.0×10^{-1}	−0.030	0.008	4.1×10^{-4}	−0.060	0.012	3.0×10^{-7}	0.007	0.008	4.1×10^{-1}	−0.04	0.012	9.8×10^{-4}	49,998	
LS-BMD	0.006	0.013	6.3×10^{-1}	−0.025	0.010	1.1×10^{-2}	−0.023	0.013	9.0×10^{-2}	0.016	0.010	1.1×10^{-1}	−0.057	0.012	6.0×10^{-6}	44,731	
eBMD	0.001	0.003	7.9×10^{-1}	−0.044	0.002	6.8×10^{-82}	−0.041	0.004	5.2×10^{-20}	−0.015	0.002	1.8×10^{-11}	−0.002	0.003	6.3×10^{-1} [b]	426,824	

Besides the data for the five identified genetic signals in the present hip fracture GWAS meta-analyses, look ups were performed in the following published GWASs: fracture at any bone site,[11] falls,[15] Alzheimer's disease,[14] eBMD,[11] and FN-BMD and LS-BMD.[12] Odds ratios (OR) for binary outcomes are given per effect allele. Betas for continuous BMD-related parameters are expressed as SD per effect allele. EA, effect allele; EAF, effect allele frequency; BMD, bone mineral density; FN, femoral neck; LS, lumbar spine; eBMD, estimated bone mineral density in the heel using ultrasound. To achieve effect estimates not confounded by a possible minor dilution by diaphyseal and distal femur fractures and lack of adjustment for height and weight in the publicly available analyses of the FinnGen cohort, we replicated the five genome wide signals for hip fracture in a meta-analyses excluding the FinnGen cohort, yielding similar effect estimates (meta-analyses results excluding FinnGen; APOE, rs429358 OR 1.16 95% CI 1.11–1.21, $p = 2.5 \times 10^{-10}$; *ETS2*, rs11088458 OR 1.11 95% CI 1.07–1.15, $p = 7.4 \times 10^{-8}$; in the *ETS2* locus, the correlated SNP rs8130983 [$r^2 = 0.73$] was genome-wide significant in analyses without FinnGen OR 1.12 95% CI 1.08–1.16, $p = 1.1 \times 10^{-8}$; SALL1, rs62028332 OR 1.16 95% CI 1.10–1.22, $p = 3.1 \times 10^{-9}$; *REST*, rs35339719 OR 1.11 95% CI 1.07–1.15, $p = 3.4 \times 10^{-8}$; *HOXC8*, rs4142680 OR 1.12 95% CI 1.08–1.16, $p = 2.2 \times 10^{-9}$).

[a]Information for rs4142680 and hip fractures was only available in HUNT, UK Biobank, and UFO (8,401 cases and 501,168 controls).

[b]Derived from Neale et al. (http://www.nealelab.is/uk-biobank/), n = 206,496 as this SNP was not present in Zheng et al.[12]

Table 2. Estimated genetic correlation between hip fractures and risk factors for hip fractures

	Genetic correlation		
Disease or trait	r_g	SE	p
Fracture at any bone site	0.68[a]	0.10[a]	4.6×10^{-11} [a]
BMD-related measures			
FN-BMD[b]	−0.89[a]	0.10[a]	5.9×10^{-20} [a]
LS-BMD[b]	−0.43[a]	0.09[a]	1.2×10^{-6} [a]
eBMD	−0.48[a]	0.06[a]	5.7×10^{-16} [a]
Clinical risk factors			
Age at menopause[b]	−0.06	0.07	4.3×10^{-1}
Only females[b]	−0.01	0.11	9.1×10^{-1}
Age at menarche[b]	−0.01	0.05	8.1×10^{-1}
Only females[b]	−0.05	0.07	4.7×10^{-1}
Relative age voice broke[b]	0.01	0.07	9.0×10^{-1}
Only males[b]	−0.20	0.13	1.3×10^{-1}
Grip strength	−0.12	0.05	7.8×10^{-3}
Vitamin D levels	−0.01	0.06	8.9×10^{-1}
Falls	0.35[b]	0.07[b]	2.1×10^{-7} [a]
Coronary artery disease[b]	−0.04	0.06	5.4×10^{-1}
Rheumatoid arthritis[b]	0.06	0.07	4.4×10^{-1}
Inflammatory bowel disease[b]	0.06	0.07	3.8×10^{-1}
Type 2 diabetes[b]	−0.07	0.10	4.5×10^{-1}
Ever vs never smoked[b]	0.23	0.08	3.9×10^{-3}
Alcohol consumption	0.13	0.05	6.9×10^{-3}
Alzheimer's disease	0.08	0.11	4.7×10^{-1}

We evaluated the genetic correlation for plausible risk factors and Alzheimer's disease with hip fractures. The genetic correlations were evaluated either using LDhub or locally using public available GWAS summary statistics (fractures at any bone site,[7] eBMD,[11] grip strength,[16] falls,[15] vitamin D,[17] alcohol consumption,[18] Alzheimer's disease[14]). For diseases/traits including UK-Biobank in the GWAS and displaying significant genetic correlations ($p < 0.05/17 = 0.0029$) with hip fractures, sensitivity analyses were performed excluding UK Biobank in the hip fracture meta-analysis used for the correlations (analyses excluding UK Biobank: eBMD $r_g = -0.53$, $p = 5.1 \times 10^{-10}$; falls $r_g = 0.33$, $p = 2 \times 10^{-4}$). BMD, bone mineral density; FN, femoral neck; LS, lumbar spine; eBMD, estimated bone mineral density in the heel using ultrasound.
[a]Significant genetic correlation with risk of hip fracture passing Bonferroni adjusted level ($p<0.05/17= 0.0029$).
[b]Genetic correlations evaluated in the LD hub.

moderate causal effect of genetically decreased LS-BMD (OR per SD decrease 1.66; 95% CI 1.38–2.00, $p = 9.9 \times 10^{-8}$) and eBMD (OR per SD decrease 1.73; 95% CI 1.59–1.87, $p = 1.1 \times 10^{-39}$; Figure 1; Table 3) on hip fracture risk. Compared with the causal effect on fractures at any bone site, the causal effect on hip fractures was significantly more pronounced for genetically decreased FN-BMD ($p = 6.0 \times 10^{-3}$ using z test) but not for genetically decreased LS-BMD or eBMD (Figure 1; Table 3). Stratified analyses revealed that the magnitude of the causal effect of genetically decreased FN-BMD on hip fracture risk was similar in young (<71.2 years) and old (≥71.2 years) subjects, while it was slightly more pronounced in men (OR per SD decrease 2.80; 95% CI 2.27–3.45, $p = 7.2 \times 10^{-22}$) compared with women (OR per SD decrease 2.02; 95% CI 1.67–2.44, $p = 2.1 \times 10^{-13}$; $p = 2.3 \times 10^{-2}$ men versus women using z test; Figure 2A). When the individual genome-wide significant top SNPs in the different loci from an FN-BMD GWAS[10] were evaluated in a candidate approach, we observed that the lead SNP in as many as 25 of 47 loci was significantly ($p < 0.05$) associated with hip fracture risk in the expected direction (the allele associated with increased FN-BMD was associated with reduced hip fracture risk; Table S8). These data support the MR finding of a strong causal role of low FN-BMD on increased hip fracture risk.

In addition, genetically determined Alzheimer's disease was causally associated with increased risk of hip fractures (Figure 1; Table 3), and a sensitivity study excluding the genome-wide significant hip fracture SNP rs429358 at the *APOE* locus revealed a similar causal association (Figure S4). When evaluating the individual genome-wide significant top SNPs in the different loci from the used Alzheimer's disease GWAS,[14] we observed that, besides the top SNP in the *APOE* locus, the top SNP in the *CD2AP* locus on chromosome 6 was significantly associated with risk of hip fractures (Table S9). In addition, rs7412 in the *APOE* locus was modestly associated with hip fractures (OR 1.06, 95% CI 1.01–1.12 per C allele, $p = 0.03$). Stratified analyses demonstrated that the causal effect of genetically determined Alzheimer's disease on hip fractures was robust in old (≥71.2 years), but not in young (<71.2 years), subjects (Figure 2B). The causal effect of genetically determined Alzheimer's disease was of similar magnitude in men and women (Figure 2B).

Ever smoked regularly was also causally associated with increased risk of hip fractures and fractures at any bone site (Figure 1; Table 3). MR demonstrated that this smoking parameter was causally related to risk of falls (summary statistics for falls from Trajanoska et al.;[15] OR: 1.08, 95% CI: 1.06–1.10, $p = 7.7 \times 10^{-16}$) but not FN-BMD (summary statistics for FN-BMD from Zheng et al;.[12] beta = −0.009, SE = 0.014; $p = 0.51$).

Some evidence was observed that late puberty was causally associated with increased hip fracture risk in females, but this association did not pass Bonferroni correction. None of the other 9 evaluated risk factors for fractures showed evidence of a causal effect on risk of hip fractures, despite often adequate statistical power (Figure 1; Tables 3 and S6). For all of the reported significant causal associations, the estimates from the inverse variance weighted fixed effects meta-analysis were very similar to the estimates from the weighted median and penalized weighted median methods (Table S7).

To exclude reverse causality, wherein hip fractures influence risk of Alzheimer's disease, we performed a bidirectional MR using hip fracture (GWAS SNPs from the present hip fracture GWAS, excluding the established strong Alzheimer's SNP rs429358) as the exposure and risk of Alzheimer's disease[14] as the outcome, revealing no evidence that genetically determined hip fracture was causally related to Alzheimer's disease (OR per doubling of diseases susceptibility 0.96; 95% CI 0.82–1.12, $p = 0.63$).

As hip fracture risk is associated with increased risk of falls and reduced FN-BMD in observational studies, we determined the effects of genetically determined Alzheimer's disease on risk of falls and FN-BMD. We observed that genetically determined Alzheimer's disease (GWAS SNPs from Kunkle at al.[14]) was causally related to increased risk of falls (GWAS summary statistics from

Table 3. Mendelian randomization to estimate the causal effects of 15 genetically determined risk factors on hip fractures

	Inverse variance weighted meta-analyses									Egger regression	
							Power			Intercept, p	
	Hip fractures			Any fractures			Hip		Any		
Trait or disease	OR	95% CI	p	OR	95% CI	p	OR 1.15, %	OR 1.20, %	OR 1.15, %	Hip fractures	Any fractures
**Continuous risk factors**											
**BMD-related parameters**											
Decreased FN-BMD	2.12	(1.82–2.47)	3.7×10^{-22} [a]	1.63	(1.44–1.84)	3.3×10^{-15} [a]	96	100	100	1.2×10^{-1}	4.4×10^{-1}
Decreased LS-BMD	1.66	(1.38–2.00)	9.9×10^{-8} [a]	1.56	(1.39–1.76)	7.2×10^{-14} [a]	97	100	100	5.5×10^{-4} [a]	4.1×10^{-1}
Decreased eBMD	1.73	(1.59–1.87)	1.1×10^{-39} [a]	1.65	(1.59–1.71)	3.0×10^{-151} [a]	100	100	100	6.4×10^{-2}	8.5×10^{-1}
**Other risk markers**											
Early menopause	0.95	(0.87–1.04)	3.0×10^{-1}	1.07	(1.03–1.12)	9.0×10^{-4} [a]	96	100	100	2.3×10^{-1}	8.8×10^{-1}
Only females	1.01	(0.88–1.16)	8.5×10^{-1}	N/A	N/A	N/A	75	95	N/A	9.6×10^{-2}	N/A
Late puberty	1.13	(1.01–1.27)	3.3×10^{-2}	1.07	(1.01–1.13)	2.5×10^{-2}	69	91	99	3.2×10^{-2}	8.5×10^{-1}
Only females	1.18	(1.02–1.38)	2.8×10^{-2}	N/A	N/A	N/A	40	65	N/A	2.5×10^{-1}	N/A
Only males	1.02	(0.83–1.24)	8.7×10^{-1}	N/A	N/A	N/A	22	37	N/A	4.1×10^{-3}	N/A
Decreased TSH	1.01	(0.91–1.11)	8.6×10^{-1}	0.99	(0.95–1.03)	6.2×10^{-1}	95	100	100	4.8×10^{-1}	8.1×10^{-1}
Decreased grip strength/BW	1.06	(0.87–1.30)	5.6×10^{-1}	1.21	(1.09–1.34)	3.4×10^{-4} [a]	48	74	91	3.8×10^{-1}	2.4×10^{-1}
Low vitamin D levels	0.98	(0.87–1.10)	7.1×10^{-1}	0.99	(0.94–1.04)	5.9×10^{-1}	99	100	100	3.8×10^{-1}	1.8×10^{-1}
**Binary risk factors**											
Alzheimer's disease	1.07	(1.05–1.10)	1.9×10^{-12} [a]	1.00	(0.99–1.01)	6.5×10^{-1}	100	100	100	7.2×10^{-1}	7.6×10^{-1}
Coronary heart disease	1.01	(0.95–1.08)	6.7×10^{-1}	1.01	(0.99–1.03)	4.3×10^{-1}	95	100	100	4.0×10^{-1}	2.6×10^{-1}
Rheumatoid arthritis	1.00	(0.97–1.02)	9.3×10^{-1}	1.01	(1.00–1.02)	1.0×10^{-1}	95	100	100	6.3×10^{-1}	4.3×10^{-1}
Inflammatory bowel disease	1.01	(1.00–1.03)	1.2×10^{-1}	1.00	(0.99–1.01)	7.8×10^{-1}	99	100	100	3.6×10^{-1}	8.9×10^{-1}
Type 1 diabetes	1.00	(0.98–1.02)	8.6×10^{-1}	1.00	(0.99–1.00)	6.6×10^{-1}	98	100	100	5.5×10^{-1}	8.4×10^{-1}
Type 2 diabetes	1.03	(0.99–1.06)	1.8×10^{-1}	1.00	(0.98–1.03)	6.3×10^{-1}	95	100	100	2.2×10^{-1}	7.6×10^{-2}
Ever smoked regularly	1.08	(1.03–1.13)	2.3×10^{-3} [a]	1.05	(1.03–1.07)	1.4×10^{-4} [a]	61	96	97	7.6×10^{-1}	2.3×10^{-1}

Inverse variance weighted meta-analysis. Estimates for the association with hip fracture are from the present hip fracture GWAS, while the estimates from fractures at any bone site are from the summary statistics in Morris et al.[11] Odds ratio (OR) is for the risk of fracture per standard deviation (SD) change in the risk factor for continuous trait or risk of fracture per doubling of odds (obtained by multiplying the causal estimate of log odds by $\ln(2) \approx 0.69$)[19] of disease susceptibility for binary factors. For menopause and puberty, we used the estimated SD from the largest cohorts in the published GWAS (early menopause SD = 3.81 years in Breast Cancer Association Consortium;[20] late puberty SD = 1.40 years in Women's Genome Health Study[21]) to translate the effect unit from year to SD. For ever smoked regularly, the ORs are expressed per 0.5 unit increase in log odds of ever smoking regularly with a 1 SD increase in genetically predicted smoking initiation corresponding to a 10% increased risk of smoking.[18,22] Estimates are displayed using a random effects model to account for possible heterogeneity. Statistical power is given to detect an odds ratio of 1.15 or 1.20 at $\alpha \leq 3.3 \times 10^{-3}$ (0.05/15 risk factors). Egger intercepts are given in this table, while Egger effect estimates are presented in Table S7.For risk factors including UK Biobank in the GWAS and displaying significant causal effects with hip fractures, sensitivity analyses were performed excluding UK Biobank in the hip fracture meta-analysis used for the mendelian randomization, revealing essentially similar effect estimates (results excluding UK Biobank in the hip fracture GWAS; decreased eBMD OR = 1.66, 95% CI: 1.54–1.79; ever smoked regularly OR = 1.07, 95% CI: 1.01–1.14). Grip strength is given as grip strength per body weight (BW). SD for grip strength is given for kg grip strength per kg in BW and was estimated in the UK Biobank to be 0.127. To achieve effect estimates not confounded by a possible minor dilution by diaphyseal and distal femur fractures and lack of adjustment for height and weight in the publicly available analyses of the FinnGen cohort, we replicated the significant causal associations for FN-BMD (OR 2.25, 95% CI 1.91–2.65), Alzheimer's disease (OR 1.08, 95% CI 1.06–1.11), and ever smoked regularly (OR 1.06, 95% CI 1.00–1.12) in a meta-analysis excluding the FinnGen cohort, yielding similar effect estimates. N/A, not available; BMD, bone mineral density; TSH, thyroid-stimulating hormone; FN, femoral neck; LS, lumbar spine; eBMD, estimated BMD in the heel using ultrasound; CI, confidence interval.

[a]Findings that remain significantly associated after correction for multiple testing ($p < 0.05/15 = 3.3 \times 10^{-3}$).

CellPress
OPEN ACCESS

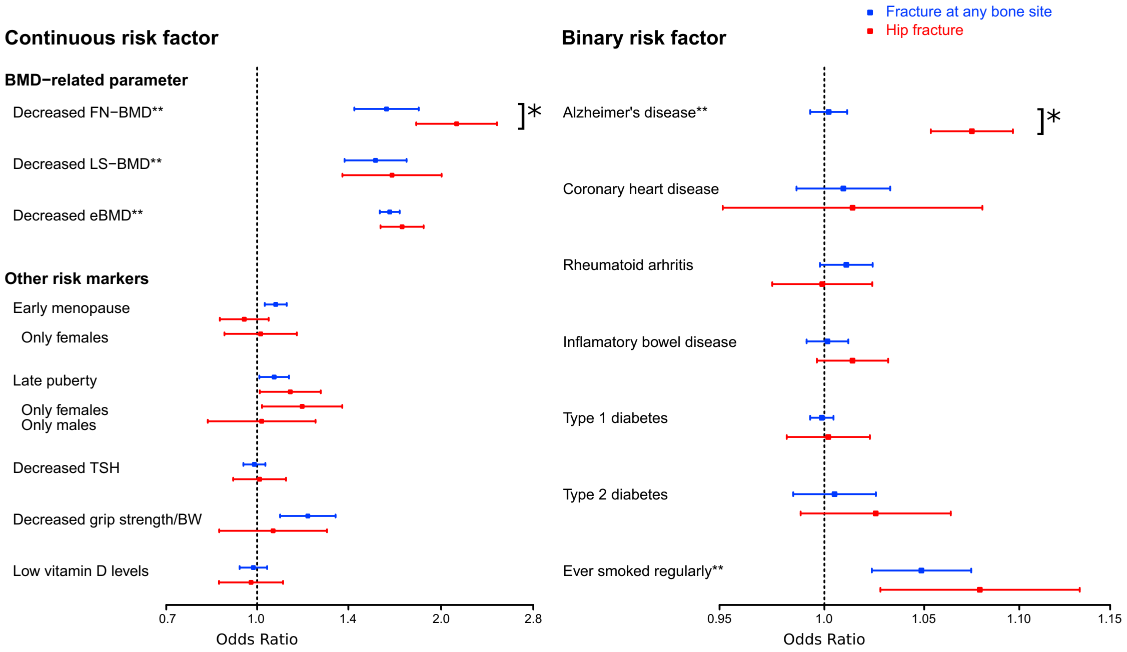

Figure 1. Mendelian randomization to estimate the causal effects of 15 genetically determined risk factors on risks of hip fractures (red) and fractures at any bone site (blue)

Estimates for the association with hip fracture is from the present hip fracture GWAS, while the estimates from any fracture are from the summary statistics in Morris et al.[11] Inverse variance weighted meta-analyses were performed. Odds ratio (OR) and 95% confidence intervals are for the risk of fractures per standard deviation change in the risk factor for continuous trait or risk of fracture per doubling of odds (obtained by multiplying the causal estimate of log odds by ln(2) ≈ 0.69)[19] of disease susceptibility for binary factors. For ever smoked regularly, the ORs are expressed per 0.5 unit increase in log odds of ever smoking regularly with a 1 standard deviation increase in genetically predicted smoking initiation corresponding to a 10% increased risk of smoking.[18,22] Estimates are displayed using a random effects model to account for possible heterogeneity. *, the ORs differ significantly between hip fracture and fractures at any bone site as determined by a z test. **, risk factors that remain significantly causally associated with hip fractures after correction for multiple testing ($p < 0.05/15 = 3.3 \times 10^{-3}$). For risk factors including UK Biobank in the GWAS and displaying significant causal effects with hip fractures, sensitivity analyses were performed excluding UK Biobank in the hip fracture meta-analysis used for the Mendelian randomization, revealing essentially similar effect estimates (results excluding UK Biobank in the hip GWAS; decreased eBMD OR = 1.66; 95% confidence interval [CI]: 1.54–1.79; ever smoked regularly OR = 1.07; 95% CI: 1.01–1.14). Grip strength is given as grip strength per body weight. Standard deviation (SD) for grip strength is given for kg grip strength per kg in body weight and was estimated in the UK Biobank to be 0.127. To achieve effect estimates not confounded by a possible minor dilution by diaphyseal and distal femur fractures among hip fractures and lack of adjustment for height and weight in the publicly available analysis of the FinnGen cohort, we replicated the significant causal associations for FN-BMD (OR 2.25; 95% CI 1.91–2.65), Alzheimer's disease (OR 1.08; 95% CI 1.06–1.11), and ever smoked regularly (OR 1.06; 95% CI 1.00–1.12) in a meta-analysis excluding the FinnGen cohort, yielding similar effect estimates. BMD, bone mineral density; TSH, thyroid-stimulating hormone; FN, femoral neck; LS, lumbar spine; eBMD, estimated BMD in the heel using ultrasound.

Trajanoska et al.;[15] OR per doubling of diseases susceptibility 1.02; 95% CI 1.01–1.03; $p = 6.3 \times 10^{-3}$) but not to FN-BMD (summary statistics from Zheng et al.;[12] SD per doubling of diseases susceptibility beta = 0.015, SE = 0.012; p = 0.21). Therefore, it is biologically plausible that Alzheimer's disease increases hip fracture risk at least partly via increased risk of falls without altering FN-BMD. A multivariable analysis using both Alzheimer's disease and falls as exposures did not support a strong mediating effect of falls for the causal association between Alzheimer's disease and hip fractures (Table S10). However, it should be noted that the genome-wide significant SNPs derived from the falls GWAS explained only 0.28% of the variation in falls,[15] suggesting limited power in this multivariable analysis. In addition, as Alzheimer's disease results in reduced memory, it is possible that patients with Alzheimer's disease underreport falls.

The three MR assumptions were evaluated as previously described.[7] We only selected variants with a MAF > 1% that were strongly associated with the clinical risk factor ($p < 5 \times 10^{-8}$), ensuring that the genetic variants used as instrumental variables are associated with the clinical risk factor (first assumption). The impact of reported associations between the genetic variants and potential confounding factors (second assumption) was searched for in the literature and in the GWAS catalog (https://www.ebi.ac.uk/gwas/). We observed that the genetic signal for both hip fractures (the present study) and Alzheimer's disease, rs429358, was also robustly associated with C-reactive protein levels and serum cholesterol levels. However, using MR and adequate genetic instruments (Table S11), we did not observe any evidence of a causal effect of C-reactive protein (CRP; genetic instruments from Han et al.[23]) or low-density lipoprotein (LDL) cholesterol or high-density lipoprotein (HDL) cholesterol (genetic instruments for both HDL and LDL cholesterol from Willer et al.[24]) on hip fractures (inverse variance weighted MR; CRP, OR 0.95 (0.88–1.02), p = 0.14; LDL cholesterol OR 1.01 (0.92–1.12), p = 0.80; HDL cholesterol OR

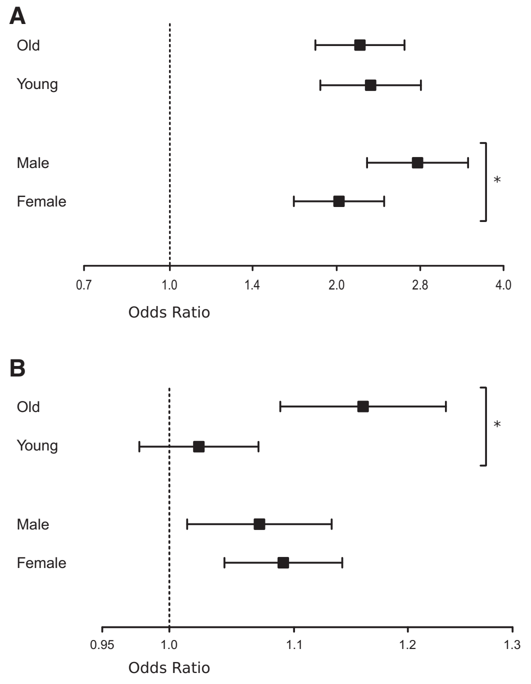

Figure 2. Age- and gender- stratified Mendelian randomization analyses

Age- and gender-stratified Mendelian randomization to estimate the causal effects of (A) decreased FN-BMD and (B) Alzheimer's disease on hip fracture risk. Inverse variance weighted meta-analysis were performed. OR and 95% CIs are for the risk of hip fractures per SD decrease in genetically determined FN-BMD or per doubling of odds of Alzheimer's disease. Estimates are displayed using a random effects model to account for possible heterogeneity. Age-stratified analyses are divided by the median age (71.2 years) of the hip fracture cases. *, the ORs differ as determined by a z test ($p < 0.05$).

1.03 (0.93–1.14) p = 0.56; ORs are expressed per SD increase in exposure). Finally, for the reported significant causal associations, no evidence of horizontal pleiotropy between the instruments and the outcomes was observed, except for LS-BMD, using MR Egger regression (Tables 3 and S7).

DISCUSSION

Although hip fracture is the clinically most important fracture, the genetic architecture and the causal risk factors of hip fractures are not characterized in detail. We, herein, established a large hip fracture dataset and performed a large-scale GWAS meta-analysis on hip fractures, identifying one non-BMD-related genetic hip fracture signal and four signals in loci known to be involved in bone biology. In addition, we demonstrate that the causal clinical risk factors for hip fractures include decreased FN-BMD, Alzheimer's disease, and ever smoked regularly. We also demonstrate that the impact of these causal risk factors differs by age, gender, and fracture site. Finally, we provide causal effect estimates of the effect of FN-BMD on hip fractures, including separate effect estimates by age and gender, supporting the use of change in FN-BMD as an efficient surrogate outcome to estimate effects of BMD-targeting treatments on hip fractures in future clinical trials.

Interestingly, in the present GWAS on hip fracture, one of the identified genetic signals, located in the *APOE* locus, is associated with falls but not associated with any BMD measures. Falls are a major determinant of hip fractures,[2–4,13] and the allele of the *APOE* signal associated with increased hip fracture risk was also associated with increased risk of falls, suggesting that this signal has an effect on hip fracture risk via risk of falls and not via bone strength. In addition, it is possible that this signal also may influence the pattern of falls as decline in age-related neuromuscular function is reported to change how we fall. When not appropriately stretching out the arms, we more often fall on the side, directly on the hip.[25] Age-stratified analyses revealed that this genetic signal was associated with hip fracture risk in old, but not in young, subjects. The hip fracture signal identified at the *APOE* locus is identical to the main established amino acid-altering genetic signal for Alzheimer's disease (in exon 4 of Apo lipoprotein E).[14,26] The allele that increases the risk of Alzheimer's disease also increases the risk of hip fractures and falls.

Our MR analyses demonstrated that Alzheimer's disease is causally associated with increased hip fracture risk, and a sensitivity study excluding the genome-wide significant hip fracture signal at the *APOE* locus revealed a similar causal association. In support of a mechanism of Alzheimer's disease on hip fracture risk involving increased risk of falls, MR demonstrated that Alzheimer's disease was causally directly related to falls but not to FN-BMD. Furthermore, genetic correlation analyses revealed that falls were significantly directly correlated with hip fractures, suggesting that the genetic architecture of falls and hip fractures are partly overlapping. Previous observational studies have reported that Alzheimer's disease is associated with increased number and severity of falls.[13,26]

The signals in the other four genomic loci associated with hip fractures (*REST*, *HOXC8*, *SALL1*, and *ETS2*) were previously shown to associate with different BMD measures,[10,11] supporting the notion that BMD is an important determinant also of hip fracture risk. None of these four BMD-related signals were associated with risk of falls, suggesting that these signals mainly affected hip fracture risk via reduced bone strength. The signal in the *ETS2* locus has previously been identified in a GWAS on fractures at any bone site, while the signals at the *SALL1* and *REST* loci were nominally ($p < 0.05$) significantly associated with fractures at any bone site in the expected direction.[7,11] A highly correlated SNP to the top SNP in the *HOXC8* locus was identified in a GWAS on LS bone area to also be associated with fractures.[27] Thus, all these four identified BMD-related hip fracture signals are known to be involved in bone biology.

A validated surrogate outcome for fracture would reduce the size, duration, and cost of trials of new osteoporosis treatments, thereby facilitating drug development. Thus, there is a clinical need of an FDA-approved surrogate marker for fractures as outcomes in clinical osteoporosis trials.[8] Two-sample MR, using strong genetic instruments, is a methodology to obtain precise causal effect estimates of FN-BMD on hip fractures that is not affected by confounders or reverse causality. Using the present large-scale GWAS on hip fractures, we demonstrated a strong causal association of decreased FN-BMD on increased hip fracture risk. Our further stratified analyses yielded separate effect estimates by age and gender, revealing that FN-BMD exerts a

substantial causal effect on hip fracture risk in both men and women and in both young and old subjects, supporting the use of change in FN-BMD as a general surrogate outcome in randomized clinical trials that estimate the effects of BMD-targeting treatments on hip fractures.

In addition to decreased FN-BMD and Alzheimer's disease, ever smoked regularly was causally associated with increased risk of hip fracture. Ever smoked regularly was chosen as a measure of smoking, as strong genetic instruments were available for this trait.[18] As there was some overlap between the populations included in the GWAS for ever smoked regularly and the present GWAS on hip fractures, this causal association should be confirmed in additional cohorts. Interestingly, this smoking parameter was directly causally associated with risk of falls but not associated with FN-BMD. In addition to increasing risk of falls, it is possible that smoking may regulate bone strength via effects on bone microstructure or other bone quality parameters, which are not captured by FN-BMD. A causal effect of smoking on hip fracture risk is supported by some previous observational association studies.[28,29]

The strength of this study is the large number of hip fracture cases included, generating the most comprehensive assessment of the genetic determinants of hip fracture risk so far. This well-powered dataset also enabled us to identify and estimate the strength of causal clinical risk factors for hip fractures.

In conclusion, this hip fracture GWAS identified one non-BMD-related and four BMD-related genetic determinants for hip fractures. MR analyses demonstrated a strong causal effect of decreased FN-BMD and moderate causal effects of Alzheimer's disease and ever smoked regularly on risk of hip fractures. The present study demonstrates that the genetic architecture of fractures is complex and at least partly differs by bone site, age, and gender and, for hip fractures, involves both BMD-related and non-BMD-related signals. The substantial causal effect of FN-BMD on hip fracture risk in both young and old subjects and in both men and women supports the use of change in FN-BMD as a general surrogate outcome in randomized clinical trials to estimate effects of BMD-targeting treatments on hip fractures.

Limitations of the study

Some limitations of the present study need to be considered. As the available genetic instruments for falls and alcohol consumption were very weak, the causal associations for these two relevant risk factors for hip fractures could not be evaluated. Moreover, as the hip fracture GWAS was adjusted for height and weight, we could not assess the possible causal associations of BMI or height on hip fractures. In addition, it is a major limitation with the present study that the analyses were restricted to participants of White ancestry. Therefore, additional analyses are necessary to investigate whether our results also apply to those of other ethnicities. As MR assumes a linear relation between the risk factor and the outcome, the present finding of no apparent causal association between low vitamin D and hip fractures does not exclude a non-linear threshold association between low vitamin D and hip fractures. Also, the present findings need to be replicated in independent cohorts.

STAR★METHODS

Detailed methods are provided in the online version of this paper and include the following:

- KEY RESOURCES TABLE
- RESOURCE AVAILABILITY
  - Lead contact
  - Materials availability
  - Data and code availability
- EXPERIMENTAL MODEL AND SUBJECT DETAILS
  - Study population
- METHOD DETAILS
  - Hip fracture definition
- QUANTIFICATION AND STATISTICAL ANALYSIS
  - Genome-wide association study and meta-analyses
  - Genetic correlation
  - Mendelian randomization
  - Power calculation

SUPPLEMENTAL INFORMATION

Supplemental information can be found online at https://doi.org/10.1016/j.xcrm.2022.100776.

CONSORTIA

The Estonian Biobank Research Team includes Mari Nelis, Lili Milani, Tõnu Esko, and Andres Metspalu.

ACKNOWLEDGMENTS

HUNT: The Trøndelag Health Study (HUNT) is a collaboration between HUNT Research Center (Faculty of Medicine and Health Sciences, Norwegian University of Science and Technology NTNU), Trøndelag County Council, Central Norway Regional Health, and the Norwegian Institute of Public Health. We thank the population of the County of Trøndelag and the staff at HUNT Research Center for their contribution.

UK Biobank: UK Biobank was established by the Wellcome Trust medical charity, Medical Research Council, Department of Health, the Scottish government, and the Northwest Regional Development Agency. It has also had funding from the Welsh government, British Heart Foundation, Cancer Research UK, and Diabetes UK. UK Biobank is supported by the National Health Service (NHS). UK Biobank is open to bona fide researchers anywhere in the world, including those funded by academia and industry. The medical research project is a non-profit charity that has received core funding of around £133 million. Core funding continues to be received from the Wellcome Trust, the MRC, and, more recently, Cancer Research UK and NIHR. UK Biobank has received additional funding for genotyping of all 500,000 participants (from the Department of Health, Medical Research Council, and British Heart Foundation). We want to acknowledge the participants and investigators of UK Biobank. This research has been conducted using the UK Biobank Resource under application number 51784.

UFO cohort: We thank Åsa Ågren, Hubert Sjödin, and Magnus Hellström for data processing and for help creating the UFO database and Kerstin Enquist and Ann-Marie Åhrén for their help in preparing samples for genotyping. We would also like to thank the participants and staff from the NSHDS cohort study and all collaborators in the UFO study group. UFO is supported by the Swedish Research Council (K20006-72X-20155013), the Swedish Sports Research Council (87/06), the Swedish Society of Medicine, and the Kempe Foundation (JCK-1021) and by grants from the Medical Faculty of Umeå University (ALFVLL:968:22-2005, ALFVLL: 937-2006, ALFVLL:223:11-2007, and ALFVLL:78151-2009) and from the county council of Västerbotten (Spjutspetsanslag VLL:159:33-2007).

Estonian Biobank: We acknowledge all the Estonian Biobank participants and the team. Data analyses were carried out in part in the High-Performance Computing Center of University of Tartu. The Estonian Biobank was funded by the European Union through the European Regional Development Fund (project nos. 2014-2020.4.01.15-0012, 2014-2020.4.01.16-0125, and MOBEC008), by the European Union through Horizon 2020 grant no. 810645, and by the Estonian Research Council grants PUT (PRG687 and PRG1291).

FinnGen: We want to acknowledge the participants and investigators of FinnGen study.

Funding to C.O. for the present analyses include grants from the Swedish Research Council, the Swedish state under the agreement between the Swedish government and the county councils, the ALF agreement, the Lundberg Foundation, the Torsten Söderberg Foundation, the Novo Nordisk Foundation, and the Knut and Alice Wallenberg Foundation.

AUTHOR CONTRIBUTIONS

The design of the study was performed in collaboration between M.N., E.C., A.L., K.H., T.F.-B., L.G., M.H., and C.O. A.L., M.H., M.E.G., C.W., U.P.-K., R.M., E.R., C.O., M.N., E.C., K.H., and U.P.-K. were either responsible for the different included cohorts or performed cohort-specific hip fracture GWASs. M.N. and C.O. performed the GWAS meta-analyses of summary statistics provided by the different contributing cohorts. M.N., T.F.-B., and C.O. performed two-sample MR analyses. C.O. wrote the first draft of the manuscript. All the authors contributed to subsequent drafts of the manuscript and made the decision to submit the manuscript for publication.

DECLARATION OF INTERESTS

The authors declare no competing interests.

Received: March 7, 2022
Revised: June 7, 2022
Accepted: September 19, 2022
Published: October 18, 2022

REFERENCES

1. Kanis, J.A., Melton, L.J., 3rd, Christiansen, C., Johnston, C.C., and Khaltaev, N. (1994). The diagnosis of osteoporosis. J. Bone Miner. Res. *9*, 1137–1141. https://doi.org/10.1002/jbmr.5650090802.
2. Harvey, N.C., Odén, A., Orwoll, E., Lapidus, J., Kwok, T., Karlsson, M.K., Rosengren, B.E., Ljunggren, Ö., Cooper, C., McCloskey, E., et al. (2018). Falls predict fractures independently of FRAX probability: a meta-analysis of the osteoporotic fractures in men (MrOS) study. J. Bone Miner. Res. *33*, 510–516. https://doi.org/10.1002/jbmr.3331.
3. Ohlsson, C. (2013). Bone metabolism in 2012: novel osteoporosis targets. Nat. Rev. Endocrinol. *9*, 72–74. https://doi.org/10.1038/nrendo.2012.252.
4. Ferrari, S., Reginster, J.Y., Brandi, M.L., Kanis, J.A., Devogelaer, J.P., Kaufman, J.M., Féron, J.M., Kurth, A., and Rizzoli, R. (2016). Unmet needs and current and future approaches for osteoporotic patients at high risk of hip fracture. Arch. Osteoporos. *11*, 37. https://doi.org/10.1007/s11657-016-0292-1.
5. Johnell, O., and Kanis, J.A. (2006). An estimate of the worldwide prevalence and disability associated with osteoporotic fractures. Osteoporos. Int. *17*, 1726–1733. https://doi.org/10.1007/s00198-006-0172-4.
6. Kanis, J.A., Odén, A., McCloskey, E.V., Johansson, H., Wahl, D.A., Cooper, C., and IOF Working Group on Epidemiology and Quality of Life; and Quality of, L. (2012). A systematic review of hip fracture incidence and probability of fracture worldwide. Osteoporos. Int. *23*, 2239–2256. https://doi.org/10.1007/s00198-012-1964-3.
7. Trajanoska, K., Morris, J.A., Oei, L., Zheng, H.F., Evans, D.M., Kiel, D.P., Ohlsson, C., Richards, J.B., Rivadeneira, F., and GEFOS/GENOMOS consortium and the 23andMe research team; and the 23andMe research, t. (2018). Assessment of the genetic and clinical determinants of fracture risk: genome wide association and mendelian randomisation study. BMJ *362*, k3225. https://doi.org/10.1136/bmj.k3225.
8. Black, D.M., Bauer, D.C., Vittinghoff, E., Lui, L.Y., Grauer, A., Marin, F., Khosla, S., de Papp, A., Mitlak, B., Cauley, J.A., et al. (2020). Treatment-related changes in bone mineral density as a surrogate biomarker for fracture risk reduction: meta-regression analyses of individual patient data from multiple randomised controlled trials. Lancet Diabetes Endocrinol. *8*, 672–682. https://doi.org/10.1016/S2213-8587(20)30159-5.
9. Michaëlsson, K., Melhus, H., Ferm, H., Ahlbom, A., and Pedersen, N.L. (2005). Genetic liability to fractures in the elderly. Arch. Intern. Med. *165*, 1825–1830. https://doi.org/10.1001/archinte.165.16.1825.
10. Estrada, K., Styrkarsdottir, U., Evangelou, E., Hsu, Y.H., Duncan, E.L., Ntzani, E.E., Oei, L., Albagha, O.M.E., Amin, N., Kemp, J.P., et al. (2012). Genome-wide meta-analysis identifies 56 bone mineral density loci and reveals 14 loci associated with risk of fracture. Nat. Genet. *44*, 491–501. https://doi.org/10.1038/ng.2249.
11. Morris, J.A., Kemp, J.P., Youlten, S.E., Laurent, L., Logan, J.G., Chai, R.C., Vulpescu, N.A., Forgetta, V., Kleinman, A., Mohanty, S.T., et al. (2019). An atlas of genetic influences on osteoporosis in humans and mice. Nat. Genet. *51*, 258–266. https://doi.org/10.1038/s41588-018-0302-x.
12. Zheng, H.F., Forgetta, V., Hsu, Y.H., Estrada, K., Rosello-Diez, A., Leo, P.J., Dahia, C.L., Park-Min, K.H., Tobias, J.H., Kooperberg, C., et al. (2015). Whole-genome sequencing identifies EN1 as a determinant of bone density and fracture. Nature *526*, 112–117. https://doi.org/10.1038/nature14878.
13. Cummings, S.R., and Eastell, R. (2016). Risk and prevention of fracture in patients with major medical illnesses: a mini-review. J. Bone Miner. Res. *31*, 2069–2072. https://doi.org/10.1002/jbmr.3030.
14. Kunkle, B.W., Grenier-Boley, B., Sims, R., Bis, J.C., Damotte, V., Naj, A.C., Boland, A., Vronskaya, M., van der Lee, S.J., Amlie-Wolf, A., et al. (2019). Genetic meta-analysis of diagnosed Alzheimer's disease identifies new risk loci and implicates Abeta, tau, immunity and lipid processing. Nat. Genet. *51*, 414–430. https://doi.org/10.1038/s41588-019-0358-2.
15. Trajanoska, K., Seppala, L.J., Medina-Gomez, C., Hsu, Y.H., Zhou, S., van Schoor, N.M., de Groot, L.C.P.G.M., Karasik, D., Richards, J.B., Kiel, D.P., et al. (2020). Genetic basis of falling risk susceptibility in the UK Biobank Study. Commun. Biol. *3*, 543. https://doi.org/10.1038/s42003-020-01256-x.
16. Tikkanen, E., Gustafsson, S., Amar, D., Shcherbina, A., Waggott, D., Ashley, E.A., and Ingelsson, E. (2018). Biological insights into muscular strength: genetic findings in the UK biobank. Sci. Rep. *8*, 6451. https://doi.org/10.1038/s41598-018-24735-y.
17. Revez, J.A., Lin, T., Qiao, Z., Xue, A., Holtz, Y., Zhu, Z., Zeng, J., Wang, H., Sidorenko, J., Kemper, K.E., et al. (2020). Genome-wide association study identifies 143 loci associated with 25 hydroxyvitamin D concentration. Nat. Commun. *11*, 1647. https://doi.org/10.1038/s41467-020-15421-7.
18. Liu, M., Jiang, Y., Wedow, R., Li, Y., Brazel, D.M., Chen, F., Datta, G., Davila-Velderrain, J., McGuire, D., Tian, C., et al. (2019). Association studies of up to 1.2 million individuals yield new insights into the genetic etiology of tobacco and alcohol use. Nat. Genet. *51*, 237–244. https://doi.org/10.1038/s41588-018-0307-5.
19. Burgess, S., and Labrecque, J.A. (2018). Mendelian randomization with a binary exposure variable: interpretation and presentation of causal estimates. Eur. J. Epidemiol. *33*, 947–952. https://doi.org/10.1007/s10654-018-0424-6.
20. Day, F.R., Ruth, K.S., Thompson, D.J., Lunetta, K.L., Pervjakova, N., Chasman, D.I., Stolk, L., Finucane, H.K., Sulem, P., Bulik-Sullivan, B., et al. (2015). Large-scale genomic analyses link reproductive aging to hypothalamic signaling, breast cancer susceptibility and BRCA1-mediated DNA repair. Nat. Genet. *47*, 1294–1303. https://doi.org/10.1038/ng.3412.

21. Perry, J.R., Day, F., Elks, C.E., Sulem, P., Thompson, D.J., Ferreira, T., He, C., Chasman, D.I., Esko, T., Thorleifsson, G., et al. (2014). Parent-of-origin-specific allelic associations among 106 genomic loci for age at menarche. Nature *514*, 92–97. https://doi.org/10.1038/nature13545.

22. Mitchell, R.E., Bates, K., Wootton, R.E., Harroud, A., Richards, J.B., Davey Smith, G., and Munafò, M.R. (2020). Little evidence for an effect of smoking on multiple sclerosis risk: a Mendelian Randomization study. PLoS Biol. *18*, e3000973. https://doi.org/10.1371/journal.pbio.3000973.

23. Han, X., Ong, J.S., An, J., Hewitt, A.W., Gharahkhani, P., and MacGregor, S. (2020). Using Mendelian randomization to evaluate the causal relationship between serum C-reactive protein levels and age-related macular degeneration. Eur. J. Epidemiol. *35*, 139–146. https://doi.org/10.1007/s10654-019-00598-z.

24. Willer, C.J., Schmidt, E.M., Sengupta, S., Peloso, G.M., Gustafsson, S., Kanoni, S., Ganna, A., Chen, J., Buchkovich, M.L., Mora, S., et al. (2013). Discovery and refinement of loci associated with lipid levels. Nat. Genet. *45*, 1274–1283. https://doi.org/10.1038/ng.2797.

25. Ensrud, K.E. (2013). Epidemiology of fracture risk with advancing age. J. Gerontol. A Biol. Sci. Med. Sci. *68*, 1236–1242. https://doi.org/10.1093/gerona/glt092.

26. Livingston, G., Sommerlad, A., Orgeta, V., Costafreda, S.G., Huntley, J., Ames, D., Ballard, C., Banerjee, S., Burns, A., Cohen-Mansfield, J., et al. (2017). Dementia prevention, intervention, and care. Lancet *390*, 2673–2734. https://doi.org/10.1016/S0140-6736(17)31363-6.

27. Styrkarsdottir, U., Stefansson, O.A., Gunnarsdottir, K., Thorleifsson, G., Lund, S.H., Stefansdottir, L., Juliusson, K., Agustsdottir, A.B., Zink, F., Halldorsson, G.H., et al. (2019). GWAS of bone size yields twelve loci that also affect height, BMD, osteoarthritis or fractures. Nat. Commun. *10*, 2054. https://doi.org/10.1038/s41467-019-09860-0.

28. Jutberger, H., Lorentzon, M., Barrett-Connor, E., Johansson, H., Kanis, J.A., Ljunggren, O., Karlsson, M.K., Rosengren, B.E., Redlund-Johnell, I., Orwoll, E., et al. (2010). Smoking predicts incident fractures in elderly men: mr OS Sweden. J. Bone Miner. Res. *25*, 1010–1016. https://doi.org/10.1359/jbmr.091112.

29. Robbins, J., Aragaki, A.K., Kooperberg, C., Watts, N., Wactawski-Wende, J., Jackson, R.D., LeBoff, M.S., Lewis, C.E., Chen, Z., Stefanick, M.L., and Cauley, J. (2007). Factors associated with 5-year risk of hip fracture in postmenopausal women. JAMA *298*, 2389–2398. https://doi.org/10.1001/jama.298.20.2389.

30. Zhou, W., Nielsen, J.B., Fritsche, L.G., Dey, R., Gabrielsen, M.E., Wolford, B.N., LeFaive, J., VandeHaar, P., Gagliano, S.A., Gifford, A., et al. (2018). Efficiently controlling for case-control imbalance and sample relatedness in large-scale genetic association studies. Nat. Genet. *50*, 1335–1341. https://doi.org/10.1038/s41588-018-0184-y.

31. Chang, C.C., Chow, C.C., Tellier, L.C., Vattikuti, S., Purcell, S.M., and Lee, J.J. (2015). Second-generation PLINK: rising to the challenge of larger and richer datasets. GigaScience *4*, 7. https://doi.org/10.1186/s13742-015-0047-8.

32. Willer, C.J., Li, Y., and Abecasis, G.R. (2010). METAL: fast and efficient meta-analysis of genomewide association scans. Bioinformatics *26*, 2190–2191. https://doi.org/10.1093/bioinformatics/btq340.

33. Bulik-Sullivan, B.K., Loh, P.R., Finucane, H.K., Ripke, S., Yang, J., Schizophrenia Working Group of the Psychiatric Genomics, Consortium; Patterson, N., Daly, M.J., Price, A.L., and Neale, B.M. (2015). LD Score regression distinguishes confounding from polygenicity in genome-wide association studies. Nat. Genet. *47*, 291–295. https://doi.org/10.1038/ng.3211.

34. Bulik-Sullivan, B., Finucane, H.K., Anttila, V., Gusev, A., Day, F.R., Loh, P.R., ReproGen Consortium; and Robinson, E.B., et al.; Psychiatric Genomics Consortium; Genetic Consortium for Anorexia Nervosa of the Wellcome Trust Case Control Consortium 3 (2015). An atlas of genetic correlations across human diseases and traits. Nat. Genet. *47*, 1236–1241. https://doi.org/10.1038/ng.3406.

35. Zheng, J., Erzurumluoglu, A.M., Elsworth, B.L., Kemp, J.P., Howe, L., Haycock, P.C., Hemani, G., Tansey, K., Laurin, C., Early Genetics and Lifecourse Epidemiology EAGLE Eczema Consortium, et al. (2017). LD Hub: a centralized database and web interface to perform LD score regression that maximizes the potential of summary level GWAS data for SNP heritability and genetic correlation analysis. Bioinformatics *33*, 272–279. https://doi.org/10.1093/bioinformatics/btw613.

36. Kemp, J.P., Morris, J.A., Medina-Gomez, C., Forgetta, V., Warrington, N.M., Youlten, S.E., Zheng, J., Gregson, C.L., Grundberg, E., Trajanoska, K., et al. (2017). Identification of 153 new loci associated with heel bone mineral density and functional involvement of GPC6 in osteoporosis. Nat. Genet. *49*, 1468–1475. https://doi.org/10.1038/ng.3949.

37. Porcu, E., Medici, M., Pistis, G., Volpato, C.B., Wilson, S.G., Cappola, A.R., Bos, S.D., Deelen, J., den Heijer, M., Freathy, R.M., et al. (2013). A meta-analysis of thyroid-related traits reveals novel loci and gender-specific differences in the regulation of thyroid function. PLoS Genet. *9*, e1003266. https://doi.org/10.1371/journal.pgen.1003266.

38. CARDIoGRAMplusC4D Consortium; Deloukas, P., Kanoni, S., Willenborg, C., Farrall, M., Assimes, T.L., Thompson, J.R., Ingelsson, E., Saleheen, D., Erdmann, J., et al. (2013). Large-scale association analysis identifies new risk loci for coronary artery disease. Nat. Genet. *45*, 25–33. https://doi.org/10.1038/ng.2480.

39. Okada, Y., Wu, D., Trynka, G., Raj, T., Terao, C., Ikari, K., Kochi, Y., Ohmura, K., Suzuki, A., Yoshida, S., et al. (2014). Genetics of rheumatoid arthritis contributes to biology and drug discovery. Nature *506*, 376–381. https://doi.org/10.1038/nature12873.

40. Jostins, L., Ripke, S., Weersma, R.K., Duerr, R.H., McGovern, D.P., Hui, K.Y., Lee, J.C., Schumm, L.P., Sharma, Y., Anderson, C.A., et al. (2012). Host-microbe interactions have shaped the genetic architecture of inflammatory bowel disease. Nature *491*, 119–124. https://doi.org/10.1038/nature11582.

41. Bradfield, J.P., Qu, H.Q., Wang, K., Zhang, H., Sleiman, P.M., Kim, C.E., Mentch, F.D., Qiu, H., Glessner, J.T., Thomas, K.A., et al. (2011). A genome-wide meta-analysis of six type 1 diabetes cohorts identifies multiple associated loci. PLoS Genet. *7*, e1002293. https://doi.org/10.1371/journal.pgen.1002293.

42. Morris, A.P., Voight, B.F., Teslovich, T.M., Ferreira, T., Segrè, A.V., Steinthorsdottir, V., Strawbridge, R.J., Khan, H., Grallert, H., Mahajan, A., et al. (2012). Large-scale association analysis provides insights into the genetic architecture and pathophysiology of type 2 diabetes. Nat. Genet. *44*, 981–990. https://doi.org/10.1038/ng.2383.

43. Bowden, J., Davey Smith, G., and Burgess, S. (2015). Mendelian randomization with invalid instruments: effect estimation and bias detection through Egger regression. Int. J. Epidemiol. *44*, 512–525. https://doi.org/10.1093/ije/dyv080.

44. Yavorska, O.O., and Burgess, S. (2017). MendelianRandomization: an R package for performing Mendelian randomization analyses using summarized data. Int. J. Epidemiol. *46*, 1734–1739. https://doi.org/10.1093/ije/dyx034.

45. Brion, M.J.A., Shakhbazov, K., and Visscher, P.M. (2013). Calculating statistical power in Mendelian randomization studies. Int. J. Epidemiol. *42*, 1497–1501. https://doi.org/10.1093/ije/dyt179.

CellPress
OPEN ACCESS

STAR★METHODS

KEY RESOURCES TABLE

REAGENT or RESOURCE	SOURCE	IDENTIFIER
Deposited data		
GWAS meta-analyses	This article	GWAS catalog, https://www.ebi.ac.uk/gwas/
Software and algorithms		
SAIGE	Zhou et al. 2018[30]	https://github.com/weizhouUMICH/SAIGE
PLINK	Chang et al. 2015[31]	https://www.cog-genomics.org/plink/1.9/
METAL	Willer et al. 2010[32]	https://genome.sph.umich.edu/wiki/METAL
LDSR	Bulik-Sullivan et al. 2015[33]	https://github.com/bulik/ldsc
R	The R Project for Statistical Computing	https://www.r-project.org/version 4.0.1

RESOURCE AVAILABILITY

Lead contact

Further information and requests for resources should be directed to and will be fulfilled by the lead contact Claes Ohlsson (claes.ohlsson@medic.gu.se).

Materials availability

This study did not generate unique reagents.

Data and code availability

- The summary statistics of the present GWAS meta-analysis on hip fractures will be deposited to the GWAS catalog (*https://www.ebi.ac.uk/gwas/studies/GCST90161240*) upon publication of the article. Additional datasets that have been used for analysis can be found on the links to their corresponding reference papers.
- This paper does not report original code.
- Any additional information required to reanalyze the data reported in this work is available from the lead contact upon request.

EXPERIMENTAL MODEL AND SUBJECT DETAILS

Study population

We included subjects from five biobanks (*UFO* from Sweden; *HUNT* from Norway; *UK-Biobank* from UK; *Estonian Biobank* from Estonia and *FinnGen* from Finland) from some of the major Northern European biobanks with hip fracture data, relevant covariates, and genotype data available. To reduce potential bias due to population stratification, we restricted the analyses to studies with participants of European descent. In total 11,516 hip fracture cases and 723,838 controls were included. The study was approved by the local ethics review boards and study subjects provided written informed consent. For detailed information on the five contributing biobanks, please see supplemental methods (Tables S1–S4).

METHOD DETAILS

Hip fracture definition

To specifically evaluate hip fractures, we only included hip fractures derived from high quality national registers based on medical and/or radiological reports and classified according to International Classification of Diseases (ICD; corresponding to ICD10 codes S72.0, S72.1 and S72.2 and ICD9 code 820; HUNT, UK Biobank, UFO, Estonian Biobank). Only hip fracture cases >30 years old were included. No self-reported hip fractures were included. Controls were defined as individuals from the same cohorts, without a history of hip fracture. For the FinnGen cohort, only public available summary statistics were available and for that analysis, a wider definition of hip fractures had been used corresponding to the ICD10 code S72, including not only the most common hip fractures (S72.0, S72.1 and S72.2) but also the less common diaphyseal and distal femur fractures. Predicted from the observed distribution in the large UK-Biobank, ~90% of the femur fractures in the FinnGen would be hip fractures. To achieve effect estimates not confounded by a possible minor dilution by diaphyseal and distal femur fractures in the FinnGen cohort, we replicated the five genome-wide signals (Table 1) and the significant causal risk factors (Table 3) for hip fracture in a meta-analysis excluding the FinnGen cohort, yielding similar effect estimates.

QUANTIFICATION AND STATISTICAL ANALYSIS

Genome-wide association study and meta-analyses

Genome-wide genotyping was performed in each cohort by use of Illumina or Affymetrix genome-wide genotyping chips and imputation was performed to ensure accurate ascertainment of nearly all common genetic variation above a minor allele frequency threshold of 1% (Table S4). We followed a standardized analytical plan to assess the association of single nucleotide polymorphisms (SNPs) with risk of hip fracture in each participating cohort. Logistic models using the SAIGE or PLINK software were used to estimate the SNP associations with hip fracture, adjusted for gender, age (simple and quadratic terms), height, weight, principal components, study site (when necessary) and family structure (if feasible), testing additive (per allele) genetic effects. Information on covariates for adjustments and study designs for the five included cohorts are presented in Table S1. When needed, individual GWAS summary results were corrected for population stratification by the genomic control inflation factor before we performed fixed effect inverse-variance weighted meta-analysis using METAL software. A total of 9,457,767 autosomal and X-linked SNPs present in more than two studies were meta-analysed. Allele and genotype frequencies of all genotyped variants followed Hardy-Weinberg equilibrium proportions. Further exploratory age- (divided by the median age of the hip fracture cases, 71.2 years) and gender-stratified sub-analyses were also performed.

Genetic correlation

To estimate the genetic correlation between hip fractures and other complex traits and diseases, we used (cross-trait) Linkage disequilibrium score regression (LDSR)[34] as implemented in the online web utility LDHub.[35] This method uses the cross-products of summary test statistics from two GWASs and regresses them against a measure of how much variation each SNP tags (its LD score).[36] The LDSR analyses were restricted to HapMap3 SNPs with MAF >5% in the 1000 Genomes European reference population. We used pre-calculated LD scores from the same reference population (https://data.broadinstitute.org/alkesgroup/LDSCORE/).

In general, the selection of plausible clinical risk factors for evaluation of genetic correlation with hip fractures in Table 2 and for MR in Table 3 was similar as reported in a previous GWAS on fractures at any bone site.[7] From the variety of traits available on LDHub,[33] we selected 10 plausible risk factors for hip fractures.[15] In addition, locally, we used the LDSCORE tool available from LDHub to estimate the genetic correlation between hip fracture risk and seven additional plausible risk factors for hip fractures using available GWAS summary statistics for fractures at any bone site,[7] estimated BMD by ultrasound in the heel (eBMD),[11] grip strength,[16] vitamin D levels,[17] falls,[15] alcohol consumption,[18] and Alzheimer's disease.[14] We accounted for multiple testing by using a conservative Bonferroni correction for 17 tests ($p < 0.05/17 = 0.0029$).

Mendelian randomization

To assess causal associations between plausible risk markers and hip fractures, we performed Two-sample mendelian randomization (MR) analyses and these associations were also compared with the effects on fractures at any bone site using summary statistics derived from a previous GWAS meta-analysis.[11] We used genetic instrument variables obtained from selected GWAS as proxies for femoral neck BMD (FN-BMD),[10] lumbar spine BMD (LS-BMD),[10] eBMD,[11] menopause[7,20] puberty,[7,21] thyroid-stimulating hormone (TSH),[7,37] grip strength,[16] vitamin D levels,[17] Alzheimer's disease,[14] coronary heart disease,[7,38] rheumatoid arthritis,[39] inflammatory bowel disease,[7,40] type 1 diabetes,[7,41] type 2 diabetes,[7,42] and ever smoked regularly.[18] Although alcohol consumption and falls are plausible causal risk factors for hip fractures, these were not included in the MR analyses as the available genetic instruments were very weak, resulting in insufficient power in the analyses (Table S6).[15,18] As genetic variants are randomly distributed at birth, they are unaffected by confounders. As the primary MR analyses, we used combined weighted estimates by an inverse-variance weighted (IVW) approach using fixed or random effects depending on Cochran's Q statistic test of heterogeneity. We applied a conservative Bonferroni corrected threshold to account for the multiple testing (i.e., $p < 0.05/15 = 0.0033$, because 15 exposures were assessed). We then used the MR-Egger method as a sensitivity analysis to avoid possible uncontrolled pleiotropy. This method uses a weighted regression with an unconstrained intercept to regress the effect sizes of variant risk factor associations. It can thus detect some violations of the standard MR assumptions and provide an effect estimate, which is not subject to these violations.[43] In further sensitivity analyses, we used the penalized weighted median MR method and the weighted median MR methods. The MR analyses were conducted with the R-package MendelianRandomization.[44]

R-Code for MR:

```
library("MendelianRandomization")
mrin < - mr_input(bx = …,bxse = …,by = …, byse = …) #bx & bxse are set to Beta and SE for the exposure (risk factor) and by & byse are set to Beta and SE for the outcome (fractures).
mrivwfix < - mr_ivw(mrin, model = "fixed")
mrivwrand < - mr_ivw(mrin, model = "random")
if(nrow(data)≥3){mrweightmed < - mr_median(mrin, weighting = "weighted")}
if(nrow(data)≥3){mrpenwightmed < - mr_median(mrin, weighting = "penalized")}
if(nrow(data)≥3){mregger < - mr_egger(mrin)}"
```

Power calculation

Calculations were performed to test whether our MR studies were adequately powered to detect a significant change mainly in the hip fracture outcomes but also in the fracture at any bone site outcome with the IVW method. For each trait, we used the variance explained by the instruments variables (R^2 for continuous risk factors and available pseudo R^2 for binary risk factors) reported in the GWAS publications, the proportion of fracture cases and the sample size, to estimate the power to detect different OR of 1.15 and 1.20 ($\alpha = 0.05/15 = 0.0033$; http://cnsgenomics.com/shiny/mRnd/).[45]

Cell Reports Medicine, Volume *3*

Supplemental information

Assessment of the genetic and clinical determinants of hip fracture risk: Genome-wide association and Mendelian randomization study

Maria Nethander, Eivind Coward, Ene Reimann, Louise Grahnemo, Maiken E. Gabrielsen, Carl Wibom, Estonian Biobank Research Team, Reedik Mägi, Thomas Funck-Brentano, Mari Hoff, Arnulf Langhammer, Ulrika Pettersson-Kymmer, Kristian Hveem, and Claes Ohlsson

A

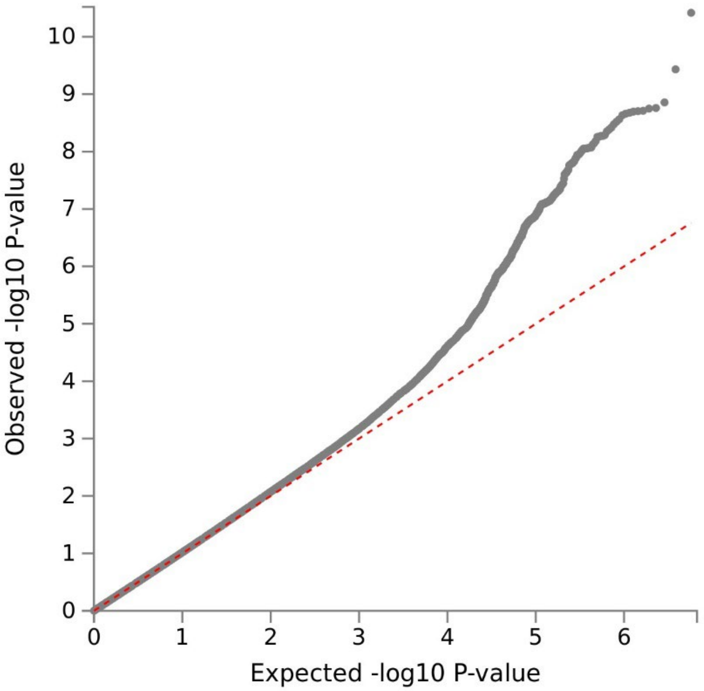

B

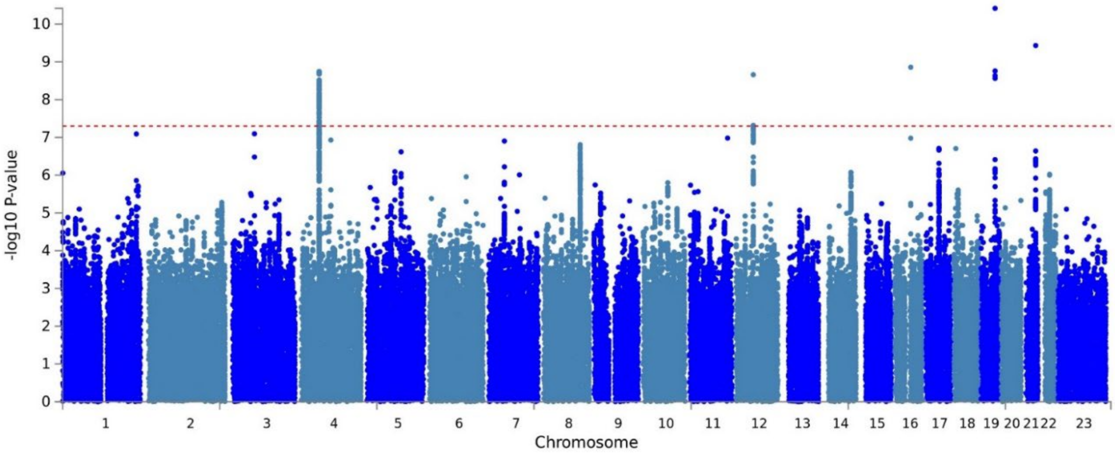

Figure S1: Quantile-quantile (Q-Q (A) and Manhattan Plot (B). Related to Table 1.

(A) Quantile-quantile (Q-Q) plot of observed versus expected P values of the GWAS results. The straight line in the Q-Q plot indicates the distribution of SNPs under the null hypothesis.

(B) Manhattan Plot of Association Statistics ($-\log_{10}(P)$) for hip fracture risk for the meta-analysis. Each dot represents a SNP, and the x axis indicates its chromosomal position (built 37 NCBI). Dashed horizontal red line marks the genome-wide significance threshold ($p < 5 \times 10^{-8}$).

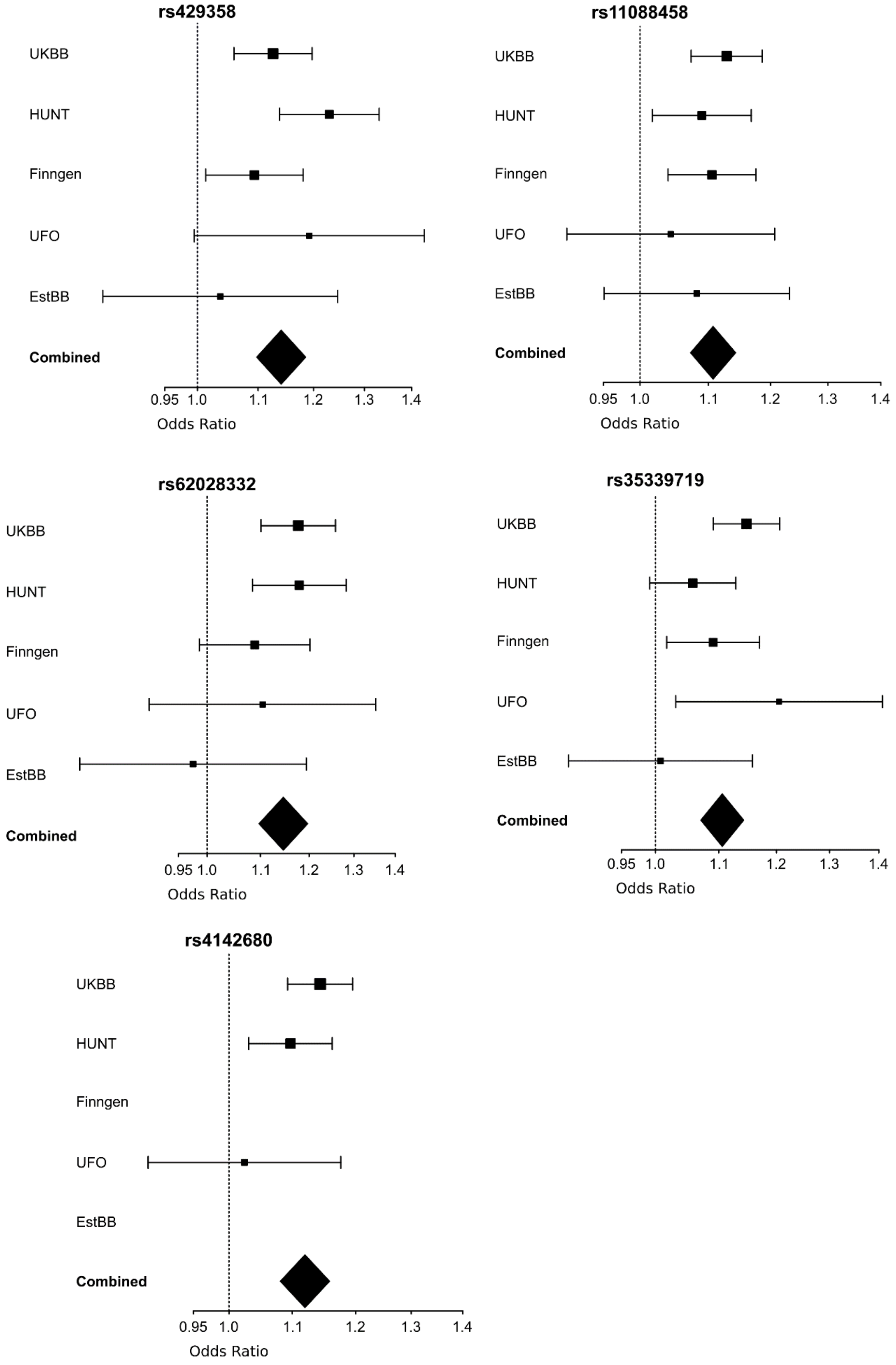

Figure S2: **Forest plots for the lead signal in each of the five loci associated with hip fracture risk.** Related to Table 1.

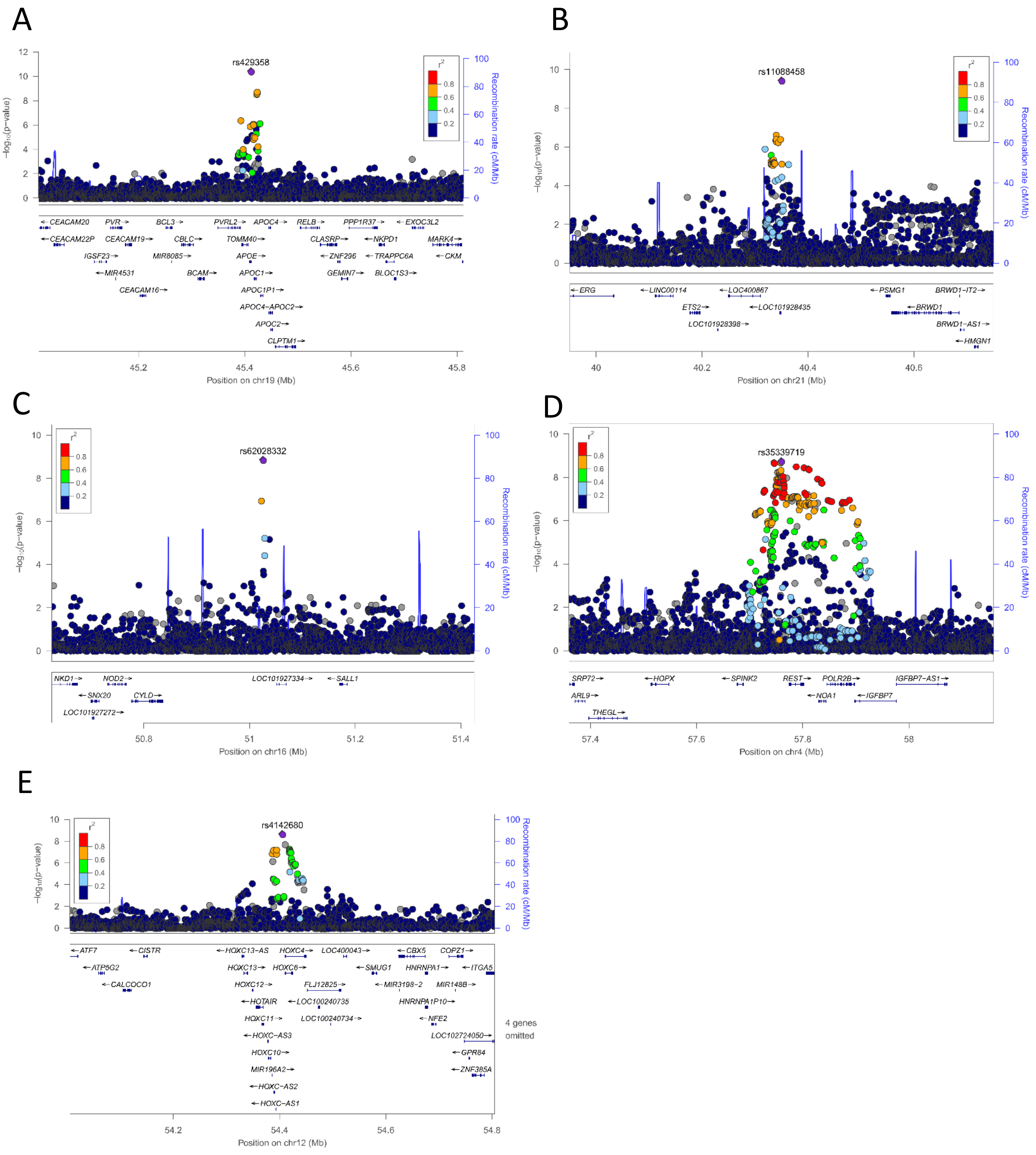

Figure S3: Regional association plots of top signals. Related to Table 1. (A). *APOE* region, 19q13.3219; (B). *ETS2* region 21q22.221; (C). *SALL1* region 16q12.116; (D). *REST* region 4q12; (E). *HOXC8* region 12q13.1312. SNPs are plotted by position in 500kb window against association with hip fracture risk (-log10 P). Plot highlighting the most significant SNPs in the hip fracture meta-analysis.

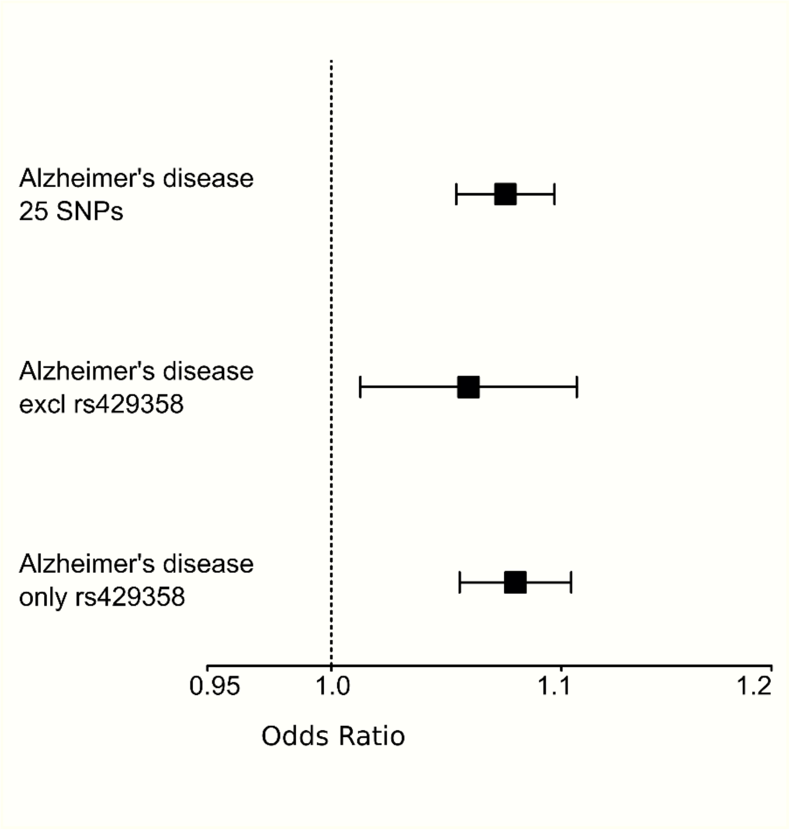

Figure S4: Mendelian randomization to estimate the causal effects of Alzheimer's disease on hip fractures. Related to Fig 1. (i) including all 25 available SNPs (=25 SNPs), (ii) excluding the strong signal in the *APOE*-region (=24 SNPs) or (iii) only using the strong SNP in the *APOE*-region (1 SNP). (Kunkle et al., 2019) Inverse variance weighted meta-analyses are performed. Odds ratios are for the risk of hip fracture per doubling of odds of Alzheimer's disease. Estimates are displayed using a random effects model to account for possible heterogeneity.

Table S1 Descriptive Study Design. Related to STAR Methods.

Short name	Full name	Study Design	Area of Origin	Ethnicity	Study Type	Total Sample Size with DNA, Covariates and Phenotype Information for the present study (N)	Study Description	References
**UKBB**	UK Biobank	Cohort study. Both prevalent and incident hip fractures were identified by ICD codes (ICD10, S72.0, S72.1, S72.2; ICD9, 820) from registers and fracture cases >30 years were included. Controls were defined as individuals from the same cohort without a history of hip fracture. Height and weight were derived from the UK-Biobank baseline visit. For fracture cases, age was the time for first hip fracture and for controls the age was the age of censoring (either death, emigration or time of evaluation in the registers). The UK Biobank has ethical approval from the Northwest Multicentre Research Ethics Committee, and informed consent was obtained from all participants.	UK	North-Western European subset used for analysis in the present study	Population based	438937	In 2006-2010, the UK Biobank recruited 502,643 individuals aged between 37 and 73 years (99.5% were 40-69 years) from across the country. Each participant provided a large amount of information regarding their health and lifestyle using touch screen questionnaires, physical measurements and agreement to have their health followed and they also provided blood, urine and saliva samples for future analysis.	Biobank UK. UK Biobank: Protocol for a Large-scale Prospective Epidemiological Resource. 2010. http://www.ukbiobank.ac.uk/wp-content/uploads/2011/11/UKBiobank-Protocol.pdf
**FinnGen**	FinnGen	The FinnGen study is a mixture of population-based cohorts and samples from hospital biobanks. Hip/femur fractures were identified by ICD codes. Both prevalent and incident hip fractures were identified by ICD codes (ICD10, S72; ICD9 820). We used publicly available summary statistics data from the 3rd data release from the FinnGen study (https://www.finngen.fi/en/access_results). The FinnGen analyses were adjusted for age, gender, genotyping chip, genetic relationship and first 10 PCs but not for height or weight. To achieve effect estimates not confounded by a possible minor dilution by diaphyseal and distal femur fractures and lack of adjustment for height and weight in the publicly available looked analyses of the FinnGen cohort, we replicated the significant causal associations in a meta-analysis excluding the FinnGen cohort, yielding similar effect estimates (Tables 1 and 3).	Finland	Northern European/Finnish	Population based	116375	FinnGen is a public-private partnership project combining genotype data from Finnish biobanks and digital health record data from Finnish health registries. FinnGen provides a unique opportunity to study genetic variation in relation to disease trajectories in an isolated population.<br>FinnGen is a growing project, aiming at 500,000 individuals in 2023. The data in the present study includes 116375 participants in the FinnGen, Data Freeze 3. FinnGen comprises prospective epidemiological cohorts (initiated as far back as 1992), disease-based cohorts, and hospital biobank samples. The unique national personal identification number links the genotypes to the national hospital discharge registry (1968-), the national death registry (1969-) and the medication reimbursement registry (1964-). These registries cover the whole population.	PMID33318493
**UFO**	Umeå Fracture and Osteoporosis Study	Case-Control study. Both prevalent and incident hip fractures were identified by ICD codes (ICD10, S72.0, S72.1, S72.2; ICD9, 820) and fracture cases >30 years were included. All fracture cases were also confirmed by radiological report and or medical records. Height and weight were retrieved from the closest visit and age is the age at the hip fracture. The UFO-hip fracture controls were age-matched without previous hip fracture and were drawn from the controls of a previous GWAS study of glioma (PMID 22886559). The UFO study was approved by the local research ethics committee at Umeå University. Written consent was obtained from all participants.	Sweden/Umeå	Northern European	Population based	1813	The UFO study is case-control study investigating associations between genes, lifestyle and osteoporotic fractures. The study is based on the prosepctive and populationbased Northern Sweden Health and Disease Study cohort, initiated to assess risk factors for diabetes and cardiovascular disease.	PMID20464545; PMID:1466024
**HUNT**	The Trøndelag Health Study	Cohort study. Both prevalent and incident hip fractures were identified by ICD codes (ICD10, S72.0, S72.1, S72.2; ICD9, 820) from registers and fracture cases >30 years were included. Controls were defined as individuals from the same cohorts, without a history of hip fracture. Height and weight were derived from the latest available HUNT visit. For fracture cases, age was the time for first hip fracture and for controls the age was the age at the HUNT visit for the height and weight measurements. Participation in the HUNT Study is based on informed consent and the study has been approved by the Regional Ethics Committee for Medical Research in Norway.	Norway/Trøndelag	Northern European	Population based	68819	The Trøndelag Health Study (HUNT) comprises data and samples obtained through four population studies between 1984 and 2019. 230,000 people from the county of Trøndelag have participated with questionnaire data, and and almost 120,000 participants have submitted biological samples. Even more than 120.000 have contributed with anthropometric measurements such as height, weight, blood pressure and many other measurements.	PMID22879362
**EstBB**	Estonian Biobank	Cohort study. Both prevalent and incident hip fractures were identified by ICD codes (ICD10, S72.0, S72.1, S72.2; ICD9, 820) from registers and fracture cases >30 years were included. Controls were defined as individuals from the same cohorts, without a history of hip fracture. Height and weight were derived from the latest visit. For fracture cases, age was the time for first hip fracture and for controls the age was age at recruitment to EstBB. Estonian Biobank has ethical approval from the Estonian Commitee on Bioethics and Human Research at the Ministry of Social Affairs (No 1.1-12/624). Written informed consent for participation was obtained from all study subjects.	Estonia/Tartu	Northern European	Population based	109410	The Estonian Biobank cohort is a volunteer-based sample of the Estonian resident adult population (aged ≥18 years). Estonians represent 83%, Russians 14%, and other nationalities 3% of all participants. The current number of participants is close to 200,000 and represents a large proportion, > 15 %, of the Estonian adult population, making it ideally suited to population-based studies. General practitioners (GPs) and medical personnel in the special recruitment offices have recruited participants throughout the country. At baseline, the GPs performed a standardized health examination of the participants, who also donated blood samples for DNA, white blood cells and plasma tests and filled out a 16-module questionnaire on health-related topics such as lifestyle, diet and clinical diagnoses described in WHO ICD-10. A significant part of the cohort has whole genome sequencing (3000), whole exome sequencing (2500), genome-wide single nucleotide polymorphism (SNP) array data (>200 000) and/or NMR metabolome data (11 000) available. The data are continuously updated through periodical linking to national electronic databases and registries. A part of the cohort has been re-contacted for follow-up purposes and resampling, and targeted invitations are possible for specific purposes, for example people with a specific diagnosis. For the current study data freeze including approximately 150,000 gene donors was applied, which resulted in including 109410 gene donors into the study.	PMID24518929, https://genomics.ut.ee/en/access-biobank

Table S2 Study-specific descriptive statistics. Related to STAR Methods.

		Women			Men		
**Short name**	**Trait**	**N**	**mean**	**SD**	**N**	**mean**	**SD**
**UKBB**	Age (yrs)	238565	68.3	7.9	200372	68.5	8.1
	Height (cm)	238565	162.6	6.2	200372	175.8	6.8
	Weight (kg)	238565	71.4	13.9	200372	86.2	14.3
**HUNT**	Age (yrs)	36377	52.73	18.14	32442	52.61	17.33
	Height (cm)	36377	164.1	6.51	32442	177.32	6.83
	Weight (kg)	36377	72.43	13.66	32442	85.47	13.39
**UFO**	Age (yrs)	1034	64.7	11.09	779	60.88	11.16
	Height (cm)	1034	163.4	5.96	779	176.74	6.67
	Weight (kg)	1034	68.0	12.68	779	81.45	12.05
**EstBB**	Age (yrs)	73617	43.65	15.90	35793	43.04	16.34
	Height (cm)	73617	166.00	6.36	35793	179.85	7.06
	Weight (kg)	73617	70.85	15.04	35793	86.39	15.63

For the public available **FinnGen** summary statistic data, the mean age = 60.6 yrs (SD 16.5 yrs) and 56.3% of the subjects are women. We do not have height or weight data for these subjects and we do not have age or gender stratified analyses for FinnGen.

Table S3 study-specific fracture counts. Related to STAR Methods.

Study	Fracture assessment method	Fracture cases (n)	Controls (n)	All (n)	Median age at fracture (yrs)
**UKBB**	Medical records; ICD codes; ICD10 S72.0; S72.1; S72.0; ICD9 820	4035	434902	438937	69
**HUNT**	Medical records; ICD codes; ICD10 S72.0; S72.1; S72.0; ICD9 820	3404	65415	68819	80
**UFO**	Medical records and radiographic verification ; ICD codes; ICD10 S72.0; S72.1; S72.0; ICD9 820	962	851	1813	64
**EstBB**	Medical records; ICD codes; ICD10 S72.0; S72.1; S72.0; ICD9 820	633	108777	109410	75
**FinnGen**	Medical records; ICD codes; ICD10 S72; ICD9 820	2482	113893	116375	NA
		11516	723838	735354	

NA = not available

Table S4 Genotypeing, study-specific fracture counts. Related to STAR Methods.

Cohort	Genotyping				Imputation			Association Analyses			λ
	Platform	Genotype Calling Algorithm	Call Rate	P for HWE	Imputation Software	Reference panel	Imputation Quality	MAF	Analysis Software	No. analyzed SNPs	
UK Biobank	UK Biobank Axiom Array (N~450,000) and UK BiLEVE Array (N~50,000)	Affymetrix Power Tools software and the Affymetrix Best Practices Workflow	≥90%	$>10^{-12}$	Phasing: Modified version of SHAPEIT2; Imputation: modified version of IMPUTE2	HRC v1.1 and UK10K + 1000 Genomes panel	≥0.3	>1%	SAIGE	9 576 610	1.00
HUNT	Illumina HumanCoreExome arrays: 12 v1.0, 12 v1.1, UM HUNT Biobank v1.0 (HumanCoreExome 24 with custom content)	Illumina GenomeStudio	≥99%	$>10^{-4}$	Minimac3	HRC v1.1 and 2201 WGS samples from HUNT	≥0.3	>1%	SAIGE	8 825 549	1.02
FinnGen	Illumina (Illumina Inc., San Diego, CA, USA) and Affymetrix arrays (Thermo Fisher Scientific, Santa Clara, CA, USA)	GenCall and zCall algorithms for Illumina and AxiomGT1 algorithm for Affymetrix data	≥95%	$>10^{-6}$	Pre-phased using Eagle 2.3.5, imputed using Beagle 4.1 (version 08Jun17.d8b)	SISu v3 imputation reference panel	≥0.95	>1%	SAIGE	8 696 849	0.99
EstBB	Illumina GSAv1.0, GSAv2.0, and GSAv2.0_EST arrays	Illumina GenomeStudio v2.0.4	≥95%	$>10^{-4}$	Pre-phased using Eagle v2.3, imputed using Beagle v.28Sep18.793	Estonian population specific imputation reference of 2297 WGS samples were used	≥0.3	>1%	SAIGE	8 664 150	0.92
UFO	Illumina HumanHap660 arrays	BeadStudio	≥97.5%	$>10^{-6}$	Pre-phased using Eagle v2.4, imputed using PBWT	HRC v1.1	≥0.3	>1%	PLINK	8 002 885	1.06

Table S5 Genome wide significant nucleotide polymorphisms for hip fractures combined in the meta-analyseis and for each included cohort. Related to Table 1.

									GWAS meta-analyses					Heterozygosity	
Locus	Candidate Gene	Distance to gene (kb)	SNP	CHR	EA	OA	EAF	EAFSE	N	Ncases	OR	(95% CI)	P	I^2	P
19q13.32	*APOE*	0.0	rs429358	19	C	T	0.17	0.02	735354	11516	1.14	(1.10 - 1.19)	3.8E-11	34.6	0.19
21q22.2	*ETS2*	153.2	rs11088458	21	G	A	0.70	0.04	735354	11516	1.11	(1.07 - 1.14)	3.7E-10	0	0.83
16q12.1	*SALL1*	144.4	rs62028332	16	G	A	0.87	0.02	735354	11516	1.15	(1.10 - 1.20)	1.4E-09	13.9	0.33
4q12	*REST*	14.5	rs35339719	4	G	A	0.74	0.02	735354	11516	1.11	(1.07 - 1.14)	1.8E-09	41.3	0.15
12q13.13	*HOXC8*	0.0	rs4142680	12	T	C	0.41	0.01	509569	8401	1.12	(1.08 - 1.16)	2.2E-09	26.5	0.26

	UKBB					HUNT				
Locus	OR	95% CI	P	N	Ncases	OR	95% CI	P	N	Ncases
19q13.32	1.13	(1.06 - 1.20)	1.5E-04	438937	4035	1.23	(1.14 - 1.33)	2.1E-07	68819	3404
21q22.2	1.13	(1.07 - 1.19)	1.8E-06	438937	4035	1.09	(1.02 - 1.17)	1.5E-02	68819	3404
16q12.1	1.18	(1.10 - 1.26)	1.7E-06	438937	4035	1.18	(1.08 - 1.28)	1.2E-04	68819	3404
4q12	1.15	(1.09 - 1.21)	7.2E-08	438937	4035	1.06	(0.99 - 1.13)	8.9E-02	68819	3404
12q13.13	1.14	(1.09 - 1.19)	3.9E-08	438937	4035	1.09	(1.03 - 1.16)	3.9E-03	68819	3404

	FinnGen					UFO					EstBB				
Locus	OR	95% CI	P	N	Ncases	OR	95% CI	P	N	Ncases	OR	95% CI	P	N	Ncases
19q13.32	1.09	(1.01 - 1.18)	2.2E-02	116375	2482	1.19	(1.00 - 1.43)	5.6E-02	1813	962	1.04	(0.86 - 1.25)	7.0E-01	109410	633
21q22.2	1.11	(1.04 - 1.18)	1.3E-03	116375	2482	1.04	(0.90 - 1.21)	5.6E-01	1813	962	1.08	(0.95 - 1.23)	2.3E-01	109410	633
16q12.1	1.09	(0.99 - 1.20)	9.3E-02	116375	2482	1.10	(0.90 - 1.35)	3.4E-01	1813	962	0.98	(0.80 - 1.19)	8.1E-01	109410	633
4q12	1.09	(1.02 - 1.17)	1.5E-02	116375	2482	1.20	(1.03 - 1.41)	1.9E-02	1813	962	1.01	(0.88 - 1.16)	9.1E-01	109410	633
12q13.13	NA	NA	NA	NA	NA	1.02	(0.89 - 1.17)	7.5E-01	1813	962	NA	NA	NA	NA	NA

OR = Odds ratio NA = not available, EA = effect allele, EAF Effect allele frequency, OA = other allele, I^2 = index of heterogenity, CHR = chromosome, SNP = single nucleotide polymorphism, CI = confidence interval

Table S6 Number of SNPs and Variance explained by Instrumental Variables for Each Risk Factor. Related to Fig 1.

Trait or disease	N SNPs	Total R^2	Total Sample Size in reference GWAS	PMID
Continuous risk factor				
**BMD-related parameters**				
Decreased FN-BMD	47	5.8	32 961	22504420
Decreased LS-BMD	45	6.4	31 800	22504420
Decreased eBMD	457	20	426 824	30598549
**Other risk markers**				
Early menopause	54	6	69 360	26414677 + 30158200
Late puberty	106	2.7	182 416	25231870 + 30158200
Decreased TSH	20	5.6	26 523	23408906 + 30158200
Decreased grip strength/BW	130	1.7	223 315	29691431
Low vitamin D levels	103	7.5	417 580	32242144
Binary risk factor				
Alzheimer´s disease	25	31	63926 (21982 cases)	30820047
Coronary heart disease	38	5.7	107 432 (41 513 cases)	23202125 + 30158200
Rheumatoid arhritis	57	5.5	58 284 (14 361 cases)	24390342
Inflammatory bowel disease	149	7.5	34 652 (12 882 cases)	23128233 + 30158200
Type 1 diabetes	19	6.7	26 890 (9934 cases)	21980299 + 30158200
Type 2 diabetes	38	5.7	56 862 (12 171 cases)	22885922 + 30158200
Ever smoked regularly	367	2.3	1 232 091 (NA)	30643251
Risk factors with insufficient power for MR analyses				
Falls	3	0.28	362 103 (89 076 cases)	32999390
Alcohol Consumption	96	0.2	941 280	30643251

NA= not available, BMD = bone mineral density, TSH = Thyroid-stimulating hormone, FN = femoral neck, LS = lumbar spine, eBMD = estimated BMD in the heel using ultrasound

Table S7 Mendelian randomization to estimate the causal effects of 15 genetically determined risk factors on hip fracture and fracture at any bone site using a variety of MR methods. Related to Fig 1.

Hip fracture

			CochranQ		Inverse variance weighted fixed effects			Inverse variance weighted random effects			Weighted median			Penalized weighted median			Egger Intercept		Egger Estimate		
**Trait or disease**	N SNPs	PMID	Est	P	OR	95% CI	P	OR	95% CI	P	OR	95% CI	P	OR	95% CI	P	Beta	P	OR	95% CI	P
Continuous risk factor																					
**BMD-related parameters**																					
Decreased FN-BMD	47	22504420	117.30	**3.8E-08**	2.12	(1.93 - 2.33)	**6.7E-54**	2.12	(1.82 - 2.47)	**3.7E-22**	2.03	(1.72 - 2.40)	**5.6E-17**	2.00	(1.70 - 2.37)	**3.3E-16**	0.02	1.2E-01	1.49	(0.93 - 2.38)	9.9E-02
Decreased LS-BMD	45	22504420	194.69	**7.5E-21**	1.66	(1.52 - 1.82)	**3.6E-29**	1.66	(1.38 - 2.00)	**9.9E-08**	1.55	(1.31 - 1.83)	**2.6E-07**	1.59	(1.34 - 1.88)	**7.7E-08**	0.05	**5.5E-04**	0.71	(0.43 - 1.19)	1.9E-01
Decreased eBMD	457	30598549	925.89	**3.2E-34**	1.73	(1.63 - 1.83)	**1.0E-78**	1.73	(1.59 - 1.87)	**1.1E-39**	1.73	(1.54 - 1.94)	**5.6E-21**	1.87	(1.67 - 2.09)	**5.5E-28**	0.00	6.4E-02	1.55	(1.35 - 1.78)	**6.3E-10**
**Other risk markers**																					
Early menopause	54	26414677	40.46	9.0E-01	0.95	(0.87 - 1.04)	3.0E-01	0.95	(0.87 - 1.04)	3.0E-01	0.97	(0.84 - 1.12)	7.0E-01	0.97	(0.84 - 1.12)	7.1E-01	-0.01	2.3E-01	1.08	(0.86 - 1.36)	4.9E-01
Only females	54	26414677	57.68	3.1E-01	1.01	(0.89 - 1.15)	8.4E-01	1.01	(0.88 - 1.16)	8.5E-01	1.02	(0.82 - 1.26)	8.9E-01	1.02	(0.82 - 1.26)	8.8E-01	-0.01	9.6E-02	1.30	(0.94 - 1.80)	1.1E-01
Late puberty	106	25231870	131.73	4.0E-02	1.13	(1.02 - 1.26)	1.7E-02	1.13	(1.01 - 1.27)	3.3E-02	1.26	(1.08 - 1.48)	4.0E-03	1.28	(1.09 - 1.50)	**2.6E-03**	-0.01	3.2E-02	1.64	(1.15 - 2.35)	6.5E-03
Only females	106	25231870	109.70	3.6E-01	1.18	(1.02 - 1.37)	2.5E-02	1.18	(1.02 - 1.38)	2.8E-02	1.45	(1.14 - 1.85)	**2.8E-03**	1.47	(1.15 - 1.88)	**2.3E-03**	-0.01	2.5E-01	1.55	(0.96 - 2.50)	7.3E-02
Onlly males	106	25231870	113.16	2.8E-01	1.02	(0.84 - 1.24)	8.7E-01	1.02	(0.83 - 1.25)	8.7E-01	1.19	(0.88 - 1.61)	2.7E-01	1.19	(0.88 - 1.61)	2.6E-01	-0.03	4.1E-03	2.42	(1.30 - 4.51)	5.4E-03
Decreased TSH	20	23408906	21.42	3.1E-01	1.01	(0.92 - 1.11)	8.5E-01	1.01	(0.91 - 1.11)	8.6E-01	0.93	(0.82 - 1.07)	3.2E-01	0.93	(0.81 - 1.06)	2.9E-01	0.01	4.8E-01	0.90	(0.65 - 1.26)	5.4E-01
Decreased grip strength/BW	130	29691431	192.11	**2.6E-04**	1.06	(0.90 - 1.25)	4.8E-01	1.06	(0.87 - 1.30)	5.6E-01	1.04	(0.80 - 1.35)	7.8E-01	1.02	(0.78 - 1.33)	9.1E-01	-0.01	3.8E-01	1.56	(0.64 - 3.78)	3.3E-01
Low vitamin D levels	103	32242144	142.54	5.0E-03	0.98	(0.88 - 1.08)	6.6E-01	0.98	(0.87 - 1.10)	7.1E-01	0.99	(0.85 - 1.15)	9.0E-01	0.99	(0.85 - 1.15)	9.0E-01	0.00	3.8E-01	1.02	(0.87 - 1.19)	7.9E-01
Binary risk factor																					
Alzheimer´s disease	25	30820047	17.85	8.1E-01	1.07	(1.05 - 1.10)	**1.9E-12**	1.07	(1.05 - 1.10)	**1.9E-12**	1.08	(1.06 - 1.10)	**2.1E-11**	1.08	(1.06 - 1.10)	**2.1E-11**	0.00	7.2E-01	1.08	(1.05 - 1.10)	**3.6E-09**
Coronar heart disease	38	23202125	61.13	7.5E-03	1.01	(0.96 - 1.07)	5.9E-01	1.01	(0.95 - 1.08)	6.7E-01	0.98	(0.91 - 1.06)	6.6E-01	0.97	(0.90 - 1.05)	4.9E-01	-0.01	4.0E-01	1.11	(0.90 - 1.36)	3.5E-01
Rheumatoid arhritis	57	24390342	55.53	4.9E-01	1.00	(0.97 - 1.02)	9.3E-01	1.00	(0.97 - 1.02)	9.3E-01	1.00	(0.96 - 1.04)	9.1E-01	1.00	(0.97 - 1.04)	8.8E-01	0.00	6.3E-01	1.01	(0.96 - 1.06)	7.1E-01
Inflamatory bowel disease	149	23128233	179.65	3.9E-02	1.01	(1.00 - 1.03)	8.9E-02	1.01	(1.00 - 1.03)	1.2E-01	0.99	(0.97 - 1.02)	6.2E-01	0.99	(0.96 - 1.01)	2.8E-01	0.00	3.6E-01	1.00	(0.96 - 1.04)	9.2E-01
Type 1 diabetes	19	21980299	26.64	8.6E-02	1.00	(0.99 - 1.02)	8.2E-01	1.00	(0.98 - 1.02)	8.6E-01	1.00	(0.98 - 1.02)	9.8E-01	1.00	(0.98 - 1.02)	1.0E+00	0.00	5.5E-01	1.01	(0.98 - 1.04)	5.8E-01
Type 2 diabetes	38	22885922	44.31	1.9E-01	1.03	(0.99 - 1.06)	1.4E-01	1.03	(0.99 - 1.06)	1.8E-01	1.01	(0.96 - 1.07)	5.9E-01	1.01	(0.96 - 1.06)	7.3E-01	0.01	2.2E-01	0.97	(0.89 - 1.07)	5.6E-01
Ever smoked regularly	367	30643251	440.65	4.5E-03	1.08	(1.03 - 1.13)	**8.2E-04**	1.08	(1.03 - 1.13)	**2.3E-03**	1.04	(0.97 - 1.11)	3.1E-01	1.03	(0.96 - 1.10)	4.1E-01	0.00	7.6E-01	1.05	(0.85 - 1.29)	6.7E-01

Fracture at any bone site

	CochranQ		Inverse variance weighted fixed effects			Inverse variance weighted random effects			Weighted median			Penalized weighted median			Egger Intercept		Egger Estimate		
**Trait or disease**	Est	P	OR	95% CI	P	OR	95% CI	P	OR	95% CI	P	OR	95% CI	P	Beta	P	OR	95% CI	P
Continuous risk factor																			
**BMD-related parameters**																			
Decreased FN-BMD	382.23	**1.5E-54**	1.63	(1.56 - 1.70)	**3.6E-114**	1.63	(1.44 - 1.84)	**3.3E-15**	1.42	(1.31 - 1.54)	**3.5E-18**	1.37	(1.26 - 1.48)	**2.9E-15**	0.01	4.4E-01	1.41	(0.97 - 2.06)	7.4E-02
Decreased LS-BMD	396.11	**3.6E-58**	1.56	(1.50 - 1.62)	**1.1E-111**	1.56	(1.39 - 1.76)	**7.2E-14**	1.46	(1.35 - 1.58)	**5.5E-22**	1.39	(1.29 - 1.51)	**3.1E-17**	0.01	4.1E-01	1.36	(0.95 - 1.93)	9.0E-02
Decreased eBMD	1040.18	**5.8E-45**	1.65	(1.61 - 1.69)	**< 2.2E-308**	1.65	(1.59 - 1.71)	**3.0E-151**	1.76	(1.67 - 1.84)	**5.4E-115**	1.78	(1.69 - 1.88)	**1.0E-98**	0.00	8.5E-01	1.66	(1.55 - 1.76)	**< 2.2E-308**
**Other risk markers**																			
Early menopause	55.64	3.8E-01	1.07	(1.03 - 1.12)	**6.7E-04**	1.07	(1.03 - 1.12)	**9.0E-04**	1.08	(1.01 - 1.15)	1.6E-02	1.08	(1.01 - 1.15)	1.6E-02	0.00	8.8E-01	1.08	(0.98 - 1.20)	1.4E-01
Only females																			
Late puberty	155.75	**9.6E-04**	1.07	(1.02 - 1.12)	6.2E-03	1.07	(1.01 - 1.13)	2.5E-02	1.08	(0.99 - 1.16)	7.0E-02	1.08	(0.99 - 1.16)	7.0E-02	0.00	8.5E-01	1.05	(0.88 - 1.25)	6.1E-01
Only females																			
Onlly males																			
Decreased TSH	15.73	6.7E-01	0.99	(0.95 - 1.03)	6.2E-01	0.99	(0.95 - 1.03)	6.2E-01	1.01	(0.95 - 1.07)	8.1E-01	1.01	(0.95 - 1.07)	8.1E-01	0.00	8.1E-01	1.01	(0.88 - 1.15)	9.4E-01
Decreased grip strength/BW	268.17	**8.5E-12**	1.21	(1.13 - 1.30)	**2.4E-07**	1.21	(1.09 - 1.34)	**3.4E-04**	1.21	(1.07 - 1.35)	**1.4E-03**	1.21	(1.08 - 1.35)	**1.2E-03**	0.00	2.4E-01	0.93	(0.59 - 1.46)	7.5E-01
Low vitamin D levels	112.51	2.9E-02	0.99	(0.94 - 1.03)	5.4E-01	0.99	(0.94 - 1.04)	5.9E-01	1.00	(0.93 - 1.06)	8.9E-01	1.00	(0.93 - 1.06)	9.0E-01	0.00	1.8E-01	1.01	(0.95 - 1.08)	6.9E-01
Binary risk factor																			
Alzheimer´s disease	19.87	7.0E-01	1.00	(0.99 - 1.01)	6.5E-01	1.00	(0.99 - 1.01)	6.5E-01	1.00	(0.99 - 1.01)	5.5E-01	1.00	(0.99 - 1.01)	5.5E-01	0.00	7.6E-01	1.00	(0.99 - 1.01)	5.9E-01
Coronar heart disease	39.69	3.5E-01	1.01	(0.99 - 1.03)	4.1E-01	1.01	(0.99 - 1.03)	4.3E-01	1.02	(0.98 - 1.05)	3.5E-01	1.02	(0.98 - 1.05)	3.5E-01	0.00	2.6E-01	0.97	(0.90 - 1.04)	4.0E-01
Rheumatoid arhritis	77.13	3.2E-02	1.01	(1.00 - 1.02)	5.6E-02	1.01	(1.00 - 1.02)	1.0E-01	1.01	(0.99 - 1.02)	5.1E-01	1.01	(0.99 - 1.02)	5.0E-01	0.00	4.3E-01	1.00	(0.98 - 1.03)	8.7E-01
Inflamatory bowel disease	331.35	**4.5E-16**	1.00	(0.99 - 1.01)	6.7E-01	1.00	(0.99 - 1.01)	7.8E-01	1.01	(0.99 - 1.02)	3.1E-01	1.01	(1.00 - 1.02)	2.1E-01	0.00	8.9E-01	1.00	(0.98 - 1.02)	8.0E-01
Type 1 diabetes	13.87	7.4E-01	1.00	(0.99 - 1.00)	6.6E-01	1.00	(0.99 - 1.00)	6.6E-01	1.00	(0.99 - 1.01)	9.5E-01	1.00	(0.99 - 1.01)	9.5E-01	0.00	8.4E-01	1.00	(0.99 - 1.01)	8.5E-01
Type 2 diabetes	69.05	**1.1E-03**	1.00	(0.99 - 1.02)	5.1E-01	1.00	(0.98 - 1.03)	6.3E-01	1.02	(1.00 - 1.05)	7.4E-02	1.02	(1.00 - 1.05)	7.8E-02	-0.01	7.6E-02	1.05	(1.00 - 1.10)	6.9E-02
Ever smoked regularly	565.40	**1.5E-10**	1.05	(1.03 - 1.07)	**2.2E-06**	1.05	(1.02 - 1.07)	**1.4E-04**	1.05	(1.02 - 1.09)	**7.2E-04**	1.06	(1.03 - 1.10)	**9.3E-05**	0.00	2.3E-01	1.11	(1.01 - 1.23)	3.8E-02

Several different MR methods are displayed, including Inverse variance weighted, Weighted median, Penalized median and Egger regression. Odds ratio (OR) is for the risk of fracture per standard deviation (SD) change in the risk factor for continuous trait or risk of fracture per doubling of odds of disease susceptibility for binary factors. For ever smoked regularly, the ORs are expressed per 0.5 unit increase in log odds of ever smoking regularly with a 1 SD increase in genetically predicted smoking initiation corresponding to a 10% increased risk of smoking (PMID30643251 + PMID33253141).

Findings that remain associated after correction for multiple testing (0.05/15; $\alpha<3.3\times10^{-3}$) are given in bold. Grip strength is given as grip strength per body weight (BW). SD for grip strength is given for kg grip strength per kg in body weight and was estimated in the UK-Biobank to be 0.127. NA=not available; BMD=bone mineral density. Estimates for the association with hip fracture is from the present hip fracture GWAS while the estimates from any fracture is from Morris *et al* Nature Genetics 2019 (PMID30598549). TSH = Thyroid-stimulating hormone, FN = femoral neck, LS = lumbar spine, eBMD = estimated BMD in the heel using ultrasound.

Table S8 Association for femoral neck bone mineral density GWAS significant loci with hip fracture risk. Related to Table 1 and Fig 1.

			FN-BMD				Hip fracture			
**SNP**	**EA**	**OA**	**EAF**	**Beta**	**SE**	**P**	**EAF**	**OR**	**95%CI**	**P**
rs736825	C	G	0.56	0.04	0.01	1.1E-09	0.59	0.92	(0.90 - 0.95)	**8.9E-08**
rs1566045	C	T	0.20	0.06	0.01	1.9E-22	0.19	0.90	(0.87 - 0.94)	**1.1E-07**
rs1366594	A	C	0.54	0.08	0.00	4.5E-61	0.55	0.93	(0.90 - 0.96)	**8.1E-07**
rs4792909	T	G	0.37	0.04	0.01	2.0E-11	0.41	0.93	(0.90 - 0.96)	**1.5E-06**
rs1286083	C	T	0.19	0.05	0.01	2.0E-15	0.17	0.91	(0.88 - 0.95)	**3.0E-06**
rs7108738	G	T	0.17	0.08	0.01	1.1E-32	0.22	0.92	(0.89 - 0.96)	**9.5E-06**
rs4796995	A	G	0.63	0.03	0.01	4.9E-08	0.63	0.94	(0.91 - 0.96)	**1.2E-05**
rs11623869	G	T	0.65	0.04	0.00	5.2E-16	0.66	0.94	(0.91 - 0.97)	**6.7E-05**
rs9466056	G	A	0.62	0.04	0.01	2.7E-13	0.63	0.94	(0.92 - 0.97)	**1.1E-04**
rs430727	C	T	0.52	0.06	0.01	4.4E-25	0.56	0.95	(0.92 - 0.97)	**1.4E-04**
rs6532023	T	G	0.34	0.06	0.01	5.0E-26	0.32	0.95	(0.92 - 0.97)	**3.3E-04**
rs6959212	C	T	0.68	0.04	0.01	1.2E-13	0.68	0.95	(0.92 - 0.98)	**3.9E-04**
rs3790160	T	C	0.50	0.04	0.01	3.6E-12	0.49	0.95	(0.93 - 0.98)	**1.4E-03**
rs7521902	C	A	0.69	0.04	0.01	2.9E-09	0.75	0.95	(0.92 - 0.98)	**1.5E-03**
rs7751941	G	A	0.79	0.04	0.01	1.6E-09	0.79	0.95	(0.91 - 0.98)	**3.7E-03**
rs4790881	A	C	0.69	0.05	0.01	9.8E-19	0.69	0.96	(0.93 - 0.99)	**4.0E-03**
rs7953528	A	T	0.18	0.05	0.01	1.9E-12	0.17	0.95	(0.91 - 0.99)	**6.9E-03**
rs1373004	G	T	0.87	0.04	0.01	1.5E-08	0.88	0.94	(0.90 - 0.98)	**7.1E-03**
rs2062377	T	A	0.43	0.06	0.01	9.1E-25	0.43	0.96	(0.93 - 0.99)	**8.6E-03**
rs3736228	C	T	0.84	0.05	0.01	4.8E-11	0.87	0.94	(0.90 - 0.99)	**1.0E-02**
rs6426749	C	G	0.17	0.11	0.01	7.4E-57	0.15	0.95	(0.91 - 0.99)	**1.2E-02**
rs13336428	G	A	0.57	0.04	0.00	1.5E-16	0.59	0.97	(0.94 - 1.00)	**2.3E-02**
rs9921222	C	T	0.52	0.04	0.01	5.2E-12	0.52	0.97	(0.94 - 1.00)	**2.4E-02**
rs4985155	G	A	0.33	0.03	0.00	1.7E-10	0.34	0.97	(0.94 - 1.00)	**2.8E-02**
rs7812088	A	G	0.13	0.05	0.01	7.3E-09	0.13	0.96	(0.92 - 1.00)	**4.0E-02**
rs10048146	A	G	0.80	0.05	0.01	1.0E-14	0.80	0.97	(0.93 - 1.00)	5.3E-02
rs4727338	C	G	0.67	0.08	0.01	8.1E-48	0.68	0.97	(0.94 - 1.00)	5.4E-02
rs7851693	C	G	0.64	0.05	0.01	3.4E-22	0.63	0.97	(0.94 - 1.00)	6.0E-02
rs7932354	T	C	0.31	0.05	0.01	5.1E-18	0.31	0.97	(0.94 - 1.00)	6.8E-02
rs4869742	C	T	0.69	0.05	0.01	4.2E-18	0.72	0.97	(0.93 - 1.01)	9.3E-02
rs7217932	A	G	0.46	0.03	0.00	1.9E-11	0.48	0.98	(0.95 - 1.01)	1.2E-01
rs2887571	G	A	0.24	0.03	0.01	6.5E-09	0.23	0.97	(0.94 - 1.01)	1.3E-01
rs163879	C	T	0.32	0.03	0.01	2.1E-08	0.35	0.98	(0.95 - 1.01)	1.8E-01
rs1346004	G	A	0.50	0.05	0.00	1.1E-25	0.49	0.98	(0.95 - 1.01)	2.0E-01
rs7584262	T	C	0.23	0.04	0.01	1.3E-09	0.23	0.99	(0.95 - 1.02)	4.0E-01
rs1053051	C	T	0.48	0.03	0.00	9.6E-10	0.48	0.99	(0.96 - 1.02)	4.4E-01
rs9533090	C	T	0.51	0.05	0.01	4.9E-23	0.49	0.99	(0.96 - 1.02)	4.5E-01
rs17040773	A	C	0.76	0.04	0.01	1.5E-09	0.79	0.99	(0.95 - 1.02)	4.7E-01
rs1026364	T	G	0.37	0.03	0.00	4.1E-10	0.39	1.01	(0.98 - 1.04)	5.7E-01
rs7084921	T	C	0.39	0.03	0.00	9.0E-10	0.37	0.99	(0.96 - 1.02)	5.9E-01
rs13204965	A	C	0.76	0.04	0.01	8.1E-12	0.76	0.99	(0.96 - 1.02)	5.9E-01
rs479336	G	T	0.26	0.04	0.01	8.5E-15	0.26	0.99	(0.96 - 1.02)	5.9E-01
rs3801387	G	A	0.26	0.08	0.01	5.0E-40	0.26	1.01	(0.98 - 1.04)	6.0E-01
rs884205	C	A	0.73	0.04	0.01	3.2E-10	0.73	0.99	(0.96 - 1.03)	6.5E-01
rs3755955	G	A	0.84	0.06	0.01	1.5E-14	0.85	0.99	(0.95 - 1.03)	7.0E-01
rs12407028	T	C	0.60	0.05	0.01	3.4E-23	0.58	1.00	(0.97 - 1.02)	7.4E-01
rs2016266	G	A	0.32	0.03	0.00	3.7E-10	0.32	1.00	(0.97 - 1.03)	7.8E-01

EA = effect allele, OA= other allele. Effect estimates for femoral neck bone mineral density (FN-BMD) are derived from Estrada et al (PMID 2250442).

Table S9 Association for Alzheimer's disease GWAS significant loci with hip fracture risk. Related to Fig 1.

					Alzheimer's disease						Hip fracture					
SNP	POS	Gene	EA	OA	EAF	Beta	SE	OR	95%CI	P	EAF	Beta	SE	OR	95%CI	P
rs429358	chr19:44908684	APOE	C	T	0.22	1.20	0.02	3.32	(3.20 - 3.45)	1.2E-881	0.17	0.13	0.02	1.14	(1.10 - 1.19)	3.8E-11
rs9473117	chr6:47463548	CD2AP	C	A	0.28	0.09	0.01	1.09	(1.06 - 1.12)	1.2E-10	0.26	0.04	0.02	1.04	(1.00 - 1.07)	2.9E-02
rs3851179	chr11:86157598	EED	C	T	0.64	0.13	0.01	1.14	(1.11 - 1.16)	6.0E-25	0.64	0.03	0.02	1.03	(1.00 - 1.06)	6.3E-02
rs12539172	chr7:100494172	NYAP1	C	T	0.70	0.08	0.01	1.09	(1.06 - 1.12)	9.3E-10	0.69	0.02	0.02	1.03	(0.99 - 1.06)	1.2E-01
rs9331896	chr8:27610169	CLU	T	C	0.61	0.13	0.01	1.14	(1.11 - 1.16)	4.6E-24	0.58	-0.02	0.02	0.98	(0.95 - 1.01)	1.3E-01
rs3740688	chr11:47358789	SPI1	T	G	0.55	0.08	0.01	1.09	(1.06 - 1.11)	5.4E-13	0.53	0.02	0.02	1.02	(0.99 - 1.05)	1.5E-01
rs6733839	chr2:127135234	LOC105373605	T	C	0.41	0.18	0.01	1.20	(1.17 - 1.23)	2.1E-44	0.40	0.02	0.02	1.02	(0.99 - 1.05)	1.8E-01
rs4844610	chr1:207629207	CR1	A	C	0.19	0.16	0.02	1.17	(1.14 - 1.21)	3.6E-24	0.19	0.02	0.02	1.02	(0.99 - 1.06)	2.3E-01
rs9271058	chr6:32607629	HLA-DRB1	A	T	0.27	0.10	0.01	1.10	(1.07 - 1.13)	1.4E-11	0.29	0.02	0.02	1.02	(0.98 - 1.06)	3.1E-01
rs593742	chr15:58753575	ADAM10	A	G	0.71	0.07	0.01	1.08	(1.05 - 1.10)	6.8E-09	0.66	0.02	0.02	1.02	(0.98 - 1.05)	3.3E-01
rs6024870	chr20:56422512	CASS4	G	A	0.91	0.13	0.02	1.14	(1.09 - 1.19)	3.5E-08	0.93	-0.02	0.03	0.98	(0.92 - 1.04)	4.2E-01
rs73223431	chr8:27362470	PTK2B	T	C	0.37	0.10	0.01	1.10	(1.07 - 1.13)	6.3E-14	0.37	0.01	0.02	1.01	(0.98 - 1.04)	4.7E-01
rs11218343	chr11:121564878	SORL1	T	C	0.96	0.22	0.03	1.25	(1.17 - 1.33)	2.9E-12	0.97	0.03	0.04	1.03	(0.95 - 1.11)	4.8E-01
rs12881735	chr14:92466484	SLC24A4	T	C	0.78	0.08	0.01	1.09	(1.06 - 1.12)	7.4E-09	0.78	0.01	0.02	1.01	(0.98 - 1.05)	5.5E-01
rs3752246	chr19:1056493	ABCA7	G	C	0.18	0.14	0.02	1.15	(1.11 - 1.19)	3.1E-16	0.16	0.01	0.02	1.01	(0.97 - 1.05)	5.6E-01
rs2830500	chr21:26784537	ADAMTS1	C	A	0.69	0.07	0.01	1.08	(1.05 - 1.10)	2.6E-08	0.70	0.01	0.02	1.01	(0.98 - 1.04)	5.7E-01
rs7920721	chr10:11678309	ECHDC3	G	A	0.39	0.08	0.01	1.08	(1.05 - 1.11)	2.3E-09	0.38	-0.01	0.02	0.99	(0.96 - 1.02)	6.5E-01
rs7185636	chr16:19796841	IQCK	T	C	0.82	0.08	0.01	1.09	(1.06 - 1.12)	2.4E-08	0.83	-0.01	0.02	0.99	(0.95 - 1.04)	6.9E-01
rs10808026	chr7:143402040	EPHA1	C	A	0.80	0.11	0.02	1.11	(1.08 - 1.15)	1.3E-10	0.80	0.01	0.02	1.01	(0.97 - 1.04)	7.3E-01
rs62039712	chr16:79321960	MAF	A	G	0.12	0.15	0.03	1.16	(1.10 - 1.22)	3.7E-08	0.12	0.01	0.02	1.01	(0.96 - 1.06)	7.3E-01
rs17125924	chr14:52924962	FERMT2	G	A	0.09	0.13	0.02	1.14	(1.09 - 1.19)	1.4E-09	0.10	-0.01	0.02	0.99	(0.94 - 1.04)	7.4E-01
rs114812713	chr6:41066261	OARD1	C	G	0.03	0.28	0.04	1.32	(1.23 - 1.42)	2.1E-13	0.03	-0.01	0.06	0.99	(0.88 - 1.10)	7.9E-01
rs138190086	chr17:63460787	CYB561	A	G	0.02	0.28	0.05	1.32	(1.20 - 1.45)	7.5E-09	0.02	0.01	0.06	1.01	(0.90 - 1.13)	9.1E-01
rs10933431	chr2:233117202	INPP5D	C	G	0.78	0.09	0.02	1.10	(1.07 - 1.13)	3.4E-09	0.78	0.00	0.02	1.00	(0.96 - 1.03)	9.5E-01
rs7933202	chr11:60169453	MS4A6A	A	C	0.61	0.12	0.01	1.12	(1.10 - 1.15)	1.9E-19	0.64	0.00	0.02	1.00	(0.97 - 1.03)	9.6E-01

EA = effect allele, OA= other allele. Effect estimates for Alzheimer's disease are derived from Kunkle et al (PMID 30820047).

Table S10 Multivariable association for Alzheimer's disease and falls on risk of hip fractures. Related to Fig 1.

		Heterogeneity		Inverse variance weighted Fixed effects model			Inverse variance weighted Random effects model			MR-Egger Regression					Multivariable/Weighted Median		
										Intercept		Slope					
Risk factor	N SNPs	Cochran's Q	P	OR	95% CI	P	OR	95% CI	P	Beta	P	OR	95% CI	P	OR	95% CI	P
**Individual associations**																	
Alzheimer's disease	24	17.59	7.8E-01	1.07	(1.05 - 1.10)	2.8E-12	1.07	(1.05 - 1.10)	2.8E-12	0.00	6.5E-01	1.08	(1.05 - 1.11)	3.2E-09	1.08	(1.06 - 1.10)	2.8E-11
Falls	3	5.22	7.4E-02	1.01	(0.63 - 1.61)	9.8E-01	1.01	(0.47 - 2.14)	9.9E-01						1.24	(0.64 - 2.43)	5.2E-01
**Multivariable associations**																	
Alzheimer's disease	27	22.67	6.0E-01	1.07	(1.05 - 1.10)	5.7E-11	1.07	(1.05 - 1.10)	5.7E-11	0.00	4.4E-01	1.08	(1.05 - 1.10)	1.5E-09	1.07	(1.05 - 1.10)	4.8E-08
Falls				1.09	(0.72 - 1.64)	6.8E-01	1.09	(0.72 - 1.64)	6.8E-01			1.11	(0.74 - 1.68)	6.1E-01	1.28	(0.69 - 2.38)	4.3E-01

Genetic instrument for Alzheimer's disease were derived from Kunkle et al (PMID 30820047) while genetic instruments for falls were derived from Trajanaskova et al (PMID 32999390)
